# EuropeaN Energy balance Research to prevent excessive weight Gain among Youth (ENERGY) project: Design and methodology of the ENERGY cross-sectional survey

**DOI:** 10.1186/1471-2458-11-65

**Published:** 2011-01-31

**Authors:** Maartje M van Stralen, Saskia J te Velde, Amika S Singh, Ilse De Bourdeaudhuij, Marloes K Martens, Maria van der Sluis, Yannis Manios, Evangelia Grammatikaki, Mai JM Chinapaw¹, Lea Maes, Elling Bere, Jorgen Jensen, Luis Moreno, Nataša Jan, Dénes Molnár, Helen Moore, Johannes Brug

**Affiliations:** 1 Department of Public and Occupational Health, VU University Medical Center, EMGO Institute for Health and Care Research, Amsterdam, the Netherlands; 2 Department of Epidemiology and Biostatistics, VU University Medical Center, EMGO Institute for Health and Care Research, Amsterdam, the Netherlands; 3 Department of Movement and Sport Sciences, Ghent University, Ghent, Belgium; 4 ResCon, Amsterdam, the Netherlands; 5 Department of Nutrition and Dietetics, Harokopio University, Athens, Greece; 6 Department of Public Health, Ghent University, Ghent, Belgium; 7 Faculty of Health and Sport, University of Agder, Kristiansand, Norway; 8 Institute of Food and Resource Economics, University of Copenhagen, Copenhagen, Denmark; 9 GENUD (Growth, Exercise, Nutrition and Development) Research Group. E.U. Ciencias de la Salud, Universidad de Zaragoza, 50009 Zaragoza, Spain; 10 Slovenian Heart Foundation, Ljubljana, Slovenia; 11 Department of Paediatrics, Pecs University, Pecs, Hungary; 12 Obesity Related Behaviours Research Group, Durham University, Durham, UK

## Abstract

**Background:**

Obesity treatment is by large ineffective long term, and more emphasis on the prevention of excessive weight gain in childhood and adolescence is warranted. To inform energy balance related behaviour (EBRB) change interventions, insight in the potential personal, family and school environmental correlates of these behaviours is needed. Studies on such multilevel correlates of EBRB among schoolchildren in Europe are lacking. The ENERGY survey aims to (1) provide up-to-date prevalence rates of measured overweight, obesity, self-reported engagement in EBRBs, and objective accelerometer-based assessment of physical activity and sedentary behaviour and blood-sample biomarkers of metabolic function in countries in different regions of Europe, (2) to identify personal, family and school environmental correlates of these EBRBs. This paper describes the design, methodology and protocol of the survey.

**Method/Design:**

A school-based cross-sectional survey was carried out in 2010 in seven different European countries; Belgium, Greece, Hungary, the Netherlands, Norway, Slovenia, and Spain. The survey included measurements of anthropometrics, child, parent and school-staff questionnaires, and school observations to measure and assess outcomes (i.e. height, weight, and waist circumference), EBRBs and potential personal, family and school environmental correlates of these behaviours including the social-cultural, physical, political, and economic environmental factors. In addition, a selection of countries conducted accelerometer measurements to objectively assess physical activity and sedentary behaviour, and collected blood samples to assess several biomarkers of metabolic function.

**Discussion:**

The ENERGY survey is a comprehensive cross-sectional study measuring anthropometrics and biomarkers as well as assessing a range of EBRBs and their potential correlates at the personal, family and school level, among 10-12 year old children in seven European countries. This study will result in a unique dataset, enabling cross country comparisons in overweight, obesity, risk behaviours for these conditions as well as the correlates of engagement in these risk behaviours.

## Background

Despite large differences between countries and regions [1], prevalence of overweight and obesity among children and adolescents has risen across Europe in recent decades [1,2]. The most recent reports show that approximately 20% of the children in several European countries are overweight or obese [1,2]. Childhood overweight and obesity track into adulthood [3] and are linked to ill health [4]. Obesity treatment is by large ineffective long term, and more emphasis on the prevention of excessive weight gain in childhood and adolescence is warranted.

Although genetic factors may influence the susceptibility of individuals to gain weight [5], there is consensus that changes in lifestyle behaviour are driving the obesity epidemic [6] rather than changes in biologic or genetic factors. A long-term positive energy balance - i.e. energy intake through food intake exceeds energy expenditure for body functions and physical activity - leads to storage of excess energy as fat, leading to weight gain, and eventually to the development of overweight and obesity. Prevention of unnecessary weight gain should thus target modifiable energy intake and energy expenditure behaviours, i.e. physical activity, sedentary, and dietary behaviours, also referred to as energy balance related behaviours (EBRBs). Recent research and reviews of the literature indicate that among schoolchildren specific EBRB are of specific relevance for obesity prevention [7-9]. These behaviours concern screen viewing behaviour (TV viewing and sedentary computer activities), intake of sugar sweetened beverages and breakfast consumption, and daily activities, i.e. active commuting to school, physical activity during recess, participation in sports and recreational physical activity. Additionally, recent evidence suggests that sleeping habits may also be relevant for energy balance [10]. According to socio ecological and cognitive behavioural models, to inform EBRB change interventions, we need insight in the potential personal, family and school environmental correlates of these behaviours [11-14].

Studies on such multilevel correlates of EBRBs among schoolchildren in Europe are lacking. As part of the European Commission-funded "EuropeaN Energy balance Research to prevent excessive weight Gain among Youth" (ENERGY)-project [12], a cross-European school-based, family-involved survey study was conducted. This study aims to (1) provide up-to-date prevalence rates of measured overweight, obesity, self reported engagement in EBRBs, and objective accelerometer assessment of physical activity and sedentary behaviour and blood-sample biomarkers of metabolic function in countries in different regions of Europe, (2) to identify personal, family-environmental and school-environmental correlates of these EBRBs. This paper describes the design, methodology and protocol of the cross-sectional study.

## Methods/Design

A school-based cross-sectional survey was carried out between March and July 2010 in seven different European countries; Belgium, Greece, Hungary, the Netherlands, Norway, Slovenia, and Spain. The survey included anthropometric measurements, child questionnaires, parent questionnaires, school-staff questionnaires and school observations to measure EBRBs and potential individual and environmental correlates of these behaviours.

The project adhered to the Helsinki Declaration and the conventions of the Council of Europe on human rights and biomedicine. All participating countries obtained ethical clearance from the relevant ethical committees and ministries; in Belgium the survey was approved by the Medical Ethics Committee of the University Hospital Ghent; in Greece the survey was approved by the Bioethics Committee of Harokopio University; in Hungary the survey was approved by the Scientific and Ethics Committee of Health Sciences Council; in the Netherlands the survey was approved by the Medical Ethics Committee of the VU University medical center; in Norway the survey was approved by the National Committees for Research Ethics in Norway; in Slovenia the survey was approved by the National Medical Ethics Committee of the Republic of Slovenia; and in Spain the survey was approved by Clinical Research Ethics Committee of the Government of Aragón. Furthermore, research permission was, if necessary, obtained from local school authorities (local school boards and/or headmasters).

### Sampling procedures and recruitment

Each country was represented by a local partner institute and each partner was responsible for the data collection in that country, with one of the partners acting as the overall coordinating centre. The procedure for sampling, data collection, and data handling for all parts of the survey was the same in all countries according to standardised protocols (see additional file 1 for the fieldwork protocol on recruitment and data collection).

The survey was carried out at schools among 10-12 year old children. Based on previous cross-European studies (e.g. the Pro-Children study [15]) a minimum sample of 1,000 school-children per country and one parent (the main caretaker) for each child was aimed. This minimum was required to enable analyses of the associations between correlates and specific EBRBs and to allow for within-country analyses as well as between-country comparisons. A total of 7,000 observations is sufficient to run prediction models with at least 10 predicting variables. In addition, it was calculated that with a power of 90% a between country difference of 5% in overweight prevalence rates will be detected as a significant difference (assuming an average overweight rate of 15%). For each country, the aim was to include 20 schools and 2 classes per school, resulting in approximately 50 children per school. In order to recruit at least 1,000 children it was necessary to over-sample. With an anticipated non-response rate of 10%, it was decided that approximately 1,100 schoolchildren were needed.

Sampling was national in Greece, Hungary, the Netherlands, and Slovenia. In Spain, schools in the region of Aragón were selected, Belgium selected schools from Flanders, the northern Dutch-speaking part of Belgium, and Norway selected schools from the southern regions of the country. Due to the differences in population distribution within the different regions, the sampling of schools was random, multi-staged, and stratified by degree of urbanization and consisted of 7 steps. First, the percentage of people living in municipalities with more than 20,000 inhabitants was calculated (Steps 1), then tertiles were formed based on this urbanization degree (Step 2), after which one province was randomly selected from each tertile (Step 3). From each of the three selected provinces one municipality was randomly selected (step 4). For these three selected municipalities, a list of all schools (Step 5) were created and sent to the coordinating partner. The coordinator then randomly selected the schools (Step 6). Based on the random selection of schools the countries started recruiting the schools following the provided rank order. If inclusion was insufficient additional schools were recruited from municipalities (i.e. in Belgium, Greece, and Hungary) or regions (i.e. in the Netherlands, Norway) (Step 7).

A school recruitment letter was sent to the headmaster or principal of the sampled schools, followed by a personal call, and if recruited a personal visit in order to answer any remaining questions and to explain the timeframe of the survey in their school. Following the school's agreement, parents received a letter explaining the study purpose and were asked for written consent for their child's and their own participation in the ENERGY-project. Passive informed consent was allowed in the Netherlands. If no parental consent was available for a child, the child did not participate in the study.

Accelerometer data for the assessment of physical activity and sedentary behaviour was collected in a selection of schools in Belgium, Greece, Hungary, and the Netherlands. The goal was to collect accelerometer data from at least 200 children per country from four schools (50 students per school). The selection of schools was balanced across the three cities as much as possible (selected from the three tertiles). The protocol of the accelerometer data collection is described in more detail elsewhere (Yıldırım, Verloigne, De Bourdeaudhuij, Androutsos, Manios, Felso, Kovacs, Doessegger, Bringolf-Isler, Te Velde, Chinapaw, unpublished data). Blood samples were collected in a selection of schools in Hungary and the Netherlands. The goal was to collect data from approximately 200 children who also wore accelerometers.

Table 1 shows the recruitment rate and response rate on the school, child and parent level.

**Table 1:** Overview of data collection and response rates per country

	Belgium	Greece	Hungary	Netherlands	Norway	Slovenia	Spain	Total
**School level**								
N	26	37	29	23	21	15	24	175
Response rate (%)	29%	54%	71%	5%	36%	100%	72%	-
No. of completed audits	26	34	32^1^	23	21	15	21	172
No. of completed SMQ	19	37	29	15	20	15	21	156

**Child level**								
No. completed Questionnaires	1003	1077	1022	926	1004	1178	1024	7234
Response rate Questionnaire	82%	94%	100%	89%	99%	98%	97%	-
No. completed Anthropometry	1005	1077	1022	898	980	1146	1024	7152
Response rate (anthropometry)	82%	94%	33.2%	87%	45%	96%	97%	-
No. of measured accelerometry	194	215	200	199	N/A	N/A	N/A	808
Response rate (Accelerometry)	84%	97%	19.9%	85%	n/a	n/a	n/a	-

**Parent level**								
No. of completed questionnaire	763	1008	932	404	903	1028	964	6002
Response rate Questionnaire	62%	83%	91%	44%	89%	87%	94%	-

N/A: Not applicable; SMQ = School Management Questionnaire

^1. ^In Hungary some schools had more than one location; therefore the number of audits is larger than the number of schools.

### Data collection

The children confidentially completed the child questionnaire during one school hour in the presence of the research assistant or project worker. Questionnaire administration did not take place on Mondays in order to avoid that weekend days were reported in answering the 24h recall questions. A research assistant guided the completion of the questionnaire and answered any questions from the participating children. In addition, anthropometric measurements were conducted. In order to measure the school environment, two observers independently conducted audits of the schools, school cafeterias, and school recreation facilities. A brief interview with a cafeteria manager and/or school representative was part of the audit. In addition a school representative at each school was asked to complete a school management questionnaire about the availability of food and physical activity related facilities within the school environment and about school policies. During the school visit, the children of a selection of schools in Greece, the Netherlands, Hungary, and Belgium were asked to wear accelerometers for six consecutive days. The children brought the devices back to school at least six days later and returned it to their teacher. From the children that were selected for wearing accelerometers in the Netherlands and Hungary, also blood samples were taken after an overnight fast. After these measurements, the children received breakfast.

The children received the parent questionnaire in a closed envelop to take home for completion by one of their parents. Completed parent questionnaires were brought back to the school by the children and were collected by the teacher.

## Measurement instruments

The following measurement instruments and measures were administered: child questionnaire, parent questionnaire, accelerometers, anthropometric measures, school management questionnaire, interview with those responsible for the cafeteria/food retail and the vending machine, and a school environment audit instrument.

### Development of measurement instruments

The selection of EBRBs and correlates measured in the questionnaires were based on the results of the literature reviews and secondary data analyses conducted in the earlier studies of the ENERGY project [12]. The ENERGY-questionnaires were developed based on and using items from existing validated and relevant European questionnaires. If no established or valid questionnaires were available the ENERGY-team used existing items from relevant earlier or ongoing projects. If no such questionnaires or items were available, new items or questionnaire parts were constructed.

Since the measurement instruments had to be standardised for all participating countries, the child, parent, and school management questionnaires and staff interviews were developed in English, and translated into the language of each participating country. The English version of the child and parent questionnaire is shown on the ENERGY website (see http://projectenergy.eu). The child and parent questionnaire were then back-translated by an official translator in order to detect any potential differences between the two. In case of differences, these were discussed within the ENERGY team and adaptations were made accordingly.

#### Pre-testing of measurement instruments

The child and parent questionnaires, audit instrument, school management questionnaire and staff interview were then first pre-tested among small samples in all participating countries to examine comprehensibility and duration of completion. Based on these results, the original version was adapted if necessary.

For the child questionnaire pre-test, five to ten 10-12 year old children from one primary school were requested to complete the questionnaire. In structured focus group interviews following a predefined check-list, the pupils were asked for their general opinion about the questionnaire, the comprehensibility and feasibility of the questionnaire and their opinion about the design of the questionnaire. In the pre-test of the parent questionnaire, a total of five to ten parents who had children aged 10-12 years old were recruited via schools. A telephone interview with the parents was conducted following a checklist in order to assess the parents' general opinion about the questionnaire, the comprehensibility and feasibility of the questionnaire and their opinion about its design. For the pre-testing of the school management questionnaire, two to three representatives of the school management (e.g. headmasters, adjunct head-masters) were asked to complete the questionnaire. Subsequently, they were interviewed by telephone about how the questions could be improved for content or phrasing, about the comprehensibility and feasibility of the questionnaire and about the design of the questionnaire. The audit instrument was pre-tested by conducting observations at one school by two observers separately. Two researchers per country were asked for their opinion and experience with regard to completing the audit instrument. Important aspects of the pre-test were the completeness of the forms and feasibility. One researcher performed the interview with the person responsible for the cafeteria/school shop and the vending machines. After the interview, the person(s) of the cafeteria/school shop and vending machine were asked to evaluate the interview.

After the pre-tests of the child questionnaire, parent questionnaire, school management questionnaire, staff interview and audit instrument, the measurements were adapted based on the results of the pre-tests.

#### Reliability and construct validity test

The reliability and content validity of the child and parent questionnaires were tested separately in all participating countries, in five schools per country using approximately 100 children and 50 parents per country for the reliability study and 15 children and 20 parents for the construct validity study. For the reliability test, a test‐retest design was used by comparing data from two completions of the questionnaire conducted one week apart, on the same weekday, under equal circumstances. To determine the (construct) validity of the questionnaire, the degree of agreement between the questionnaire and information from cognitive interviews administered after completion of the first questionnaire was assessed. The results of the test-retest study is described in more detail elsewhere (Singh, Chinapaw, Terwee, Vik, van Lippevelde, Fernandez, Kovacs, Jan, Manios, van der Sluis and Brug, unpublished data.; Singh, Vik, Chinapaw, Verloigne, Fernandez, Kovacs, Jan, Manios, Martens, and Brug, unpublished data).

### Child questionnaire

The child questionnaire assessed self-reported levels of EBRBs, and personal and family environmental correlates.

#### Energy Balance Related Behaviours

Table 2 shows the EBRBs (i.e. dietary behaviours, physical activity, sedentary, and dieting behaviour) assessed in the child questionnaire. Dietary intake was assessed with food frequency questions referring to a general week and a 24-hour recall question [16]. Physical activity behaviours (i.e., commuting to school, activity during recess, sports/physical activity behaviour during leisure time) and sedentary behaviour (i.e., television viewing and computer time) were assessed with frequency questions referring to a general week and a 24-hour recall question [16]. In addition, dieting behaviour was assessed by two items, one using a frequency score of the last year and one on the current dieting behaviour informed by the restrained eating questions from the Three Factor Eating Questionnaire [17].

**Table 2:** Dietary, Physical activity, sedentary, sleeping and dieting behaviour measured in the child en parent questionnaire

	Child Questionnaire	Parent Questionnaire
**Dietary behaviour**		
*Sugar-sweetened beverage consumption*	1. How many times a week do you usually drink fizzy drinks and fruit squash?	1. How many times a week on average do you drink soft drinks?
	2. On a day that you drink fizzy drinks and fruit squash, how many glasses, cans or bottles do you drink on such a day?	2. On a day that you drink soft drinks, how many glasses, cans or bottles do you drink?
	3. How many fizzy drinks or fruit squash did you drink yesterday?	
*Fruit Juice consumption*	1. How many times a week do you usually drink fruit juices	1 How many times a week on average do you drink fruit juices?
	2. On a day that you drink fruit juices, how many glasses or cartons do you drink on such a day?	2 On a day that you drink fruit juices, how many glasses or cartons do you drink?
	3. How many fruit juices did you drink yesterday?	
*Breakfast consumption*	1. From Monday to Friday during school weeks, on how many days do you usually eat breakfast?	1. From Monday to Friday, how many times do you usually eat breakfast?
	2. On how many days in the weekend (Saturday and Sunday) do you usually eat breakfast?	2. How many times do you usually eat breakfast on the weekend?
	3. Did you eat breakfast yesterday?	
*Meal patterns*	1. Did you eat lunch yesterday?	1. How often do you and/or your spouse/partner have breakfast together with your child?
	2. Did you eat dinner yesterday?	2. How often do you and/or your spouse/partner have lunch together with your child?
	3. Did you eat anything between meals yesterday?	3. How often do you and/or your spouse/partner have dinner together with your child?

**Physical activity behaviour**		
*Commuting to school/work*	1. How many days do you usually bike to school?	1. How many days do you travel by car to work?
	2. If you bike to school, how long does it take you to bike to school?	2. How many days do you usually use public transport (bus, tram, metro) to go to work?
	3. How many days a week do you usually walk to school?	3. How many days do you usually cycle to work or to the public transport station?
	4. If you walk to school, how long does it take you to walk to school?	4. If you cycle, how long does it take you to cycle to work or the public transport station?
	5. How many days do you usually travel by car to school?	5. How many days do you usually walk to work or to the public transport station?
	6. How many days do you usually travel by public transport (bus, school bus, tram, metro) to school?	6. If you walk, how long does it take you to cycle to work or the public transport station?
	7. How did you go to school today?	
*Activity during recess*	1. What do you usually do during breaks at school?	
*Physical activity/sports in leisure time*	1. In a total week how many hours do you do this sport (sport1)?	1. About how many days a week do you usually participate in physical activities/sports in your leisure time?
	2. In a total week how many hours do you do this sport (sport2)?	2. About how much time a week do you participate in physical activities/sports in your leisure time?
	3. How many hours of sports did you do yesterday?	

**Sedentary behaviour**		
*Television viewing*	1. About how many hours a day do you usually watch television in your free time?	1. About how many hours a day do you usually watch television (including DVDs and videos) in your free time?
	2. About how many hours did you watch television yesterday?	
*Computer time*	1. About how many hours a day do you usually play games on a computer, or use your computer for leisure activities in your free time?	1. About how many hours a day do you usually use your computer for activities like chatting online, internet, emailing or playing games on a computer, games console (e.g. Playstation, Xbox, GameCube) during leisure time?
	2. About how many hours did you play games on a computer, games console or use your computer for leisure activities yesterday?	
*Mobile phone use*		1. About how many hours a day do you usually use your mobile phone for calling, texting, playing games or surfing on the internet during leisure time?

**Dieting behaviour**		
*Dieting*	1. How often have you tried to get slimmer/thinner during the last year?	1. I deliberately have smaller helpings as a means of controlling my weight.
	2. Do you try to get slimmer or thinner right now?	2. I do not eat certain foods because they make me fat.
		3. On a scale of 1 to 8, where 1 means no restraint in eating (eating, as much as you want, whenever you want it) and 8 means total restraint (constantly limiting food intake and never "giving in"), what rating would you give yourself?

**Sleeping behaviour**		
*Sleeping behaviour child*		1. How many hours of sleep does your child usually have during the night?

#### Personal variables

Child characteristics, such as gender, date of birth, ethnicity, and family status of the child were assessed using one question. Attitude, knowledge of health promotion recommendation with regard to the specific EBRB, self-efficacy, health beliefs, preference/liking and habit strength were assessed for all dietary behaviours, physical activity/sport behaviours, and television viewing using one question with a five-point answer format. These items were informed by the Pro Children questionnaire [18]. Table 3 shows the assessed personal variables, their description and the questionnaire items.

**Table 3:** Measurement items of each specific correlates per EBRB of the Child Questionnaire

	Description	Soft drink	Fruit juice	Breakfast	Physical activity/Sports	TV viewing
**Personal correlates**						

Attitude^1^	Does the child consider the behaviour as 'bad' or 'good'	I think that drinking fizzy drinks or fruit squash is (very bad (-2)- very good (+2))	I think that drinking fruit drinks is (very bad (-2)- very good (+2))	I think that eating breakfast is (very bad (-2)- very good (+2))	I think that physical activity/sports is (very bad (-2)- very good (+2))	I think that watching TV is (very bad (-2)- very good (+2))

Knowledge^1^	Does the child know the recommendation with regard to the behaviour is		I think it is recommended for children my age Not to drink fruit juices at all (1), To drink not more than one glass a day (2), To drink fruit juices as much as you like (3), I don't know what is recommended (4)"	I think it is recommended for children my age (to skip breakfast (1), Eat breakfast when you feel like it (2), eat breakfast on schooldays (3) Eat breakfast every day (4).	I think it is recommended for children my age (to be active once a week (1), to be active some days a eek (2), to be active every day for 30 minutes (3), to be active every day for 1 hour (4), to be active every day for 2 hours (5), to be active every day for 3 to 4 hours (6).	I think it is recommended for children my age (to watch television as often as you like (1), To watch television for more than 2 hours per day (2) To watch television for less than 2 hours per day (3) To watch television for less than 1 hour a day (4) To watch television not more than a few times per week (5) Not to watch television at al (6).

Health beliefs^2^	Belief of the child in that performing the behaviour would make him/her fat	I think that drinking fizzy drinks or fruit squash will make me fat (I fully disagree (-2)- I fully agree (+2))	I think that drinking fruit juices will make me fat (I fully disagree (-2)- I fully agree (+2))	1. I think NOT eating breakfast will make me fat (I fully disagree (-2)- I fully agree (+2)) 2. I think that eating breakfast will make me fat (I fully disagree (-2)- I fully agree (+2))	I think NOT doing physical activities/sports will make me fat (I fully disagree (-2)- I fully agree (+2))	I think watching too much television will make me fat (I fully disagree (-2)- I fully agree (+2))

Preference/liking^1^	Whether the child likes performing the behaviour or likes the taste of food	I like the taste of fizzy drinks or fruit squash (I fully disagree (-2)- I fully agree (+2))			I like doing physical activity/sports (I fully disagree (-2)- I fully agree (+2))	I like watching television (I fully disagree (-2)- I fully agree (+2))

Self-efficacy^1^	The child's perception of how 'easy' or 'difficult' the behaviour is to perform	I find not drinking fizzy drinks or fruit squash... (very difficult (-2) - very easy (+2)).		I find eating breakfast every day... (very difficult (-2) - very easy (+2)).	I find doing physical activity/sports for 1 hour everyday... (very difficult (-2) - very easy (+2)).	I find NOT watching television... (very difficult (-2) - very easy (+2)).

Automaticity^3^	extent to which a behaviour is performed automatically	Drinking fizzy drinks or fruit squash is something I do without even really thinking about it (I fully disagree (-2) - I fully agree (+2)).		Eating breakfast is something I do without even really thinking about it (I fully disagree (-2) - I fully agree (+2)).	Doing physical activity/sports is something I do without even really thinking about it (I fully disagree (-2) - I fully agree (+2)).	Watching television is something I do without even really thinking about it (I fully disagree (-2) - I fully agree (+2)).

**Family environment**						

*Social environment*						

Parental subjective norm^1^	Child's believe that parents consider performing the EBRB as 'good' or 'bad'	If I drink fizzy drinks or fruit squash my parents/caregivers think this is (very bad (-2)- very good (+2))		If I eat breakfast my parents/caregivers think this is (very bad (-2)- very good (+2))	If I do physical activity/sports my parents/caregivers think this is (very bad (-2)- very good (+2))	1. If I watch television my parents/caregivers think this is (very bad (-2)- very good (+2))

Parent modelling^1^	Perceived behaviour performed by parent	How often do your parents/caregivers drink fizzy drinks or fruit squash? (Never (-2)- Always (+2))		How often do your parents/caregivers eat breakfast? (Never (-2)- Always (+2))	How often do your parents/caregivers do physical activity/sports? (Never (-2)- Always (+2))	How often do your parents/caregivers watch television? (Never (-2)- Always (+2))

Performing the ERBR together^1.^	Frequency score of how often the child performs the behaviour together with his/her parent/caregiver			How often do you eat breakfast with your parents/caregivers? (Never (-2) - Every day (+2)).	How often do you take part in physical activity/do sports with your parents/caregivers? (Never (-2) - Every day (+2)).	How often do you watch television with your parents/caregivers? (Never (0)- Every day, more than once a day (14))

Active encouragement/parental support^1^	Perceived encouragement/support from parent in performing the behaviour			My parents/caregivers encourage me to have breakfast. (I fully disagree (-2) - I fully agree (+2)).	My parents/caregivers encourage me to do physical activity/sports. (I fully disagree (-2) - I fully agree (+2)).	

Parental support^1^					My parents/caregivers help me if I need something for my sports. (I fully disagree (-2) - I fully agree (+2)).	

*Political environment*						

parental practices^1^	Family rules with regard to the behaviour perceived by child	1. I am allowed to take fizzy drinks or fruit squash, whenever I want (Never (-2) - Always (+2)). 2. Do your parents/caregivers have rules about how many fizzy drinks or fruit squash you are allowed to drink (No (0) - Yes (1)). 3. If you ask your parents/caregivers to buy a certain brand of fizzy drink or fruit squash, will they do it? (Never (-2)- Always (+2), 4. If I ask my parents/caregivers for a fizzy drink or fruit squash, I get one. (Never (-2) - Always (+2)).	1. I am allowed to take fruit juices, whenever I want (Never (-2) - Always (+2)). 2. Do your parents/caregivers have rules about how many fruit juices you are allowed to drink (No (0) - Yes (1)).	1. Do your parents/caregivers have rules about whether you should eat breakfast? (No (0) - Yes (1)). 2. If you ask your parents/caregivers to buy a certain band of food or drink for breakfast, will she do it? (Never (-2) - Always (+2)).	1. Do your parents/caregivers have rules about whether you should be physically active/do sports? (No (0) - Yes (1)). 2. Do your parents/caregivers allow you to take part in physical activity/do sports? (No (0) - Yes (1)). 3. If you indicate that you like a certain physical activity/sports will your parents/caregivers allow you to do it? (Never (-2) - Always (+2)).	1. Do your parents/caregivers have rules about how many hours per day you are allowed to watch television? (No (0) - Yes (1)). 2. My parents/caregivers allow me to watch television, whenever I want (I fully disagree (-2) - I fully agree (+2)). 3. If I ask my parents/caregivers to watch television, I can do so. (Never (-2)- Always (+2))

*Physical environment*						

Situation specific habit^1^	Situations in which the behaviour is performed	I which situations do you usually drink fizzy drinks or fruit squash (Thick boxes: During the weekend, breakfast, lunch, dinner, at school, while watching TV, as a thirst quencher between meals, during/after sports, when I am with friends, at birthday/parties, I never drink fuzzy drinks or fruit squash)	I which situations do you usually drink fruit juices (Thick boxes: During weekend, breakfast, lunch, dinner, at school, while watching TV, as a thirst quencher between meals, during/after sports, when I am with friends, at birthday/parties, I never drink fruit juices)	I which situations do you usually eat breakfast (Thick boxes: At a set table at home, In bed, While watching TV, on my way to school, at school before the class starts, I never eat breakfast).		How often do you watch television during meals? (Never (-2) - Always (+2)).

Home availability^1^	Availability of food products or equipments	Are there usually fizzy drinks or fruit squash at your home?	Are there usually fruit juices at your home? (Never (-2) - Always (+2)).	Are there usually breakfast products (milk, cereals, bread etc) at your home? (Never (-2) - Always (+2)).	Do you have the following things at home that you can use for physical activities/sports? (Thick boxes: bike, Tennis and/or badminton racket, ball (basketball, volleyball, football etc), sporting shoes, skipping rope, skates, skis, skate board	Do you have a television in your bedroom? (No (0) - Yes (1)).

*Economic environment*						

Pocket money^4^	Spending pocket money on food products	How often do you spend your own money on fizzy drinks or fruit squash? (Never (-2) - Always (+2)).				

Pricing^4^	Consequences of increasing prices of food products	If the price of fizzy drinks and fruit squash were doubles, I would buy less fizzy drinks or fruit squash from my own money (I fully disagree (-2)- I fully agree (+2)).				

**School environment**						

*Social environment*						

Peer subjective norm^1^	Child's believe that peers consider performing the EBRB as 'good' or 'bad'	If I drink fizzy drinks or fruit squash most of my friends think this is (very bad (-2) - very good (+2)).		If I eat breakfast most of my friends think this is (very bad (-2) - very good (+2)).	If I do physical activity/sports most of my friends think this is (very bad (-2) - very good (+2)).	If I watch television most of my friends think this is (very bad (-2)- very good (+2))

Peer modelling ^1^	Perceived behaviour performed by friends	How often do most of your friends drink fizzy drinks or fruit squash? (Never (-2)- Always (+2))		How often do most of your friends eat breakfast? (Never (-2)- Always (+2))	How often do most of your friends do physical activity/sports? (Never (-2)- Always (+2))	How often do most of your friends watch television? (Never (-2)- Always (+2))

^1. ^Item derived from the ProChildren study [18]; ^2 ^Item derived from DOiT study [16]^3 ^Item derived from Habit Strength Index [27], ^4^Items derived from Vogt-Nielsen et al. 2010 [22]

#### Family environmental variables

Table 3 shows the assessed family environmental variables, their description and the items used in the ENERGY questionnaire. In line with the ANGELO framework [14], these family environmental variables can be distinguished into social-cultural environmental factors (i.e. parental subjective norm, parent modelling, and parental support), political environmental factors (i.e. parental rules), physical environmental variables (i.e. situations where the EBRB was performed and home availability), and economic or financial environmental factors (i.e. buying habits of soft drinks or fruit squash from children's pocket money, and the influence of price changes). All family environmental variables were assessed by single items, with the exception of parental rules (assessed by three items) by means of a 5-point Likert scale. The items on the environmental variables were informed by the Pro Children Questionnaire [18] and the ENDORSE study questionnaire [19] and informed by recent reviews of the literature [20,21]. The additional items on the economic environment were developed for this project, informed by insights from a recent study [22].

#### School environmental variables

Social-cultural factors related to the school environment (i.e. peer subjective norm and peer modelling) were assessed by single items using 5-point Likert scales. The items were again informed by the ProChildren [18] and ENDORSE questionnaire [19].

### Parent questionnaire

In the parent questionnaire, self-reported levels of EBRBs as well as personal and family environmental variables were assessed.

#### Energy Balance Related Behaviours

Questions on EBRBs were similar to the child questionnaire (Table 3). Dietary intake, physical activity behaviours, and sedentary behaviour were assessed with frequency questions referring to a general week and were relevant to adult life [16]. In addition, dieting behaviour was assessed using two questions using a five point Likert scale, and a rating scale with scores from 1 (no restrained eating) to 8 (much restrained eating) how much the parent restraint him/herself in eating, informed by restrained eating questions from the Three Factor Eating Questionnaire [17]. Finally the parents were asked about the sleeping habits of their child by means of three questions informed by the HBSC questionnaire [23].

#### Personal variables

Parental status, age, marital status, weight, height, educational level, occupational status, ethnicity child, and date of birth child, were assessed using one question.

#### Family environmental variables

Table 4 shows the family environmental variables included in the parent questionnaire, its description and the questionnaire items. The items were again based on and informed by the Pro Children and ENDORSE parent Questionnaires [18,19]. The family environmental correlates could be distinguished into social environmental correlates (i.e. parental practices including parental beliefs, parental rules and parental modelling, automaticity of the behaviour and the nagging behaviour of the child), physical environmental variables (i.e. home availability), and economic environmental variables (i.e. influence of pricing on child's behaviour; influence of pricing on own behaviour; price consciousness of the child and amount of provided pocket money for food products). The variables were assessed by one or more items using a five-point answering format, except the amount of provided pocket money for food and beverage products that was measured with an 8 point scale, ranging from 0 (I do not give money to my child) to 8 (more than €51 Euro per week (or the approximate equal amount in the local currency)).

**Table 4:** Measurement items of each specific correlates per EBRB of the Parent Questionnaire

	Description	Soft drink	Fruit juice	Breakfast	Dietary behaviour general	Physical activity/Sports	TV viewing
**Family environment**							

*Social environment*							

Parenting practices^1^	Assess upbringing strategies and rules the parent apply with regard to EBRB performed by the child	1. I negotiate with my child how much soft drinks (s)he is allowed to drink 2. If I prohibit my child from drinking soft drinks, I find it difficult to stick to my rule(s), if (s)he starts negotiating 3. I give soft drinks to my child as a reward or to comfort him/her 4. How often do you tell your child that soft drinks are not good for him/her 5. How often do you tell your child that soft drinks can make him/her fat 6. How often do you tell your child that soft drinks are bad for his/her teeth 7. I pay attention to the amount of soft drinks that my child drinks 8. My child is allowed to take soft drinks, whenever (s)he wants 9. If I would like to drink soft drinks, I would restrain myself because of the presence of my child 10. If my child asks for soft drinks, I will give it to him/her	1 I negotiate with my child how much fruit juices (s)he is allowed to drink 2. If I prohibit my child from drinking fruit juices, I find it difficult to stick to my rule(s) if (s)he starts negotiating. 3. I give fruit juices to my child as a reward or to comfort him/her. 4. How often do you tell you child that fruit juices are good for him/her? 5. How often do you tell your child that fruit juices can make him/her fat? 6. How often do you tell your child that fruit juices are bad for his/her teeth? 7. I pay attention to the amount of fruit juices that my child drinks. 8. My child is allowed to take fruit juices whenever (s)he wants. 9. If I would like to drink fruit juices, I would restrain myself because of the presence of my child. 10. If my child asks for fruit juices, I will give it to him/her.	1. I negotiate with my child on how much breakfast products (s)he has to eat and/or drink. 2. If I prohibit my child from skipping breakfast, I find it difficult to stick to my rule(s) if (s)he starts negotiating. 3. I praise my child if (s)he eats breakfast. 4. How often do you tell your child that eating breakfast is good for you 5. I pay attention what kind of products my child is eating for breakfast. 6. My child is allowed to skip breakfast. 7. I encourage my child to have breakfast.	What do you consider to be the three most important characteristics of your child's meal during school hours	1. I negotiate with my child on how much physical activity/sports (s)he has to do 2. If I try to prohibit my child from skipping a physical activity/sport session, I find it difficult to stick to my rule(s) if (s)he starts negotiating 3. I praise my child if (s)he takes part in physical activity/sports. 4. I punish my child by not allowing him/her to take part in his/her physical activity sessions/sports 5. How often do you tell your child that physical activity/sports are good for him/her? 6. I pay attention that my child does enough physical activity/sports 7. My child is allowed to skip physical activity/sport sessions whenever (s)he wants 8. I set a time limit on how much time of physical activity/sports my child can do in order to devote more time to his/her homework or other important things. 9. I do not allow my child to take part in physical activity/sports in his/her free time so that (s)he can concentrate on his/her studies. 10. I pay for my child to take part in physical activity/sports. 11. I bring my child to physical activity/sport sessions. 12. I encourage my child to take part in physical activity/sports	1. I negotiate with my child how much TV/video/DVD (s)he is allowed to watch. 2. If I prohibit my child from watching TV/video/DVD, I find it difficult to stick to my rule(s) if (s)he starts negotiating. 3. I allow my child to watch TV/video/DVD as a reward or to comfort him/her. 4. How often do you tell your child that watching TV/video/DVD is not good for him/her? 5. How often do you tell your child that watching TV/video/DVD can make him/her fat? 6. How often do you tell your child that watching TV/video/DVD is bad for his/her eye sight? 7. I pay attention to the amount of time my child watches TV/video/DVD. 8. My child is allowed to watch TV, whenever (s)he wants) 9. If I would like to watch TV/video/DVD, I would restrain myself because of the presence of my child. 10. If my child asks if (s)he is allowed to watch TV/video/DVD, I will allow it.

Automaticity^2^	Assess the extent to which the behaviour is performed automatically	Drinking soft drinks is something that I do without even really thinking about it	Drinking fruit juices is something I do without even really thinking about	Eating breakfast is something I do without even really thinking about		Physical activities/sport activities is something that I do without really thinking about	Watching television is something I do without even really thinking about

TV watching while having meal^1^					In general, how often do you watch television during the following meals?		

Performing the EBRB together^1^	Frequency score of how often parent performs the EBRB together with the child	How often do you or your spouse drink soft drinks together with your child?	How often do you and/or your spouse/partner drink fruit juices together with your child?	How often do you and/or your spouse/partner have breakfast together with your child?	1. How often do you and/or your spouse/partner have lunch together with your child? 2. How often do you and/or your spouse/partner have dinner together with your child?	How often do you and/or your spouse/partner participate in physical activity/sports together with your child (e.g. play games outside, ride bikes, walk, or play sports together)?	How often do you (one parent/spouse/partner or both) watch television together with your child?

Nagging behaviour of child^1^	Assess how the child behaves despite prohibition of the behaviour by the parent	If I prohibit my child from drinking soft drinks (s)he tries to drink it anyway.	If I prohibit my child from drinking fruit juices (s)he tries to drink it anyway.	If I prohibit my child from skipping breakfast, (s)he tries to skip it anyway.		If I try to prohibit my child from not taking part in a physical activity/sport session, (s)he will try to skip it anyway.	If I prohibit my child from watching TV/video/DVD, (s)he tries to watch anyway.

*Physical environment*							

Home availability^1^	Assess whether certain food products or equipment are available for the child.	There are soft drinks available at home for my child.	There are fruit juices available at home for my child.	There are breakfast products (e.g. milk, cereal, bread) available at home for my child.			TV/video/DVD is available in my child's room.

*Economic environment*							

Influence pricing on child's behaviour^3^	Parental believe of whether child changes buying habits if prices change	If the price of soft drinks were double, my child would drink less soft drinks.					

Influence pricing own behaviour^3^	Influence of prices on performing own behaviour.				I don't give my child some foods because they cost too much.	I let my child participate in activity/sports less than I would like, because it is too expensive.	

Price consciousness child^3^	Assess whether the parent would consider his/her child as being price conscious regarding food products				I would consider my child as being price conscious regarding food, snacks and beverages.		

Pocket money for food^3^	How much pocket money the parent weekly gave to the child to buy food and drinks				On average how much money do you give to your child to buy food and drinks per week? Please do not include money you save or spend on clothes for your child		

^1. ^Item derived from the ProChildren study [18]; ^2 ^Item derived from Habit Strength Index [27], ^3^Items derived from Vogt-Nielsen et al. 2010 [22]

### School management questionnaire

The school management questionnaire was developed to describe the variation in food and physical activity related facilities and items within the school environment and to get insight into school policies and was informed by the Pro Children staff questionnaire [18], ENDORSE study [19], ANGELO framework [14] and the IDEA study [24]. The questionnaire was completed by the school manager. Table 5 shows the items and answer format of the school management questionnaire. In addition to general characteristics of the school, the questionnaire addressed the *physical environment *(i.e. Opportunities to eat/drink and be physically active, such as offering/practicing any additional opportunities to be physically active; perceived safety to walk or bike to school; scheduled times to eat main meals or a "snack"); the *social environment *related to healthy eating and physical activity (i.e. role modelling teachers; social support teachers; social support parents); and the *political environment *concerning regulations and practices pertaining to food/drinks, physical activity, and health. Lastly, *economic environmental *variables related to eating/drinking and physical activity were assessed (i.e. which economic factors/sponsorships have been used, whether the school participated in national or regional campaigns using rewards for the pupils).

**Table 5:** School management questionnaire

School environment	Physical activity behaviour	Dietary behaviour	Physical activity and Dietary Behaviour
*Physical environment*			

Opportunities to eat/drink and be physically active	1. How do the majority of the 5^th^-6^th ^graders in your school get to/from school? (1 = By motorised transportation; 2 = By walking/biking; 3 = About 50/50 of each of the above 2. Is it generally regarded as safe to walk or bike to your school? (0 = no; 1 = yes) 3. Are there scheduled times for recess between lessons for 5-6^th ^graders? (0 = no; 1 = yes, please fill in the number of the recesses, start time and end time) 4. How many lessons of required physical education are scheduled per week for the 5^th^-6^th ^graders in your school? (please fill in..) 5. What is the scheduled time per lesson of physical education for the 5^th ^- 6^th ^graders? (1 = 30 minutes or less; 2 = 30-45 minutes; 3 = 46-60 minutes; 4 = 61-75 minutes; 5 = 76-90 minutes; 6 = More than 90 minutes) 6. Does your school regularly (i.e. weekly) offer or practice any of the following additional opportunities for 5^th ^- 6^th ^graders to be physically active? a) Brief teacher organized physical activities in lessons other than physical education (0 = no; 1 = yes) b) Organised sport activities before or after the school day (i.e. school sports team, after-school activities) (0 = no; 1 = yes)	1. Are there scheduled times to eat main meals or a "snack" for 5-6^th ^graders? (0 = no; 1 = yes, please fill in the name of the eating occasion, start time and end time) 2. From where does the majority of 5-6^th ^graders get the food/drinks that they consume during the school day? *(Please check all that apply) *(0 = no; 1 = yes for meals; 2 = yes for snacks) a) Home (i.e. bagged lunch) b) Shops etc around the school (i.e. bought on the way to school or in recess during the school day) c) Subscription programs offered through school (i.e. milk, fruit, sandwiches) d) School canteen e) School shop/kiosk f) Vending machines on the school premises 3. How are the meals/snacks obtained at school paid for? *(Please check all that apply) *(0 = NA; 1 = meals; 2 = snacks) a) Parents prepay - in full b) Parents prepay - but partly subsidized (by school/government) c) Parent pay afterwards (by invoice) d) Pupil/parent pay at point of purchase - in full e) Pupil/parent pay at point of purchase - but partly subsidized (by school/government) f) School pay - in full (through own budget) g) School pay - but subsidized by government (local/national) h) Local/national government pay - in full i) Private companies pay - in full j) Private companies pay - in part k) Offered for free/subsidized price to pupils from low-income families	

*Social environment*			

Social factors related to healthy eating and physical activity	1. Rate to what extent teachers/other adults at your school act as role models by being physically active (0 = small; 4 = very large) 2. Rate to what extent play or physical activity during recess is promoted at your school. (0 = small; 4 = very large) 3. Rate to what extent the majority of parents at your school support physical activity to/from and in school. (0 = small; 4 = very large)	1. Rate to what extent teachers/other adults at your school act as role models by eating healthy foods/drinks. (0 = small; 4 = very large) 2. Rate to what extent healthy foods/drinks are promoted at your school's social/sporting events (0 = small; 4 = very large) 3. Rate to what the extent the majority of parents at your school support healthy eating in school. (0 = small; 4 = very large)	1. Rate to what extent promoting healthy eating and/or physical activity is regarded as important at your school. (0 = small; 4 = very large) 2. Rate to what extent the school health services promote healthy eating and/or physical activity at your school (0 = small; 4 = very large) 3. Rate to what extent you personally regard promoting healthy eating and/or physical activity as important for schools. (0 = small; 4 = very large)

*Political environment*			

Available organized services and practices related to eating/drinking and physical activity	1. Please, indicate which of the following physical activity practices you consider part of your school's (daily) routine. (Write 0 = no; 1 = partly/sometimes; 2 = yes; 3 = NA) a) Encourage biking/walking to school b) Provide bike racks or a designated area for bike parking c) Work to ensure traffic safety at start/end of the school day d) Require pupils to be outside during recess e) Allow pupils to use own equipment (balls, ropes etc) during recess f) Provide access to sports equipment (balls, ropes etc) during recess g) Provide adult/teacher supervision of school grounds during recess h) Encourage pupils to play or be physically active during recess i) Teach about physical activity and health as part of the curriculum j) Teach skills to practice specific sports as part of the curriculum k) Require physical education teachers to be certified/specialists in physical education l) Require pupils to be dressed for physical activity in physical education lessons m) Provide clean and separate changing rooms with showers n) Require pupils to shower after physical education lessons o) Require a note from parents for single absences from physical education p) Require a note from a doctor for long term absences from physical education q) Provide alternative physical activities in physical education for pupils with special needs r) Provide parents with feedback on their child's development in physical education s) Give physical education homework Include physical activity in lessons other than physical education t) Allow general use of school grounds (incl. outdoor facilities) before/after the school day u) Encourage teachers and other staff to act as role models for physical activity v) Address the importance of everyday physical activity at parent meetings w) Address the importance of everyday physical activity in written information to parents 2) In which forums or with whom have physical activity practices been discussed at your school in the past 2 years? (0 = No; 1 = Yes, to some extent; 2 = Yes to a great extent) a) Teacher/staff meetings b) Parent meetings c) With pupils d) With school nurse/doctor e) other	1. Please, indicate which of the following eating/drinking practices you consider part of your school's (daily) routine. (Write 0 = no; 1 = partly/sometimes; 2 = yes; 3 = NA) a) Provide easy access to fresh drinking water at all times b) Apply nutritional guidelines for what foods/drinks may be offered in the canteen c) Apply nutritional guidelines for what foods/drinks may be sold in the school shop/kiosk d) Apply nutritional guidelines for what foods/drinks may be sold in vending machines e) Prohibit that pupils bring unhealthy foods/drinks to school on a regular school day f) Allow pupils to leave school during any or all of the recesses g) Provide adequate eating facilities (i.e. clean, access to water to wash hands, waste bins) h) Provide adult supervision during scheduled snacks/meal times i) Encourage teachers and other staff to act as role models for healthy eating j) Encourage pupils to eat and drink healthy foods and drinks k) Allow pupils to eat whenever they want l) Limit access to canteen to snack/meal times only m) Limit access to school shop/kiosk to snack/meal times only n) Limit access to vending machines to snack/meal times only o) Provide healthy food/drink options at social/sporting events p) Provide healthy food/drink options at parent meetings q) Provide healthy food/drink options at teacher trainings and other staff meetings r) Allow marketing (i.e. posters, material, events) of unhealthy foods/drinks at/through school s) Prohibit sale of unhealthy foods as fund raising during school days t) Prohibit use of unhealthy food/drinks as rewards by teachers and other staff u) Teach about food, nutrition and health as part of the curriculum v) Teach practical cooking skills as part of the curriculum w) Address the importance of everyday healthy eating at parent meetings x) Address the importance of everyday healthy eating in written information to parents 2) In which forums or with whom have eating/drinking practices been discussed at your school in the past 2 years? (0 = No; 1 = Yes, to some extent; 2 = Yes to a great extent) a) Teacher/staff meetings b) Parent meetings c) With pupils d) With school nurse/doctor e) other	1. Are any of the practices discussed stated in written documents? *(Please check all that apply) *(0 = No; 1 = Yes, in a school health policy; 2 = Yes, in a school food policy; 3 = yes in a school physical activity policy; 4 = other) 2. For how long has the school had these policy documents? (1 = less than a year; 2 = 1-2 years; 3 = 3-4 years; 4 = 5 years or more) a) health b) food c) physical activity

*Economic environment*			

Economic factors related to eating/drinking and physical activity	1. Please, indicate which of the following economic factors/sponsorships have been used in your school in the past 2 years. (Write 0 = No; 1 = partly/sometimes; 2 = yes; 3 = not applicable) a) Obtained sponsorship to buy sports equipment for recess b) Obtained sponsorship to maintain/renovate the school ground (incl. play areas) c) Obtained sponsorship to maintain/renovate outdoor or indoor sport facilities d) Obtained sponsorship to offer extra physical activity during school hours e) Obtain sponsorship to offer after school sports programs 2. Has the school participated in national or regional campaigns with the aim of promoting physical activity in the past 2 years, involving any form of (chance of) reward? 0 = No, the school has not participated in such campaigns during the last 2 years; 1 = Yes, a campaign involving a reward for all pupils on the school (e.g. school party, playground facilities, concert, money prize etc) 2 = Yes, a campaign involving a reward for a group of pupils on the school (e.g. party, excursion, money prize etc) 3 = Yes, a campaign involving a reward for single pupils (e.g. money prize, book, CD's, etc) 4 = Yes, a campaign involving a reward for the school 5 = The school has participated in campaign(s) without such reward schemes 6 = Don't know	1. Please, indicate which of the following economic factors/sponsorships have been used in your school in the past 2 years. (Write 0 = No; 1 = partly/sometimes; 2 = yes; 3 = not applicable) a) Free/subsidized milk subscription program for all b) Free/subsidized fruit/vegetable subscription program for all c) Free/subsidized lunch for all d) Free/subsidized snacks for all e) f) Reduced prices on healthy options in canteen g) Reduced prices on healthy options in shop/kiosk h) Reduced prices on healthy options in vending machines i) Offered package deals on healthy options (i.e. healthy food with healthy drink) j) Used coupon systems for healthy options (i.e. buy 5 - get the 6^th ^for free) k) Increased prices on unhealthy options in canteen/shop/kiosk/vending machines l) Obtained sponsorship for healthy foods/drinks to social/sporting events m) Obtained sponsorship for unhealthy foods/drinks to social/sporting events n) Obtained sponsorship for healthy foods/drinks on regular school days o) Obtained sponsorship for unhealthy foods/drinks on regular school days 2. Has the school participated in national or regional campaigns with the aim of promoting healthy diets in the past 2 years, and involving any form of (chance of) reward? 0 = No, the school has not participated in such campaigns during the last 2 years 1 = Yes, a campaign involving a reward for all pupils on the school (e.g. school party, playground facilities, concert, money prize etc) 2 = Yes, a campaign involving a reward for a group of pupils on the school (e.g. party, excursion, money prize etc) 3 = Yes, a campaign involving a reward for single pupils (e.g. money prize, book, CD's, etc) 4 = Yes, a campaign involving a reward for the school 5 = The school has participated in campaign(s) without such reward schemes 6 = Don't know	1. Please, indicate which of the following economic factors/sponsorships have been used in your school in the past 2 years. (Write 0 = No; 1 = partly/sometimes; 2 = yes; 3 = not applicable) a) Applied for funding that required the implementation of certain healthy eating and/or physical activity practices

### Audit instrument for school environment

Partly based on the audit instrument used in the ENDORSE study [19], the ENERGY-team developed an audit instrument to assess the availability, accessibility, and commercial advertising of food and drinks and also identify the opportunities to stimulate physical activity within the school environment. At each school, two research assistants completed the audit instrument independently.

Table 6 shows the items and answer formats of the audit instrument. The audit instrument consisted of nine parts: (1) food/drink available in the cafeteria; (2) food/drink available in vending machines; (3) subscription programs; (4) commercial advertising; (5) bicycle parking; (6) equipment for recess; (7) indoor physical activity facilities; (8) outdoor physical activity facilities; and (9) other information of outdoor areas. The audit instrument had a 'tick box' answering format and included observation of objective characteristics. When subjective characteristics such as 'state of maintenance of the school yard', or 'condition of the bicycling parking' were reported, photographs were taken.

**Table 6:** Audit instrument for school environment

Interview to the canteen/school shop administrator	
*Canteen/school shop*	1. How many days per week is the canteen/school shop operated? 2. What are its hours of operation? 3. Does the canteen/shop sell/offer any meals? (0 = No; 1 = Yes, breakfast; 2 = Yes, lunch; 3 = Yes, other) 4. What are the main product groups sold in the canteen? (1 = Sandwiches; 2 = Hot meals; 3 = Drinks; 4 = Salt/sweet snacks/cakes; 5 = Other) 5. Who owns/run the canteen/school shop? (1 = The school; 2 = Parents; 3 = Private; 4 = Other) 6. Who decides what is offered in the canteen/school shop? (1 = The school management; 2 = The canteen/shop manager' 3 = Pupils; 4 = Other) 7. What are the three most sold food products in the canteen/school shop? 8. What are the three most sold drink items in the canteen/school shop? 9. Is it possible for students to stay in the canteen/school shop before/after breaks? (0 = No; 1 = Yes) 10. Is there fresh drinking water offered in the canteen? (0 = No; 1 = Yes, for free; 2 = Yes, for sale) 11. Does the school make profit on canteen/school shop from products' sales or as rent to the company/person who is running the canteen/school shop? (0 = No; 1 = Yes; 2 = NA; If Yes, how much?) 12. How does the school use that profit?

*Vending machines*	1. What types of vending machine(s) are available? (type; N° of machines; location) 2. What are the three most sold food products in the vending machine(s)? 3. What are the three most sold drink items in the vending machine(s)? 4. How often are the vending machines refilled? 5. Are there any restrictions on hours of operation/accessibility of vending machines? (0 = No; 1 = Yes; if Yes please explain) 6. Are any vending machines owned and operated by the school? (0 = No; 1 = Yes) 7. Does the school make profit on vending machine(s) from product sales or as rent to the company/person who is running the vending machine(s)? (0 = No; 1 = Yes; 2 = n/a; If Yes, how much? 8. How does school use that profit?

*Subscription programs*	1. Name of program (i.e. School fruit scheme) 2. Offered products 3. Offered by whom? (1 = Government; 2 = Large/national food company; 3 = Small/local food company; 4 = Other) 4. Paid by whom? (1 = By governments; 2 = By the school; 3 = By parents; 4 = Other) 5. How administrated in the school? (1 = By staff paid from school; 2 = Parents; 3 = Other)

**Food/drink registration**	

*Canteen/school shop-*	Products Observation Form (product; gr/portion; Kcal/portion; No of products; Comments) 1. Bread‐products (e.g. tuna sandwich, egg sandwich, cheese pie) 2. Hot meals 3. Drinks (e.g. coca cola, sprite, fanta, fruit juice, water, milk, ice tea, tea, coffee) 4. Snacks/cakes/candies (e.g. chips, mars, gingerbread) 5. Fruits and Vegetables 6. Other (e.g. salads) 7. Attach a photograph(s) of the canteen/school shop, so that is well visible what products are placed on and behind the counter. 8. Attach a photograph(s) of the wider area of the school shop/canteen, so that the facilities/dining area are well visible.

*Vending machines*	1. Indicate where the vending machine is located (e.g. canteen/school shop, entrance, hallway). 2. Type of machine (e.g. beverage, snack, mixed) 3. Is the machine currently on and available to students or is it turned off? (0 = Off; 1 = On) Products Observation Form (product; gr/portion; Kcal/portion; No of products; Comments) 1. Bread‐products (e.g. tuna sandwich, egg sandwich, cheese pie) 2. Drinks (e.g. coca cola, sprite, fanta, fruit juice, water, milk, ice tea, tea, coffee) 3. Snacks/cakes/candies (e.g. chips, mars, gingerbread) 4. Other 5. Attach a photograph(s) of the vending machine(s), so that the products available are well visible. 6. Attach overview photograph(s) of the location(s) of the vending machine(s), in which the biggest part of the machine is visible (Location of the machines in the canteen/school shop needs to be visible).

**Observation forms**	

*Food and drink advertising*	1. Are there any food or beverage advertisements in the following locations (tick applicable options)? (1 = In the canteen school shop; 2 = On vending machines; 3 = In school buildings; 4 = On school grounds, including the outside of school buildings, on playing fields, or other areas of school; 5 = Other (please clarify) 2. Please indicate the types of food/beverages advertised: a) Food (0 = No food advertisements; 1 = Fruits and vegetables; 2 = Bread products (e.g. sandwiches, pies); 3 = Snacks, cakes, candies; 4 = Other (please clarify) b) Beverages (0 = No beverage advertisements; 1 = 100% fruit juice; 2 = Sweetened drinks (incl. fizzy drinks); 3 = Diet drinks; 4 = Milk; 5 = Water; 6 = Other (please clarify)

*Bicycle parking area (designated by the school)*	1. Is the bicycle parking covered (i.e. by a roof/shed)? (0 = No; 1 = Yes; 2 = Partly) 2. Is the bicycle parking visible from the public road? (0 = No; 1 = Yes) 3. Is there supervision at the bicycle parking? (0 = No; 1 = Yes, if Yes: 1 = Doorkeeper; 2 = Camera; 3 = Reception; 4 = Other 4. Is the bicycle parking surrounded by a fence? (0 = No; 1 = Yes) 5. Are there bicycle racks? (0 = No; 1 = Yes) 6. How many bicycles can be placed in the racks? (count 1 bicycle rack, multiply number of racks, count up all the racks with different sizes) 7. Is the bicycle parking full of bicycles? Number of bikes: Full > 75%; Mean 25 - 75%; Empty < 25% 8. Attach an overview photograph of the bicycle parking

*Physical activity equipment (loose) for recess*	Available equipment (0 = No; 1 = Yes; N°; Condition (e.g. new, clean, damaged, poor quality) 1. Footballs 2. Basketballs 3. Volleyballs 4. Other balls: 5. Rackets (i.e. tennis, badminton, squash) 6. Bandy/hockey sticks 7. Bats (i.e. baseball) 8. Skipping ropes 9. Jump bands (elastics) 10. Frisbees 11. Other (i.e. seasonal equipment) 12. Other____________ 13. If possible, attach an overview photograph of the room/box for keeping the equipment 6b. 14. If possible, attach an overview photograph of the type of equipment

*Indoor sport facilities*	Available facilities (0 = No; 1 = Yes; N° of facilities; Condition (e.g. new, clean, damaged, poor quality) 1. Gym 2. Swimming pool 3. Weight training facilities. 4. Gender specific changing rooms 5. Shower facilities 6. Other 7. If possible, attach an overview photograph of the indoor gym 8. If possible, attach an overview photograph of the equipment for the indoor gym

*Outdoor sport facilities*	Available facilities (0 = No; 1 = Yes; N° of facilities; Condition (e.g. new, clean, damaged, poor quality) 1. Football field 2. Basketball field 3. Volleyball/badminton/tennis fields with net 4. Other marked fields for ballgames: __________ 5. Paint/marks for other games 6. Play area with swings, slides etc (for young children) 7. Table tennis 8. Track and field (running, jumping, throwing) 9. Obstacle course/Jungle path 10. Climbing (walls, trees) 11. Skateboard area 12. Other:_____________ 13. Attach an overview photograph of the outdoor sport facilities 14. Attach an overview photograph of the outdoor sport facilities

*Other information about the school outdoor area*	1. What kinds of surfaces are present? (1 = Asphalt/paved; 2 = Grass; 3 = Gravel; 4 = Sand; 5 = Rocks; 6 = Nature/woods; 7 = Other) 2. How is the topography? (1 = Flat; 2 = Hilly; 3 = Combination of both; 4; Other) 3. How is the school's outdoor area separated from the neighbourhood? (1 = Fence/Hedge; 2 = No clear boundaries; 3 = Other) 4. Are there other things present? (1 = Plants/trees; 2 = Benches (and tables); 3 = Drinking fountains; 4 = Other) 5. General appearance of the outdoor area a. Clean (0 = No; 1 = Yes) b. Safe (0 = No; 1 = Yes) c. Inviting (0 = No; 1 = Yes) 6. If relevant, attach a photograph of a physical activity promoting characteristic of this school 7. If relevant, attach a photograph of barriers to physical activity characteristic of this school

### Anthropometrics

In each country, at least two trained research assistants measured body height, weight, and waist circumference according to a standardized protocol. The children were measured without shoes and were allowed to wear light clothing, such as a t-shirt and shorts/short pants. Body height was measured with a Seca Leicester Portable stadiometer with an accuracy of 0.1 cm. Weight was measured with a calibrated electronic scale SECA 861 with an accuracy of 0.1 kg. The waist circumference was measured with the circumference measuring band SECA 201 to the nearest 0.1 cm.

Two readings of each measurement (weight, height and waist) were obtained to assure accuracy. If the two readings differed more than 1% then a third measurement was taken. All three measurements were recorded and the outlier was excluded during the data cleaning process. The researcher was not allowed to provide the child with their weight and waist circumference measurement results.

#### Training of research assistants

Since the precision of the anthropometrical measurements play an important role, the ENERGY-project team aimed to minimise the measurement error within and across countries. Therefore at one of the ENERGY meetings, at least one researcher per participating country was trained for the anthropometry measures. The intra-rater reliability rates ranged from 0.999 to 1.00 for weight and height measurements and 0.942 to 0.999 for waist measurements. The inter-rater reliability was 1.00 for weight and height measurements and 0.990 for waist circumference measurements.

### Additional measurement instruments in a selection of countries

#### Blood samples

Blood samples were taken in order to assess the following blood parameters: fasting plasma glucose, C-peptide, total cholesterol, high density lipoprotein cholesterol, and triglycerides. Capillary blood was collected with a finger prick using a well-validated collection kit developed for ambulatory purposes (Demecal, Haarlem, The Netherlands) [25]. These procedures are similar to the procedures of the Amsterdam Birth Cohort Development (ABCD) study [26].

#### Accelerometers

Triaxis (GT3X) and Uniaxis (GT1M) Actigraph models were used to collect data on physical activity and sedentary behaviour. Trained research assistants taught the children how to correctly wear the accelerometers on the right side of the waist. Children were asked to wear the accelerometer for six consecutive days (including at least one weekend day) except at night while sleeping or during any water activities (e.g., bathing, swimming). Time intervals (epochs) were set to 15 seconds. Teachers were informed about the procedures and asked to remind the children to wear the devices every day. Additionally, the children and parents received a brochure about accelerometer use and a diary. We asked children to write down the time of getting up in the morning and going to bed for sleeping; the time and reason why the device was removed for 5 minutes or more for any activity such as swimming, or showering; and whether the wearing day was a school day, half-school day or non-school day.

## Data Handling and Transformation

The child and parent questionnaires from all countries were shipped to the coordinating centre in the Netherlands and were there scanned and the data were transferred to SPSS files. The data of the audit instrument, school management questionnaire, and anthropometry data was entered manually into an Excel file by the researchers in each country, and converted into SPSS files, sent to the coordinating centre and there merged and checked by a data manager.

The following quality checks on data entry were performed. Of the anthropometry measurements, 10% were entered twice in an Excel sheet by two separate researchers and cross-checked. Of the audit instrument and the school management questionnaire, 20% of the data were entered in the Excel files by two separate researchers, followed by another cross-check. The raw data of the child and parent questionnaires were provided with variable labels, value labels, and missing value labels (see additional file 2 for the codebooks) and cleaned on double records, non-existing participants, missing values on compulsory questions, out-of-range values, and inconsistencies. The final cleaned data files from all measurement sources and countries were combined based on the child and/or school ID to form the final raw data file. The cleaned data file was then submitted to recoding and transformations to create and calculate variables ready for analyses.

## Data Analyses

Descriptive analyses will be performed first. Subsequently bivariate and multivariate models will be tested using a range of regression and other analyses to test correlates and mediation models of overweight, obesity, metabolic outcomes and EBRBs. Since the data of the children are clustered within classes and schools, which are clustered within countries, data will be analysed using multilevel analyses when appropriate. The analyses will be conducted on the international sample as well as country or region specific analyses.

## Discussion

The ENERGY survey is a comprehensive cross-sectional study collecting data on anthropometrics, physical activity behaviours and biomarkers as well as assessing a range of EBRBs and their potential determinants at the personal, school environment and family environment levels, among 10-12 year old children in seven European countries. This study will result in a unique data set, enabling cross country comparisons in overweight, obesity, risk behaviours for these conditions as well as the correlates of engagement in these risk behaviours.

An important strength of the ENERGY-survey study concerns the number of participating countries from different regions in Europe including countries that lack data on EBRB and potential correlates among schoolchildren. In addition, the data set allows unique comparisons of EBRBs and their correlates between a diversity of countries and regions. Another strength of the study is the range of potential relevant EBRBs included as well as the broad range of potential personal and environmental correlates of these behaviours. The objective measures of height, weight and waist circumference from all participating children, as well as accelerometer measures of physical activity and sedentary behaviour and blood samples from subgroups of respondents, further enriches the data set. All these measurements were obtained according to standard methodology and protocols in all participating countries.

The ENERGY-survey also has several potential weaknesses. Although we have obtained several measurements objectively (e.g. height, weight, waist circumference), other measures are self-reported and thus liable to social desirability and recall bias. In addition, to lower the burden for children and parents, the number of items that could be included in the questionnaire was firmly restricted. Therefore, we chose to assess the correlates with only a few or single-item measures, possibly reducing the reliability and increasing measurement error. However, previous analyses showed that the correlates measured with 1-item questions showed significant associations with EBRBs [18]. A further weakness of the ENERGY survey is its cross-sectional design. This means that we will be able to explore *correlates *of EBRB and obesity, but not *true determinants*. Finally, there were some variations in sampling between countries that may reduce the validity of cross country comparisons. Nevertheless, we believe that the ENERGY-project with its cross-European approach is a unique endeavour to study overweight prevalence, associated EBRBs, and their potential personal, family environmental, school environmental and economic environmental correlates in different European countries.

## Competing interests

The authors declare that they have no competing interests.

## Authors' contributions

STV and JB developed the concept and design of the ENERGY study. MVS, STV and JB drafted the manuscript. All other co-authors have been involved in the development, coordination and/or implementation of the ENERGY survey. All authors have read and approved the manuscript.

## Pre-publication history

The pre-publication history for this paper can be accessed here:

http://www.biomedcentral.com/1471-2458/11/65/prepub

## Supplementary Material

Additional file 1 **Fieldwork protocol of the ENERGY survey**. Click here for file

Additional file 2 **codebook of ENERGY instruments**. Click here for file
