# Supplementary material for: EuropeaN Energy balance Research to prevent excessive weight Gain among Youth (ENERGY) project: Design and methodology of the ENERGY cross-sectional survey
Source: BMC Public Health. 2011 Jan 31;11:65. doi: 10.1186/1471-2458-11-65 (PMC3044658; doi:10.1186/1471-2458-11-65)
Supplement: Additional file 1 — Fieldwork protocol of the ENERGY survey. [file 1471-2458-11-65-S1.PDF]

EuropeaN Energy balance Research to prevent excessive weight Gain among Youth

The ENERGY Cross-sectional survey

Fieldwork Protocol

January 2010

Table of Contents

A. Concept **4**

B. Ethical approval **5**

C. Sampling procedures **6**

School recruitment 6

Response Rates 6-7

Timeframe 7

D. Preparation of documents and training **7**

Letter to headmaster 7-8

Letter to parents 8

Translation of child and parent questionnaires into local languages 8

Lay out. 9

Audit instrument and school staff questionnaire 9

Pre-testing in UK, Greece and the Netherlands 9

Pre-testing in all countries 9

Anthropometry training 10

E. Field procedures **10**

Send out letters - timeframe 10

Meeting with the headmaster and instruction of teachers – timeframe and informed consent procedure 10

Questionnaires 11-12

Matching data 12

Delivery of the questionnaires 12-13

Responses to questions from pupils 13

Collection of envelopes by the teachers 13

Anthropometry measurements 13-14

Absentees 14

School management questionnaires 14

Audit instrument 14

Quality check 14

F. Accelerometer study **15**

G. Data handling and entry **16**

Data delivery 16

Questionnaires......16

Excel files......16

Data entry......16

Identification......17

Missing information......17

Back up procedures......17

Data cleaning......17

Data storage and safety......17

H. Feedback to schools 18

Appendices

Appendix A **Letter to headmaster**

Appendix B **Letter to parent**

Appendix C **Child Questionnaire - Pre test protocol and report**

Appendix D **Parent Questionnaire- Pre test protocol and report**

Appendix E **Audit Instrument - Pre test protocol and report**

Appendix F **School management questionnaire - Pre test protocol and report**

Appendix G **Children questionnaire protocol**

Appendix H **Anthropometry datasheet**

Appendix I **Anthropometry protocol**

Appendix J **Accelerometer protocol**

A. Concept

Research has shown that the number of children affected by overweight and obesity is now rising at more than 400,000 a year and already affects almost one in four children across the entire Europe, including accession countries in 2002.

Overweight and obesity are the result of health behaviours related to nutrition and physical activity causing a positive energy balance; when energy intake through nutrition exceeds energy expenditure, a positive energy balance will result to weight gain. It is less clear which specific diet and physical activity behaviours contribute to the risk of excess weight gain in different populations.

ENERGY is a unique project, which for the first time will systematically assess modifiable determinants of energy-balance health behaviours and address the promotion of these behaviour in a European-wide setting.

The main objective of ENERGY is to build a school based, family involved intervention scheme for the promotion of health behaviours that contribute to obesity prevention in children aged 10-12 years old across Europe. In order to achieve that aim, ENERGY will conduct a cross-European school-based survey. At a second stage the results of the data analyses of this cross European survey will support the decision making process regarding the most important and prevalent energy balance behaviours and their determinants that need to be addressed in the intervention.

The present protocol describes the background and practical procedures for the cross-sectional survey that will be carried out in March, April and May 2010 by partners in eight different European countries; Belgium, Greece, Hungary, the Netherlands, Norway, Slovenia, Spain and Switzerland.

The ENERGY project is supported by the European Commission and is funded by the Seventh Framework Programme (CORDIS FP7) of the European Commission, HEALTH.

B. Ethical approval

All participating countries will collect data among children and their parents. The parents will be fully informed about the contents and the aims of the study and will be asked for written consent for the participation of their child and themselves in the ENERGY project.

The pupils will complete a self administered questionnaire, which aims to identify their dietary and physical activity habits. Additionally, measurements on the schoolchildrens' height, weight and waist circumference will be performed. The parents will also complete a self administered questionnaire related to their child's nutritional and physical activity behaviours.

This project will in all aspects adhere to the Helsinki Declaration and the conventions of the Council of Europe on human rights and biomedicine. Prior to initiating the project, ethical clearance will have to be obtained from the relevant ethical committees and ministries in all participating countries. Furthermore, research permission, depending on considerations on local and national level, might have to be obtained from local school authorities (local school boards and/or headmasters).

C. Sampling procedures

School recruitment

Random school selection in each country with the exception of Spain (for Spain, the schools will be selected in the region of Aragon), Switzerland (only German speaking regions) and Belgium (for Belgium, the schools will be selected in Flanders, the Dutch-speaking part of Belgium) will be employed. Schools will constitute the sampling unit, and from each country samples of at least 20 schools and 1,000 eligible children will be included. This student sample size was seen as sufficient to allow for the planned within-country comparisons (gender, socio-economic status and urban-rural differences) and taking the cluster sample into account. Due to the differences observed in the participating countries in terms of distribution of population within the different regions, the sampling of schools will be random, multistage and stratified by the degree of urbanization of the different provinces. More specifically, the percent of population living in municipalities having more than 20,000 inhabitants for each province will be calculated (Step 1 & 2). The provinces will then be ranked according to these percentages, tertiles will be formed based on urbanization degree (step 3 & 4) and one province will be randomly selected from each tertile (step 5). From each of the three selected provinces one municipality having more than 20.000 inhabitants will be randomly selected (step 6). For the 3 selected municipalities, a list of all schools (step7) will be created and sent to ResCon. ResCon will then randomly select the schools (step 8). Based on the randomly selection of schools the countries can start recruiting the schools following the provided rank order.

Response rates

In order to get at least 1000 children and parents it is necessary to over-sample. Considering a dropout rate of 10 % it is recommended to acquire approximately 1100 school children and parents. Sampling should be done by schools and not by individuals.

To increase the response rate, it is important to take a number of factors into account in the recruitment procedure and in all contact with the schools. Briefly, these factors are;

- *School benefits;* It is important to explain the benefits that the school will get out of this procedure. Such benefits for the school can be to use the survey as a part of the education, (how to complete a questionnaire or as a part of health education). Important to emphasize that all school results will remain confidential.

- All *telephone contacts* with headmasters and school staff should be clear and friendly. A clear explanation of the aim of the survey should be provided as well as specific answers to possible questions.
- During the *personal contact* with the headmaster make sure you are well organised to provide then with the necessary documents.
- There should be clear agreements with the teacher about the procedure for taking the parent questionnaire to home and receiving this in completed form returned from the children. This in order to obtain a high response rate for the parent questionnaire.

Timeframe

Schools should preferably be contacted in October/November/December 2009 with an initial letter to the headmaster of the sampled schools. The acquiring of schools should be finalized by **mid-February**, one month before the official start date of the cross sectional survey.

After school recruitment all class teachers should be provided with written information about the project and instructions about the implementation and timing of the survey in classes. Furthermore, parent information letters should be sent out in order to inform the parents about the project and consent to their child's participation in the survey.

The cross sectional survey should be carried out in all countries from mid March to mid May 2010. Some partners need to consider in their planning the Easter school break, spring break or other established holidays. All partners should provide ResCon with the schedule for all schools that has been made for the survey. This is important because visits for a procedural check will be planned by ResCon.

D. Preparation of documents

Letter to headmaster (Appendix A)

The initial contact with the school will be a formal letter to the headmaster of the sampled school. The letter will describe the aim of the overall ENERGY project and the purpose of the cross European survey in particular. The schools will be informed that the study aims to get up-to date information about height and weight of the children, the amount of time spent on specific energy balance-related behaviours, and information on why children spend more or less time on these specific behaviours. Besides the questionnaire for parents

and children, also the environment and school policy investigation through a checklist and questionnaire for the school management will be mentioned. The letter will also explain that all information gathered will be treated anonymously and that information specific for their school will not be available for other schools. It will also explain that the questionnaires need to be completed during one school hour and will thus replace a regular school hour. An almost identical copy can be sent to the school board if required, depending on local circumstances.

Letter to parents (Appendix B)

Following the acceptance of the school to participate in the survey, parents will receive a letter explaining the purpose of the survey and will be asked for written consent for the participation of their child and themselves in the ENERGY project. The letter will be brought home by the child and returned by this child back to school. If no consent is available for a child, the child will not participate in the study and will not be asked to complete the questionnaire and no measurements will be taken.

The letter will inform them on the influence of family environment on the child's health behaviours and therefore ask them to complete a questionnaire as well. Also clarifications will be provided regarding the anthropometric measurements that are going to be obtained, how long the collected data will be kept for, that all data will be treated anonymously, that they can withdraw any moment, that they are not obliged to participate and that no home addresses will be entered in the database.

Translation of child and parent questionnaires into local languages

The introductory section and the items of the child and parent questionnaire have to be the same for all participating countries. Therefore, the questionnaires will be developed in English, translated into the language of each participating country and then will be back translated in order to detect any potential differences between the back translated and the original questionnaire. The back translation should be carried out either by a translation office or by a native English speaker who is not involved in the project. The back-translated version of the questionnaires will be sent to ResCon for cross- checking.

Lay out

The layout of the child and parent questionnaire will be done centrally by the same company (Arthleten) in order to assure a consistent form of appearance, a reasonable progression of questioning and the same format to be used for scanning of the questionnaires.

Audit instrument and School management questionnaire

The audit instrument and school management questionnaire will be translated in local languages. Every effort has to be made to avoid differences in meaning and interpretation of words and phrases respectively. The participating countries are responsible for the accurate translation of these instruments and ResCon coordinates the lay out procedure.

Pre-testing in UK, Greece and the Netherlands

The English version of the child questionnaire will be pre tested in the UK during weeks 48-51. The English version of the parent questionnaire will be pre tested at native English speaking parents in Greece and the Netherlands during weeks 45-51. The aim of the pre-testing is to get more insight into the clarity and structure of the questionnaires.

Pre-testing in all countries

The child and parent questionnaires (Appendix C and Appendix D), audit instrument (Appendix E) and school management questionnaire (Appendix F) will be pre-tested in native languages in all countries based on pre-testing protocols. The aim of the pre-testing is to improve those tools on phrasing and content.

The pre test will start at the 8th of February 2010 and will end at the 19th of February 2010. These two weeks do include the writing of this report including the results, conclusion and recommendations.

Test – retest

The test re-test of the child and parent questionnaires, audit instrument and school management questionnaire will take place during weeks 11-19.

Anthropometry training

At the ENERGY Ghent meeting at the beginning of February, all researchers are trained for the anthropometry measures. After this training, in each country a training of the research assistants, by the researchers who had the training at the Ghent meeting, will take place before the beginning of the measurements. Inter- and intra- reliability test will be performed and the results will be sent to HUA before the official start day of the survey.

E. Field procedures

Send-out of letters – timeframe

The school recruitment letter should preferably be sent out in October-December 2009 to the headmaster of the sampled schools. It is recommended to give a personal call shortly after in order to discuss further details of the survey and to arrange a meeting. The time period between reception of the letter and the follow-up telephone call can vary between countries but should ideally be between 1 to 2 weeks. The reasons for non-participation can be asked for during this call.

Meeting with the headmaster and instruction of teachers – timeframe and informed consent procedure

A meeting with the headmaster will follow in order to answer any potential enquiries and inform on the timeframe of the survey in their school.

Each class teacher will get written information about the project, i.e. a copy of the headmaster's letter, with general instructions about the implementation and timing of the survey. Preferably both the researcher and the teacher will be responsible for handing out the parent information and consent form letter to children. The teacher will have a supportive role in encouraging children to take part and the researcher will provide the background information. Teachers will also be responsible to retrieve the parent questionnaire within a couple of days. The researcher will ring the school the day before picking up the consent forms, so that the teacher will give a last reminder to children to bring it back.

Questionnaires

Researcher should be prompted not to hand out the questionnaires on a Monday, since we do not want to collect Sunday information on food and drink intake. Each country takes care for their own envelopes to put the parent questionnaire inside and be able to give this to the child. The identification number should be written down by the researchers on the child and parent questionnaires.

IMPORTANT

The researchers are advised to write down the identification number (except for the land code which will remain the same for every country) on the child and parent questionnaire when they are handing out the questionnaire to the child. This way if a child is missing or decided to drop out the child and parent questionnaires can be used for another child/parent.

To avoid that the identification number will be lost due to ripping off the first page, this number should also be written on the final page (right above the "Thank you for completing this questionnaire") of the questionnaires for children and their parents. No names will be written anywhere. The researcher has to underline to children not to mix up or swap their envelopes.

The identification number consists of:

- Land code: **1 digit**

Country	Land code
Belgium	1
Greece	2
Hungary	3
Netherlands	4
Norway	5
Slovenia	6
Spain	7
Switzerland	8

- School number of the child: **3 digits**; 1 for the tertile/region and maximum of 2 for the school
- Class/Grade number of the child: **1 digits**; 5th or 6th grade
- Child number: **2 digits**; there will probably not be more than 35 children in one class

- The identification number will be the same for the child and the parent questionnaires. Extra questionnaires should be taken with you to the school, due to new students appearing and lost questionnaires from parents.

Matching Data

The researcher will:

- Either make a list of the children's' name which will correspond to the identification number and just ask the child's name and provide him/her with the right questionnaire.

OR

- Provide children with stickers based on their identification numbers and distribute the questionnaires. This method is mostly preferred since it will be easier for the researcher to identify the child throughout the measurements.

Delivery of the Questionnaires

All WP7 partners will provide ResCon with the contact person and the address so that the questionnaires are expected to arrive more safely and quickly. Each country will receive two times 1200 questionnaires between week 10 and 13, depending on the start of the data collection. When more are needed, the country has to let ResCon know at the 26th of February at the latest how much extra questionnaires need to be printed at the Dutch printing company. Questionnaires will not be printed at the countries itself. The rationale for this is that the questionnaires have to meet the specific criteria of the company who will scan al the questionnaires.

On the day of the survey the researcher does the following:

1. **Provide children with detailed instructions;** Distribute the child questionnaire and explain the purpose of the study to the children. Make sure that children do not swop the questionnaires because they are coded individually. She/he will tell the children that the results of the study will help to make all children in Europe healthier, and therefore it is important to give honest answers, that they will not be judged, that all their answers will be treated anonymously, that their parents, teachers and class mates

will not be informed about their answers and that they can withdraw any moment during the study.

2. **How to fill in each section;** Read out the standardized instruction form to the children on how to fill in the questionnaire in sections (Appendix G).
3. **Collect the children's questionnaires**
4. **Envelope with parent questionnaire;** Give to children an envelope with the parents' questionnaire to give it to their mother or father and to return it to the teacher by a specific date.

The time needed for the entire procedure is approximately *one school hour.* However, children should be aware that there is a possibility for them to remain in the class during recess to finish the questionnaire.

Responses to questions from pupils

Some pupils may have problems in understanding (parts of) the questions. Researchers can help students individually if major problems occur but are urged to keep interaction to a minimum to reduce between country variations.

Collection of envelopes by the teachers

By a specific date the teachers should collect the parent questionnaires. The research team will ring the school on that date to arrange a time to receive the envelopes either by hand or by post. If children do not bring the questionnaires back within a week the teachers will be responsible to notify researchers to receive the late returned envelopes.

Anthropometry measurements

2 researchers will be needed in order to conduct the anthropometry measurements. Researchers will have already entered the school and child number on the anthropometry datasheet provided by VUmc. For matching data they can either have a list of names which will correspond to the identification number (same as questionnaires) or put stickers on children based on their identification numbers.

The anthropometry protocol (Appendix I) will be followed for the accurate conduction of the measurements. Researchers will enter the anthropometry measurements by hand and fill in the data by hand (Appendix H). The researchers will receive an excel datasheet by VUmc to insert the data.

Those researchers should process the data in the excel file on the same day after the completion of the measurements. The identification number should be entered for each child.

The following measurements will be taken:

- Waist circumference
- Height
- Weight

Absentees

Children absent at the day of collection are disregarded from the survey.

School management questionnaires

On the survey day the school management questionnaires will be given separately to each headmaster. On the day of receiving the parent questionnaires the researcher will also receive the completed school management questionnaire. The researcher will receive an excel datasheet by VUmc to insert the data.

Audit instrument

The observations of the school environment will be done by two observers on a separate date that can be arranged between the researchers. The researcher will receive an excel datasheet by VUmc to insert the data.

Quality check

Quality check on data insertion will be performed. 10% of the anthropometry measurements will be entered in the excel sheet by two separate researchers. Then the researchers will cross check if they have noted the same values.

Also 20% of the audit instrument and the school management questionnaire will be entered in the excel files by two separate researchers. Then the researchers will cross checked if they have inserted the same data.

If there is no good agreement then all data should be re-entered.

F. Accelerometer study

Another special feature of this cross-European survey will be the use of accelerometers to objectively measure physical activity in Greece, the Netherlands, Belgium and Switzerland.

The schools for the accelerometer data collection will be selected from the list of randomly selected schools as sent by ResCon. We aimed to collect accelerometer data from at least 200 children per country from 4 schools (50 students per school). The selection of schools should be balanced across the 3 cities (selected from 3 tertiles), which were selected for the main survey. Distribution of the number of selected schools for accelerometer study should be proportional with the number of schools in the selected cities (thus more schools from larger cities).

One identical recruitment letter will be sent to all randomly selected schools explaining the possibility that some schoolchildren can be asked to wear accelerometers during one week. The selection of the four schools for accelerometer data collection will be based on the logistic planning of the survey data collection process (Appendix F).

Research assistants will be trained during the project meeting in Gent on the 5th of February and are responsible for the accelerometer data collection according to standardised protocols. Accelerometers should be initialized and prepared before handing out in schools. Information about accelerometer use will be given to schoolchildren and at the end of the information session, accelerometers will be handed out. Additionally, children and parents will receive a brochure about accelerometer use and a diary. Teachers will also be informed about the procedures and asked to remind the children to wear the devices every day. Children will bring the device back to school and hand it to their teacher. Accelerometers will be collected from schools the day after. They will be prepared for the next school, i.e. charging, downloading data, initializing. In the end it is expected to have data from 800 children (200 per country).

G. Data handling and entry

Data delivery

Questionnaires

By the **14th of May** the data collection period should be completed. Partners are not allowed to make copies of the questionnaires before sending it to the scanning company in the Netherlands since it can cause problems with scanning. Each country has two weeks' time to pack the child and parent questionnaires and either send them by registered mail directly to the scanning company or transfer them themselves. **The scanning company should have all questionnaires delivered by the 31st of May.** Either way partners are responsible for the safe and on time delivery of the questionnaires to the Scanning company in the following address:

Scan Serv
t.a.v. Johan Almeida
ENERGY project
Hofweg 21
2631 XD
Nootdorp
The Netherlands

It should be noted that researchers should estimate the time that the delivery will take from their country to the scanning company.

Excel files

By **Monday 31st June** all partners should email the excel files for the Audit instrument, School management questionnaires and anthropometry data via "YouSendIt" to ResCon.

Data entry

Data entry from the parent and child questionnaires into SPSS files will be done automatically by the Scan Serv company. The data entry from the audit instrument and school management questionnaire and anthropometry measures will be done by the researchers in each country itself in the excel files. The anthropometric measurements data, results from the audit instrument and school management questionnaire will be transferred from the excel file to the SPSS file by the VUmc data manager.

Identification

Country number (1 digit), School number of the child (3 digits), Class/grade number of the child (2 digits), Child number (2 digits), as separate variables.

Missing information

All questionnaires will be scanned and entered in a data set. This data will be cleaned afterwards at the VUmc. The same cleaning procedure accounts for the anthropometric data, the audit instrument and the school management protocol.

Backup procedures

Data should be saved on local server hard-disk drives, with a copy on one or more local workstation drives. Additional copies will be made on DVD and will be spread among all partners.

Data Cleaning

A trouble-shooting list will be prepared for all data files by the VUmc.
Scan Serv will deliver a data file one week after the scanning can start of all questionnaires. When the data is available, the VUmc will start the data cleaning process and afterwards, on request, data files are sent.

Data storage and safety

All the hard copies of the anthropometry measurements, the child and the parent questionnaires, the audit instrument and the school management questionnaire will be stored for 5 years after the project ends.
The scanned data will be stored in DVDs/CDs at the VUmc, which will be available to partners upon request by sending a mail to the VUmc.
The hard copies of the anthropometry data, audit instrument and school management questionnaire should be sent by all partners to VUmc on the 11th of June at the latest.
Data storage and safety will be co-ordinated by WP11.

Feedback to schools

Feedback to schools can be provided upon request by the headmaster to increase participation rate. Schools will be given the average BMI of the 5th and 6th graders and also a short report of the dietary and physical activity of this age group.

Appendix A Letter to headmaster

[School address]

[Place], [Date]

Dear [Headsname],

This letter is send to your school to invite you to participate in a project entitled:

ENERGY Project
(E**uropea**N E**nergy balance** R**esearch to prevent excessive weight** G**ain among** Y**outh)**

The ENERGY project is supported by the European Commission and carried out across 8 European countries: Belgium, Greece, Hungary, Norway, Slovenia, Spain, Switzerland and the Netherlands. Participation is requested of pupils aged 10-12 years old and one of their parents/caretaker.

Background and relevance
In Europe, more than 400.000 children are overweight or obese. This prevalence is still increasing, reaching epidemic proportions. Overweight is caused by an imbalance of nutrition intake and physical activity, so called energy balance behaviours. The transition from childhood to adolescence (age 10-12) is a critical period as they gain more autonomy regarding these behaviours. There is an increase of computer use, drop out from sport clubs, skipping breakfast, snacking and sugar-sweetened drink consumption. Overweight is a strong predictor for being overweight at adulthood. It furthermore is associated with medical and psychosocial problems like asthma, Diabetes Mellitus type 2, depression, bullying and worse performance in class. Prevention of overweight at an early age is therefore of great importance.

Aim
The overall aim of the project is to obtain insight into the energy balance behaviours and their determinants in children aged 10-12 years olds, with the intention of developing an effective obesity prevention intervention.

Reason participation of your school
The school is, next to family environment, of major importance to children's health and an ideal setting for health promotion and education. Participation with this European project will provide insight into the drinking and food habits and physical activity patterns of your pupils and into the prevalence of overweight and obesity at your school. As the main objective is to design an obesity prevention intervention, your school can contribute to the prevention of overweight and obesity and therefore is relevant for current and future health of the schoolchildren.

Comment [V1]: The underlined parts concern ONLY Belgium, Greece, the Netherlands and Switzerland.

Measurements and project time line

During April/May 2010 the following measurements will take place:

- The schoolchildren are asked to complete a written questionnaire related to energy balance behaviours.
- The schoolchildren's' parents (one parent/caretaker per child) are asked to complete a written questionnaire related to energy balance behaviours.
- Measurements on the schoolchildren's' height, weight and waist circumference will be performed.
- In some schools children are asked to wear an activity meter during 5 days.
- An audit instrument will be completed by the research assistant including a short school staff questionnaire to provide an overview of the school environment.

Expectations of school and teachers

The teachers of the pupils aged 10-12 years old are asked to reserve one hour for the completion of the questionnaire, co-ordinated by a trained research assistant. Furthermore, children are asked to bring the parental questionnaire home and return the completed version to the teacher. The physical measurements of the schoolchildren will approximately take two school days, in concordance with the school. Some children are asked to wear an activity meter for 5 consecutive days. The device is the size of a matchbox and worn in an elastic belt on the right hip. All measurements will be performed by a trained research assistant.

Confidentially

All data collected within the project will be anonymous and stored in locked filing cabinets and password protected computers at [name University]. After the project is completed all the study materials and information will be stored securely for up to five years by [name University] and then destroyed. Schools will be named during report writing, although only as an acknowledgement for taking part. Names of schools will never be attached to specific data. Anonymous identifiers (e.g. School 'A'; School 'B') will be used when discussing the data. When interested, a written summary of the project will be available for the participating schools.

Voluntary

Participation of your school, the children and their parents is voluntary. You are free to withdraw from the study at any given time, without giving a reason. Choosing not to participate or withdrawing from this study will not have any consequences whatsoever.

Contact details for questions and further information

For any further information you may require, please contact at working days:

Name: [Name responsible person at University]
Telephone: ______________________________
E-mail: ______________________________

HEAD TEACHER'S CONSENT FORM

ENERGY (Europea**N E**nergy balance **R**esearch to prevent excessive weight **G**ain among **Y**outh**)**
Project

Please read the statements below (ticking box 'Y' or 'N' and initialling each statement) and complete the form at the bottom of the page if you would like your school to take part in this project.

	Yes	No	Initial
I confirm that I have read and understood all of the information relating to this project and have had the opportunity to ask questions (face to face and by telephone).	☐	☐	☐
I understand that the school's participation is entirely voluntary and we can stop participating at any time, without giving a reason before the 30th of April when the data collection period ends.	☐	☐	☐
I understand that if the school withdraws from the project, all data collected up to the point of withdrawal will be kept and used.	☐	☐	☐
I understand that all participant information will be anonymous and kept confidential. All data collected will be stored securely at [name University] with computerised information being stored on password protected computers. After the project is completed all the study materials and information will be stored securely for up to five years by the VU University and then destroyed.	☐	☐	☐
I understand that the findings of the project may be printed in	☐	☐	☐

journals and talked about at conferences, but that pupils will not be identified in any report or journal article, although the school may be named without attachment to data.			
I understand that parents will be asked to provide consent for their child to participate in the project, and that pupils (whose parents have given consent) will be asked to give their assent to participate and that the school should not encourage or discourage anyone to take part.	☐	☐	☐
I confirm that my school will take part in this study.	☐	☐	☐

Name of school: [*FILL IN*]

Name of head teacher: ………………………………………………………………

Signature of head teacher: ………………………………… **Date:** ……/……/………

Appendix B Letter to parent

Comment [V2]: The underlined parts concern ONLY Belgium, Greece, the Netherlands and Switzerland.

ENERGY

Information letter for parents/ caretakers

Why?

During the last decades, there is an alarming increase in the number of European children that are overweight. Food habits and physical activity patterns are key issues of health and may have an impact during lifetime. This means that obesity prevention can not start early enough. The transition from childhood to adolescence (age 10-12) is a critical age period regarding energy balance behaviors: drop out from sport clubs, increase of computer use, increase of snacking, soft drink consumption and skipping breakfast. Intervening in that age is therefore important. To develop an effective intervention it is important to get more insight in the energy balance behaviors of children.

Aim of the study

The aim of the study it is to get more insight in the school and family factors that influence energy balance behaviors in youth. These insights will enable the development of an obesity prevention intervention in European youth.

Why is my school participating in the ENERGY project?

The school is, next to family environment, of major importance to children ' s health. It is self-evident that the school is an ideal setting for health promotion and education. Your school wants to contribute to obesity prevention and is therefore willing to participate in the study.

Thanks to the schools' and your participation, effective obesity prevention interventions can be developed within ENERGY. Therefore this study is particularly relevant for future health of your child.

European

This study is supported by the European Commission by a specific project called ' ENERGY ' . The study project is carried out across 8 countries in the European Union: Belgium, Greece, Hungary, Norway, Slovenia, Spain, Switzerland and the Netherlands. About 8000 schoolchildren aged 10-12years are participating in the study.

Measurements

During April/May 2010 the following measurements will take place:

- Your child is asked to fill in a written (child) questionnaire related to energy-balance behaviors
- You are asked to fill in a written (parental) questionnaire related to energy-balance behaviors
- We will perform measurement on your child's' height, weight and waist circumference.
- <u>At some schools children will be asked to wear an activity meter during 5 consecutive days</u>

All child measurements will take place during school hours and you could fill in the parent questionnaire at home.

Confidentially

All information concerning your child ' s and your participation in the study will solely be used for scientific purposes, in accordance with applicable *national* law. Only the members of the research team have access to the data. The name of your child and / or your name will not be used in any report or released in any way. Names will be kept separately from any survey data under lock and key.

Voluntary

Your child ' s and your participation in this study is voluntary. Your child and you are free to withdraw from the study at any given time, without giving a reason. Choosing not to participate or withdrawing from this study will not have any consequences whatsoever.

Contact details

For any further information you may require, please contact at working days:

Name: ______________________________

Telephone: ______________________________

E-mail: ______________________________

Certificate of consent

I have read the information form and the information supplied is clear to me.

If I do have more questions, I know whom to call.

I agree with my child ' s participation in the study described in the information form and I am willing to participate in the study as well.

I have been informed that my child ' s and my participation in this study are voluntary. I am free to withdraw and / or my child from the study any time and without need to give a reason. Choosing not to participate or withdrawing from this study will not result in any disadvantages for me or my child.

Name of child (in block letters)

____

Name of parent/ caretaker (in block letters)

____

City, date

____

Signature parent/caretaker

Name researcher: ____________________

Appendix C

Child Questionnaire

Pre test protocol and report

[Country]

[name of authors/researchers]

[date]

Content **Page**
number

1. Introduction **27**

2. Method **27**

3. Results **27**

4. Conclusion and recommendation **28**

Appendix I **29**

Appendix II **31**

Appendix III **33**

Fieldwork Protocol

1. Introduction

Before the ENERGY data collection starts in mid March 2010, the pupil questionnaire needs to be pre-tested among children aged 10-12 years old in every country. By discussing the content of the questionnaire with the target group can lead to substantial improvements in phrasing and content.

The lay out of this report is the same for every partner involved in the ENERGY project and will consist of a description of the method (chapter two) that has been applied to perform the pre test. Except for the instruction, this part has to be completed by the researcher. Chapter three is a description of the results, which are based on the checklist. Chapter four includes the conclusions that can be drawn and the recommendations that can be given from the pre test results.

The pre test will start at the 8th of February 2010 and will end at the 19th of February 2010. These two weeks do include the writing of this report including the results, conclusion and recommendations. Friday the 19th at the latest you can send your report to Maria van der Sluis (mail to m.vandersluis@rescon.nl). The reports will be discussed during week 8 and based on the results minor changes will be made and the lay out will be finalized.

2. Method

Instruction:
In total five to ten children of age 10-12 years old will be requested to complete the questionnaire. They can be recruited either from one class or from different classes from a primary school (see Appendix I). An other option is that the children are recruited through personal relations, for example family or friends of the researchers. A pupil information sheet will provide information to pupils about the project and the pre testing procedure (see Appendix II).

(To completed by researcher)
- Method of recruitment
- Description of characteristics of respondents

3. Results

Please write down the results according to the checklist (see Appendix III).

A. General opinion about the questionnaire.
B. Comprehensibility and feasibility of the questionnaire in general.
C. Comprehensibility and feasibility of the questionnaire specific.
D. Design of the questionnaire

EXAMPLE

3.1 General opinion about the questionnaire

- What do you think of the questionnaire? (fun/boring/strange) *Try to get detailed information, like what was their first impression, when did they not find it fun anymore? Or which part or item?*

    a. 6 respondents mention that the questionnaire is “fun to complete” and “easy to read”.
    b. 4 respondents are “discouraged” from the fact that it would take one lesson to complete the questionnaire. From the second part on during the lesson they got bored and concentration decreased.

4. Conclusion and recommendations

Please write down the conclusions and recommendations based on the checklist results.

Appendix I

Recruitment letter of schools for the pre test of child and parent questionnaire

[Place and date]

Dear [name of the head of the school],

This letter is send to your school to ask you to participate in the pre test phase for the European project entitled:

ENERGY Project
(Europea**N E**nergy balance **R**esearch to prevent excessive weight **G**ain among **Y**outh)

The ENERGY project is supported by the European Commission and carried out across 8 European countries: Belgium, Greece, Hungary, the Netherlands, Norway, Slovenia, Spain and Switzerland.

Background and aim
In Europe, almost one in four children is affected by overweight or obesity. This prevalence is still increasing, reaching epidemic proportions. Overweight is caused by an imbalance of nutrition intake and physical activity, so called energy balance behaviours. The overall aim of the project is to obtain insight into the energy balance behaviours and their determinants in children aged 10-12 years olds, with the intention of developing an effective obesity prevention intervention. The school is, next to family environment, of major importance to children's health and an ideal setting for health promotion and education. Therefore a school and parental survey will take place in all eight countries from mid March to mid May 2010 and will comprise a pupil and a parent questionnaire.

Reason participation of your school
Before the data collection starts, the pupil and parent questionnaire need to be pre tested. For this reason we would like to test the questionnaires at five to ten children aged 10-12 years old and five to ten parents with children in the age category of 10-12 years old. The questionnaires will include additional questions about e.g. the structure, clarity and lay out. The children who complete the questionnaire will be asked to participate in a focus group. The parents will be kindly requested to complete the questionnaire and, if the parent agrees, to discuss the content of the questionnaire in a telephone interview.

Time line
Completion of the questionnaire, the focus group and the telephone interview will take place during weeks 6 and 7 (8th of February – 16th of February 2010). The children can complete the questionnaire in a small group and will be requested to take part in a focus group.
In order to recruit a minimum of 5 parents we would like to ask the pupils of the 5th and 6th grade, and possibly other grades, to take the information letter and the questionnaire at home and to bring a completed version back to school. We will provide the school teacher with the letters and questionnaires (week 5) and will collect the completed

versions (Early week 6). In the information letter to the parent, the parent will be asked if we can approach him or her by telephone to discuss the questionnaire.

Voluntary

The participation of the parents and children is voluntary. They are free to withdraw from the study at any given time, without giving a reason. Choosing not to participate or withdrawing from this study will not have any consequences .

Contact details for questions and further information

We will contact you by telephone if you would like to participate. For further information you may require, please contact at working days:

Name:
Telephone:
E-mail:

Kind regards,

[name researcher]

Appendix II

Pupil Information Sheet

ENERGY Project

A team of people from [name] University want to find out more about the food habits and physical activity patterns of 10-12 year-old children in a project known as ENERGY (**E**uropea**N** **E**nergy balance **R**esearch to prevent excessive weight **G**ain among **Y**outh). To do this, they have developed a new questionnaire which needs to be tested on children who are 10-12 years old.

What is research and why is this project being done?
Research is a way we try to find out the answers to questions. We would like to know your opinion about our new questionnaire, so we can improve it for the future.

What will happen to me if I take part in the research?
We would like to know what you think of our new questionnaire so we can improve it and make it easier to understand. To be able to do this we need your help. Before you and your parents decide if you want to take part or not we would like to ask you to read carefully the information on these two pages and talk about it with your family and friends if you want to.

1. What the questionnaire is about: We would like you to tell us about the kinds of foods you eat, what foods you like, what foods you don't like, what activities you are involved in, how often you watch television… all to give us a better understanding of how you and other people your age behave.

We also want you to mark any questions that you do not understand

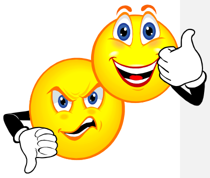

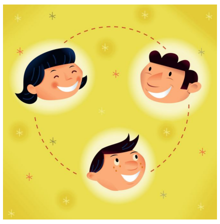

2. Discuss the questionnaire: When you complete the questionnaire we will ask you what your thoughts are about the questionnaire, and to tell us about the bits you liked and the bits that you didn't like, how well you understood what the questions were asking and in general what was your impression about the questionnaire

Why have I been asked to take part?
You are being asked to take part because your school has agreed to take part in this project. Some other 5th and 6th grade pupils in your school have been invited to participate as well.

Did anyone check the project is OK to do?
Before any research is allowed to happen, it has to be checked by a group of people called a Research Ethics Committee. They make sure that the research is fair. This project has been checked by an ethics committee at [name] University.

Do I have to take part?
No you do not have to take part in the project. It is up to you and your parents to decide if you want to or not. Also, if you decide to take part and then change your mind at any time this is fine, just let the researchers know you do not want to carry on.

When will this project start and finish?
The project will run during February.

What are the possible benefits of taking part?
The information gained from this project will help us make the questionnaire easier to understand, so we can use it with other schoolchildren who have the same age as yours.

Might anything about the research upset me?
We hope that nothing we do will be upsetting for you but if at any point you would like to talk about anything that is bothering you, you can tell it to the researchers, your teachers, or your parents who will be happy to help you with any problems that you may have.

What will happen when the research stops?
When the research stops we will write a report to a group of people. The report will let these people know whether anything about the questionnaire needs to be changed to make it easier to understand. The new questionnaire will then be given to 8,000 schoolchildren in eight different European countries with similar questions to the ones we have asked you, so we can find out more about their eating and activity habits.

Will my details and the information I give be kept private if I take part? Will anyone else know I am doing this?
All the information that you give us will be kept private and will not be shown to your parents, teachers and your friends. You can tell others that you are taking part if you want to. Don't forget that you can ask questions anytime about the project any of the project staff as well as your family.

If you have any questions, then please ask your parents/guardians/carers, your teacher or the researchers when they are at school.

Thank you!

Appendix III

Checklist pre test focus group pupils

ENERGY Project
(**E**uropea**N** **E**nergy balance **R**esearch to prevent excessive weight **G**ain among **Y**outh)

*A team of people from [name] University want to find out more about the food habits and physical activity patterns of 10-12 year-old children in a project known as ENERGY (**E**uropea**N** **E**nergy balance **R**esearch to prevent excessive weight **G**ain among **Y**outh). To do this, they have developed a new questionnaire which needs to be tested on children who are 10-12 years old.*

Aim of Focus groups
The focus group will provide the researchers with further feedback on the questionnaire in terms of comprehensibility, feasibility and design of the questionnaire.

Introduction (5 min)
You all just completed a questionnaire about your drinking and food habits and in which activities you are involved in. The reason for this is to give the team of people from the [name] University a better understanding of how you and other children of your age behave. You already have marked questions which you did not understand. Nevertheless, we now would like to talk about what you think about the questionnaire in a more extensive and detailed way. We will not specifically talk about the answers you gave to the questions but for example which part you did like or did not like, or if you think the questionnaire was fun to complete or not. This way we can improve the questionnaire. This is important because it will make it easier for other children that will be asked to complete the questionnaire in the future. This year the questionnaire will be completed by 8,000 pupils in eight different countries.

Everybody can respond to our questions, and you can respond to what each other is saying, but it is very important that you speak one by one. There are no right or wrong answers. So if your classmate is saying something you do not agree with, you can also give your opinion afterwards. It is also possible that you give your opinion but that you think friends of your age or classmates would disagree or think differently. You can mention that too. Do not hesitate to ask questions during this hour and to tell us what you think. All the information that you give us will be kept private and will not be shown to others. Are there any questions beforehand?

Questions
<u>A General opinion about the questionnaire (10 min)</u>

- What do you think of the questionnaire? (fun/boring/strange) *Try to get detailed information, like what was their first impression, when did they not find it fun anymore? Or which part or item?*
- Do you think that children of your age will complete this questionnaire honestly? Did you complete the questionnaire honestly?
- Did you answer any questions by luck because you did not know the answer or you were bored? If yes, which ones?

<u>B Comprehensibility and feasibility of the questionnaire in general (10 min)</u>

- What do you think of the length of the questionnaire? When it is too long, which part or from which part on?
- What do you think of the sequence of the questions?
- What questions or parts did you find difficult to complete?
- What questions were easy to complete?
- What do you think of answering questions by putting an ‘x’ in the box?
- What questions did you not understand? What exactly was not clear to you?
- Were there any questions that you thought about but were not asked? If yes, on what topic?
- If you answered a question, were there sometimes answers missing?
- Did you find any errors in the questionnaire? If so, where?

<u>C Comprehensibility and feasibility of the questionnaire specific (20 min)</u>

- Did you read the instruction ‘How to complete the questionnaire’?
  - If no, why not?
  - If yes, was the instruction clear enough?
- Do you know the year and month you were born? (this concerns question A1 and A2)
- Do you understand what the researchers mean with the text ‘what you usually drink or eat’? (this concerns the text box after question A7)
- Some questions are about your mother, what do you think about that? *No problem, strange, annoying, could also be father or both, etc.* (This concerns question B6, B8 etc)
- Did you recognize the soft drinks and fruit juices that were mentioned in the answers? Were there any strange drinks mentioned or other drinks that you missed? (this concerns text box before question B1 (soft drinks) and before question C1 (fruit juices)
- Did you find the definition on breakfast eating clear? (this concerns text box before question D1)
- What do you think the researchers meant by ‘physical activity’? (this concerns text box above question E1 and further questions)
- What do you think the researchers meant by ‘sport activities’? (this concerns text box before question E9 and further questions) (is this clear or is the term ‘organized activities’ clearer?)
- Do you have a favourite sport? Or second favourite sport? (Question E10 and E14)
- Who decides whether you can take part in sports or other physical activities?
- If a child usually watches 2 hours of television on a Saturday and 5 hours of television on a Sunday, which text box does he or she need to tick? *(To check if they fill in the total hours watched across a weekend or the average across a weekend)* (this concerns question F1).

<u>D Design of the questionnaire (10 min)</u>

- What do you think about the design of the questionnaire? (fun/boring/strange)
- What do you think of the Energy logo used in the questionnaire?
- What do you think about the general illustrations used in the questionnaire? (fun/boring/strange)
- What do you think about the graphic designs of the bottles used in the questionnaire? (this concerns question X) (recognizable or not)
- What do you think about the text boxes used in the questionnaire?

- Is there anything you think that would make the questionnaire more fun or easier to complete?

Finish and thank you (5 min)
This is the end of the interview. Does anyone have a question before we finish this interview? If not, we would thank you again for participating in this interview and completing the questionnaire.
Appendix D

Parent Questionnaire

Pre test protocol and report

[Country]

[name of authors/researchers]

[date]

Fieldwork Protocol

Content

1. Introduction..37
2. Method 37
3. Results 37
4. Conclusion and recommendation...38
Appendix I...39
Appendix II 41
Appendix III 43

Fieldwork Protocol

1. Introduction

Before the ENERGY data collection starts in March, April and May 2010, the parent questionnaire needs to be pre tested among parents from children of age 10-12 years old. By discussing the questionnaire with the target group, the questionnaire can be improved on phrasing and content.

The lay out of this report is the same for every partner involved in the ENERGY project and will consist of a description of the method (chapter two) that has been applied to perform the pre test. Except for the instruction, this part has to be completed by the researcher. Chapter three is a description of the results, which are based on the checklist. Chapter four includes the conclusions that can be drawn and recommendations that can be given from the pre test results.

The pre test will start at the 8th of February 2010 and will end at the 19th of February 2010. These two weeks do include the writing of this report including the results, conclusion and recommendations. Friday the 19th at the latest you can send your report to Maria van der Sluis (mail to m.vandersluis@rescon.nl). The reports will be discussed during week 8 and based on the results minor changes will be made and the lay out will be finalized.

2. Method

Instruction:
In total five to ten parents who have children of age 10-12 years old will be requested to complete the questionnaire. These can either be recruited from one class or different classes from a primary school (see Appendix I). An other option is that the children are recruited through personal relations, for example family or friends of the researchers. A parent information sheet will provide information to the parents about the project and the pre test (see Appendix II).

(to complete by researcher)
- Method of recruitment
- Description of characteristics of respondents

3. Results

Please write down the results extensively according to the checklist (see Appendix III).

A. General opinion about the questionnaire.
B. Comprehensibility and feasibility of the questionnaire in general.
C. Comprehensibility and feasibility of the questionnaire related to the remarks of the parents.
D. Comprehensibility and feasibility of the questionnaire specific.
E. Design of the questionnaire.

EXAMPLE

3.1 General opinion about the questionnaire

1. What do you think of the questionnaire? What was your first impression? *Try to get detailed information.*

   c. 6 respondents report that the questionnaire is “well laid out”, “easy to read”, “well presented”, “fine” and “concise”.
   d. 3 respondents are initially “discouraged” from the fact that it would take 45 minutes to complete the questionnaire. However, once they started answering the questions “time went through very quickly”.
   e. 1 respondent is “positively influenced” by the fact that she was contributing to a research study for the University. She is impressed by with the booklet format.

4. Conclusion and recommendations

Please write down the conclusions and recommendations based on the checklist results.

Appendix I

Recruitment letter of schools for the pre test of child and parent questionnaire

[Place and date]

Dear [name of the head of the school],

This letter is send to your school to ask you to participate in the pre test phase for the European project entitled:

ENERGY Project
(Europea**N E**nergy balance **R**esearch to prevent excessive weight **G**ain among **Y**outh)

The ENERGY project is supported by the European Commission and carried out across 8 European countries: Belgium, Greece, Hungary, the Netherlands, Norway, Slovenia, Spain and Switzerland.

Background and aim
In Europe, almost one in four children is affected by overweight or obesity. This prevalence is still increasing, reaching epidemic proportions. Overweight is caused by an imbalance of nutrition intake and physical activity, so called energy balance behaviours. The overall aim of the project is to obtain insight into the energy balance behaviours and their determinants in children aged 10-12 years olds, with the intention of developing an effective obesity prevention intervention. The school is, next to family environment, of major importance to children's health and an ideal setting for health promotion and education. Therefore a school and parental survey will take place in all eight countries from mid March to mid May 2010 and will comprise a pupil and a parent questionnaire.

Reason participation of your school
Before the data collection starts, the pupil and parent questionnaire need to be pre tested. For this reason we would like to test the questionnaires at five to ten children aged 10-12 years old and five to ten parents with children in the age category of 10-12 years old. The questionnaires will include additional questions about e.g. the structure, clarity and lay out. The children who complete the questionnaire will be asked to participate in a focus group. The parents will be kindly requested to complete the questionnaire and, if the parent agrees, to discuss the content of the questionnaire in a telephone interview.

Time line
Completion of the questionnaire, the focus group and the telephone interview will take place during weeks 6 and 7 (8th of February – 16th of February 2010). The children can complete the questionnaire in a small group and will be requested to take part in a focus group.
In order to recruit a minimum of 5 parents we would like to ask the pupils of the 5th and 6th grade, and possibly other grades, to take the information letter and the questionnaire at home and to bring a completed version back to school. We will provide the school teacher with the letters and questionnaires (week 5) and will collect the completed

versions (Early week 6). In the information letter to the parent, the parent will be asked if we can approach him or her by telephone to discuss the questionnaire.

Voluntary
The participation of the parents and children is voluntary. They are free to withdraw from the study at any given time, without giving a reason. Choosing not to participate or withdrawing from this study will not have any consequences .

Contact details for questions and further information
We will contact you by telephone if you would like to participate. For further information you may require, please contact at working days:

Name:
Telephone:
E-mail:

Kind regards,

[name researcher]

Appendix II

Information letter to parents

[Place and date]

Dear Madame/Sir,

This letter is send to you to ask you to participate in the pre test phase for the European project entitled:

ENERGY Project
(**Europea**N E**nergy balance** R**esearch to prevent excessive weight** G**ain among** Y**outh)**

The ENERGY project is supported by the European Commission and carried out across 8 European countries: Belgium, Greece, Hungary, the Netherlands, Norway, Slovenia, Spain and Switzerland.

Background and aim
In Europe, almost one in four children is affected by overweight or obesity. This prevalence is still increasing, reaching epidemic proportions. Overweight is caused by an imbalance of nutrition intake and physical activity, so called energy balance behaviours. The overall aim of the project is to obtain insight into the energy balance behaviours and their determinants in children aged 10-12 years olds, with the intention of developing an effective obesity prevention intervention. The school is, next to family environment, of major importance to children's health and an ideal setting for health promotion and education. A school and parental survey about diet and physical activity habits will therefore take place in all eight countries in March, April and May 2010 and comprises a pupil and parental questionnaire.

Reason participation of you as a parent
Before the data collection starts, the parental questionnaire needs to be pre tested. We would kindly ask you to complete the questionnaire but also let us know if you have any problems with the understanding and interpretation of any of the questions. Completion of the questionnaire will take about 30 minutes of your time. Please put any of the following marks next to each question which, for any reason, is not clear to you:

U = unclear
W = weird question/formulation
M = answer is missing
O = other reason

Voluntary
The participation of the parents is voluntary. You are free to withdraw from the study at any given time, without giving a reason. Choosing not to participate or withdrawing from this study will not have any consequences whatsoever.
Questionnaire and telephone contact

You will receive the questionnaire on [please enter date]. You can return the questionnaire by putting this in the envelope and giving this to your child who also brought the questionnaire at home. To discuss the remarks that you have noted in the questionnaire and thus to discuss the questionnaire in more detail, we would like to contact you by telephone in week [enter week and date]. This will take about 15 minutes of your time.

Contact and sending the questionnaires

If you have any questions or would like to have further information please do not hesitate to contact me by email or telephone (your email address / your telephone number).

If you agree to participate, would you please forward your name and address to Teachers Name so we are able to send you the questionnaire? Thank you in advance.

Kind regards,

RESEARCHER'S NAME

Appendix III

Checklist Telephone interview

Introduction
Before we start the interview I want to thank you for your willingness to participate in the pre testing procedure of the questionnaire that we developed. As mentioned before in the information letter a school and parental survey about diet and physical activity habits will take place in eight countries in March, April and May 2010. Before the data collection starts, the parental questionnaire needs to be pre tested. You have already completed the questionnaire and the reason for this interview is to collect more detailed information in order to improve the questionnaire. This will approximately take 15 minutes. We will not specifically talk about the answers you gave to the questions but more in a general way. By this we can improve the questionnaire. Before discussing your remarks I have firstly some general questions. So you have any questions beforehand?

Aim
The aim of this interview is to get more insight into the clarity and structure of the parental questionnaire. Consequently, the questionnaire can be adapted and improved.

Items and remarks
[to be filled in according to the remarks on the completed questionnaire]
U = unclear
W = weird question/formulation
M = answer is missing
O = other reason

A General opinion about the questionnaire

1. What do you think of the questionnaire? What was your first impression? *Try to get detailed information.*

B Comprehensibility and feasibility of the questionnaire in general

1. What do you think of the length of the questionnaire? When it is too long, which part or from which part on?
2. Were there any questions that you thought about but were not asked? If yes, on what topic?
3. Were there any questions that you thought that were too personal and therefore found it difficult to answer?
4. Did you find any errors in the questionnaire? If so, where?

C Comprehensibility and feasibility of the questionnaire related to the remarks of the parents

1. Discuss the remarks that parents mentioned in the questionnaires. *Try to get detailed information so we have input for improvement of the questionnaire.*

D Comprehensibility and feasibility of the questionnaire specific

If not discussed before:

1. Did you read the instruction ‘How to complete the questionnaire’?
   a) If no, why not?
   b) If yes, was the instruction clear enough?
2. Did you read the short introductions at the beginning of each new topic?
   a) If no, why not?
   b) If yes, was the instruction clear enough?
3. Did you find the definition on ‘breakfast eating’ and ‘physical activity’ clear?

E Design of the questionnaire

1. What do you think about the design of the questionnaire? (fun/boring/strange)
2. What do you think about the general illustrations used in the questionnaire? (clear/fun/boring/strange)
3. Is there anything you think that would make the questionnaire more fun, clearer or easier to complete?

Finish conversation

Do you have any additional comments or remarks about the questionnaire?

Thank you very much for providing us with your comments and making time available for this telephone interview. It has been very helpful.

Appendix E

Pre test Audit instrument

Protocol and report

Fieldwork Protocol

ResCon

January 2010

Fieldwork Protocol

Pre test Audit instrument

Content **Page number**

1. Introduction **48**
2. Method **49**
3. Results **49**
4. Conclusion and recommendations **50**
Appendix 1 .... **52**
Appendix 2 .... **54**

Fieldwork Protocol

1. Introduction

This audit instrument is designed to assess the physical school environment and how it supports participation in healthy eating and physical activity. Furthermore, it identifies areas within the school environment that could be improved in order to promote healthier behaviours. This will enable the school to prioritize measurable and attainable goals that can provide the basis for developing and implementing healthy eating and physical activity initiatives. Besides this, observations can be compared among schools and other countries. Before the audit instrument is used broadly by researchers in the ENERGY project, it needs to be pre tested in order to improve its content and phrasing. Eventually, the audit instrument will provide information on the relevance and focus of environmental components of the ENERGY intervention.

The lay out of this report is the same for every partner involved in the ENERGY project and will consist of a description of the method (chapter two) that has been applied to perform the pre test. Except for the instruction, chapter two will be completed by the researcher. Chapter three is a description of the results, which are based on the observation results and the checklist. Chapter four includes the conclusions that can be drawn and recommendations that can be given from the pre test results.

The pre test will start at the 8th of February 2010 and will end at the 19th of February 2010. These two weeks do include the writing of this report including the results, conclusion and recommendations. Friday the 19th at the latest you can send your report to Victoria Maskini (mail to maskiniv@yahoo.com and cc to m.vandersluis@rescon.nl). The reports will be discussed during week 8 and based on the results minor changes will be made and the lay out will be finalized.

2. Method

Instruction

The recruitment of the school to test the audit instrument can be executed parallel to the pre test of the parent and child questionnaire. Only one school is needed. All the school observations will be performed by two observers separately. All observations will be performed by two researchers, preferably in the morning. By this the inter-rater reliability can be obtained. When possible for the categorical outcomes, Cohen's kappa will be calculated by you to provide insight into the agreement of observations. Try not to have too much of time difference between the observations of the two researchers. This avoids that two researchers complete the form differently due to differences in availability of products rather than due to differences between the researchers. To be able to improve the audit instrument, your opinion and experience will be asked for (Appendix I). The interviews with the responsible person of the canteen/school shop and the vending machine administrator will be performed by one researcher only. After the interview you can ask some questions to the person of the canteen/school shop and the vending machine administrator evaluate the interview (Appendix II).

(to complete by the researcher)

Please write down the characteristics of the school if you have this information avaiblable.

- Public/private, urban/rural, size (number of pupils, size of school grounds), % low SES, % minorities.

3. Results

Please report you findings:

- Describe the similarities and differences in the observations between both researchers including the results of the Cohen's Kappa if you have calculated these.

- Your results from the checklist (Appendix I)

  A. General questions

  B. Specific questions

- Your results from the interview (Appendix II)

EXAMPLE:

<u>Inter-rater reliability</u>

At form 1B, x out of x are similar between observers. Cohen's Kappa was x for question x. Et cetera. Main differences are found in part x and x of form x.

<u>General questions:</u>

- How long were you busy with the completion of the forms and the interviews in total?
  On average it takes x hours and x minutes to compete all forms of the audit instrument including the interviews.

- Et cetera

4. Conclusion and recommendations

Please report your conclusions and recommendations based on both the inter-rater reliability test, the checklist results of both Appendix I and Appendix II.

Appendix I

Checklist audit instrument researchers

Instructions

The checklist needs to be completed by one or two researchers. In case of the latter you need to obtain agreement and not write down several opinions.

A. General questions:

5. How long were you busy with the completion of the forms and the interviews in total?
6. What do you think of the length of the instrument? When it is too long, which part or from which part on?
7. Were there any questions that you thought about but were not asked? If yes, on what topic?
8. What do you think of the sequence of questions and topics?
9. Did you find any errors in the questionnaire? If so, where?

i. Specific questions:

1. What percentage of the primary schools in your country do you expect to have a school canteen or shop?
2. What percentage of the primary schools in your country do you expect to have vending machines?
3. Do you find it useful to make pictures? If no, when and why not?
4. At the first section at page 2: “forms completed” when do you use the “no” answer and when do you use the “n/a” answer? (i.e. is the difference clear or should only “yes” and “n/a” will do)

5. Do you think that the person(s) was/were able to provide you with enough and reliable information? (interview with the responsible person of the canteen shop and the vending machine administrator, Form 1A and 2A)
6. Do you miss any topic or question during the interviews? (interview with the responsible person of the canteen shop and the vending machine administrator, Form 1A and 2A)
7. Were you able to obtain all information needed for the observation form from the canteen or school shop and vending machine? If not, which part was difficult or not feasible? (Form 1B and 2B)
8. Was the part with ‘country specific products’ feasible in practise for you? (Form 1B and 2B) If not, what could be improved?
9. Were did you obtain information for the subscription programs? (Form 3)
10. Did you miss the option the tick more than one box? (Form 3 and 4)
11. Did you experience any difficulties with the bicycle parking area questions? If yes, when and why? (Form 5)
12. Did you experience any difficulties with the observation form for physical activity equipment? If yes, when and why? (Form 6)
13. Did you experience any difficulties with the observation form for indoor sport facilities? If yes, when and why? (Form 7)
14. Did you experience any difficulties with the observation form for outdoor sport facilities? If yes, when and why? (Form 8)
15. Did you experience any difficulties with the observation form for the outdoor school area? If yes, when and why? (From 9)

Appendix 2

Checklist interview with person responsible for the canteen/food shop and the vending machine administrator. This concerns form 1A and 2A.

Introduction

Thank you for your willingness to participate in the pre testing procedure of the questionnaire that we developed. We just have spoken to you about the canteen/school shop and/or vending machine and I would like to ask you some questions about is to collect more detailed information in order to improve the interview. These will not be to talk about the answers you gave to the questions but more in a general way.

2. What do you think of the interview? What was your first impression? *Try to get detailed information.*
3. Were there any questions that you thought about but were not asked? If yes, on what topic?
4. Were there any questions that were difficult to answer because of lack of knowledge about it?

Finish conversation

Do you have any additional comments or remarks about the interview?

Thank you very much for taking the time to have this interview and providing us with your comments. It has been very helpful.

Appendix F

Pre test School management questionnaire

Protocol and report

ResCon

January 2010

Pre test School management questionnaire

Content **Page number**

1. Introduction........57
2. Method........57
3. Results........57
4. Conclusion and recommendation........58
Appendix I........59
Appendix II........61
Appendix III........63

Fieldwork Protocol

1. Introduction

The school management questionnaire is developed to describe the variation in food and physical activity of school environments and school policies. Before the ENERGY data collection starts in March, April and May 2010, the school management questionnaire needs to be pre tested among headmasters. By discussing the questionnaire with the target group, the questionnaire can be improved on phrasing and content.

The lay out of this report is the same for every partner involved in the ENERGY project and will consist of a description of the school characteristics (chapter two). Except for the instruction, this part has to be completed by the researcher. Chapter three is a description of the results, which are based on the results of the telephone interview. Chapter four includes the conclusions that can be drawn and recommendations that can be given from the pre test results.

The pre test will start at the 8th of February 2010 and will end at the 19th of February 2010. These two weeks do include the writing of this report including the results, conclusion and recommendations. Before the pre test the school management questionnaire need to be translated. This is without back translation. Friday the 19th at the latest you can send your report to Nanna Lien and cc to Maria van der Sluis (mail to nanna.lien@medisin.uio.no and m.vandersluis@rescon.nl). The reports will be discussed during week 8 and based on the results minor changes will be made.

2. Method

Instruction
In total 2-3 headmasters will be requested to complete the questionnaire. The questionnaire will be translated in native language. To guide translation, an extensive explanation on purpose and terminology use has been written (Appendix I). The recruitment of the schools to test the school management questionnaire can be executed parallel to the pre test of audit instrument and the parent and child questionnaire. In the recruitment letter can be added a short part which introduces the school management questionnaire and explains that this will also be pre tested. The person who is representative for school management (e.g. headmaster, adjunct-headmaster) will be kindly requested to complete the questionnaire. Afterwards, this person will be briefly interviewed by telephone contact to obtain detailed information about how the questionnaire could be improved on content and phrasing. An information letter will provide information to the headmaster about the pre test procedure (See Appendix II). This can either be send by post mail or by email.

(to complete by the researcher)
Please write down the characteristics of the schools and the characteristics of the persons who completed the questionnaire and has been interviewed.

- About the persons filling it in: position, gender, number of years at school (part A of the questionnaire)
- About the schools: public/private, urban/rural, size (number of pupils, % low SES, % minorities, (part A of the questionnaire).

3. Results

Please write down the results extensively according to the checklist (see Appendix III).

A. General questions
B. Specific questions related to the remarks of the headmasters
C. Design of the questionnaire

EXAMPLE

3.1 General questions

1. What do you think of the questionnaire? What was your first impression? *Try to get detailed information.*

Example of responses:

a. 2 respondents report that the questionnaire is "well laid out", "easy to read", "well presented", "fine" and "concise".
b. 1 respondent found the questionnaire too long.

4. Conclusion and recommendation

Please report your conclusions and recommendations based on the results of the interview with the school staff.

Recommendations for deleting
Recommendations for moving to audit
Recommendations for instruction/lay out changes
Recommendations for adding questions
Recommendations for improvement of questions for answer categories
Ideas for motivators to get it filled in and returned during the CSS

Appendix I

Terminology and brief explanation of purpose of each question in the ENERGY School management questionnaire.

Nanna Lien
20th of January 2010

1) Overall purpose:
To have the headmaster or someone else in the school management describe their school, the opportunities that the 5th-6th graders have to eat/drink and be physically active and generally how they think every day practice related to these two behaviours are in their school.

This means that we don't necessarily get a true picture of what is going on in the classrooms, but at this point in this research and within the ENERGY-project it is not feasible to ask every teacher involved with these grades.

2) General terminology to pay attention to when translating:
These are my definitions and clarifications based on things that I have learned, gotten feedback on or struggled with and NOT official definitions as I have not had time to search for that. If anybody have such definitions or disagree with the use here, please let me know. If you have questions about other terms, please e-mail me. As we have decided not to back-translate the questionnaire it is important that you do use this possibility to check with me if in doubt.

Pupils = used generally about all children attending the school

5th-6th graders = those grades or age groups participating in ENERGY – use the country specific terminology

Lessons = time scheduled for learning

Recess = free time between the scheduled lessons

Main meals/snacks = this is a difficult one.... Relevant main meals are breakfast, lunch, I think, but then the snack could have many names. In Norway this would typically be a "between meal" as the word snack is strongly associated with unhealthy foods, but here we try to have both healthy and unhealthy foods. It is also a point that there should be time scheduled for as we are targeting those eating and drinking occasions that school should have some responsibility for because they are planned as a part of the every day schedule.

Physical education = the subject taught in school

Sports = organized activities with regards to participating in a special sport, often competitive

Physical activity = all types of movements (for sports, transport, house chores, play, other unorganized activities like walking in the woods, dancing etc)

Teachers/adults and other staff = those who are in daily contact with the pupils. Use the country specific term that fits the best depending judging by the context of the question. Please, e-mail me if in doubt.

/ = and/or (I have tried to write and where there is only and. An or where there is only or)

School grounds = the area used by pupils during recess

School health services = any health services (i.e. vaccinations, consultation, information) that is being offered to children through their school by health personnel (i.e. doctors, nurses)

School premises = school indoor and outdoor where school has responsibility

3) Question specific clarifications

Q4 – by private we mean schools where the parents pay a fee to have their child going to school.

Q6 - the terminology for the type of school varies by country, but hopefully question 5 and 6 will give us a general impression about the size and type of school.

Q13 – if physical education is taught as double lessons this should count as 2, so that we can multiply with Q14 to find how much PE time is scheduled (we do not try to measure how much of that time the pupils are actually active, because headmasters won't know). I allow for different number of lessons in each grade level, but assume same length of the lessons.

Q15 – we aim to get a view of the most frequent opportunities to be active – not monthly or even less frequent events.

Q16 – here we aim to describe who is responsible for providing the foods/drinks consumed so we can target the intervention right.

Q17 – what are the costs related to foods/drinks available in school and who are paying these.

Q18 & Q19 – these ***practices*** should ideally be actively enforced practices as part of the routines.

There is a danger of pleasing bias with just yes/no, but we will try it like this for now and see if there will be meaningful distinctions between the schools/countries as I think not all practices can be assessed by a graded scale as they are now and also grading will require that headmasters need to spend more time on it. If we after the pre test think grading is important and feasible we can sort the remaining items into two and ask them to provide a grading form 1-3 for instance.

Not applicable would be if they don't have canteen etc or they don't have fund raising in school, teachers don't use rewards etc.

Q18 - I have not defined healthy foods/drinks or healthy eating as the important thing is whether the headmaster thinks they have the practices related to this.

Encourage = mainly verbal reminders

Q20 &Q21 – assessing who are involved in developing these practices and whether this has been on the agenda recently.

Q22 – again a difficult question because of the policy term is difficult to translate/define. I include it as I think it is important whether these practices are written down or just passed on orally, but then for now I use the policy terminology as this is what we often talk about and think are the important documents that every school should have. I do expect a lot of "yes, other" here, but the relevant documents will vary too much between countries to try to assess these within this project. I think finding that such policy documents don't exist is also important (and I could be wrong).

Section D – is very subjective, but will hopefully differentiate between the really healthy schools and the rest.

Promoting = making a substantial effort by one or several different strategies available

Q29 – assess whether the schools think that this is important (after using q23-q28 to make them think about this)

Q30 – assess whether the headmaster or the one who he/she delegated the completion of the questionnaire to are positively biased towards these topics

Q32/Q33 – new questions, related to WP6 about economic incentives or factors that can be used to promote healthy eating and physical activity. Thus a novelty and a core topic for ENERGY. If you have questions on the terminology - send them to both me and Jørgen.

Appendix II

Instruction pre test headmaster

[Place and date]

Dear Madame/Sir,

ENERGY Project

(E**uropea**N E**nergy balance** R**esearch to prevent excessive weight** G**ain among** Y**outh)**

We are very happy to hear that you agreed to participate in the pre test of the questionnaire that we have developed.

Fieldwork Protocol

Background and aim

In Europe, almost one in four children is affected by overweight or obesity. This prevalence is still increasing, reaching epidemic proportions. Overweight is caused by an imbalance of nutrition intake and physical activity, so called energy balance behaviours. The overall aim of the project is to obtain insight into the energy balance behaviours and their determinants in children aged 10-12 years olds, with the intention of developing an effective obesity prevention intervention.

A part of the aim of the project is to describe the variation in food and physical environments at schools across Europe. Therefore a school management questionnaire is developed and will be used in all eight countries during data collection in March, April and May 2010.

Reason for participation of you as a headmaster

Before the data collection starts, the school management questionnaire needs to be pre tested. We would kindly ask you to complete the questionnaire but also let us know if you have any problems with the understanding and interpretation of any of the questions. Completion of the questionnaire will take approximately 15 minutes of your time. Please put any of the following marks next to each question which, for any reason, is not clear to you:

U = unclear
W = weird question/formulation
M = answer is missing
O = other reason

After completion of the questionnaire you can add more notes on these questions about what you were thinking. These marks and the notes will be the starting point for a brief interview conducted by the researcher by phone before the 17th of February.

Voluntary

The participation of this study voluntary. You are free to withdraw from the study at any given time, without giving a reason. Choosing not to participate or withdrawing from this study will not have any consequences whatsoever.

Questionnaire and telephone contact

You will receive the questionnaire on [please enter date]. You can return the questionnaire by sending this back to the researcher in the postage paid envelope. To discuss the remarks that you have noted in the questionnaire and thus to discuss the questionnaire in more detail, we would like to contact you by telephone after completion of the questionnaire. This will take about 10 minutes of your time.

Contact and sending the questionnaires

If you have any questions or would like to have further information please do not hesitate to contact me by email or telephone (your email address / your telephone number).

Thank you in advance!

Kind regards,

RESEARCHER'S NAME

Appendix III

Checklist School management questionnaire

Instructions
The checklist needs to be completed by one researcher. Please make an appointment with the person who is representative for the school management for completing the questionnaire and a short face-to-face interview or, if not possible, a telephone interview.

Introduction telephone interview
First I would like to thank you for the willingness to participate in the pre test of the questionnaire. Thank you very much for taking the time to complete it and for having this telephone interview with me. The school management questionnaire is developed to assess the dietary and physical activity environment in schools across Europe. This will provide insight into how school environment varies across Europe and this can be seen in relation with dietary behaviours, physical activity and overweight. To develop a good and valuable instrument, the questionnaire has to be pre tested first on content and phrasing. This interview will approximately take 10 minutes of your time.

Items and remarks
[to be filled in according to the remarks on the completed questionnaire]
U = unclear
W = weird question/formulation
M = answer is missing
O = other reason

A. General questions

10. What do you think of the questionnaire? What was your first impression? *Try to get detailed information.*
11. What do you think of the length of the questionnaire? *When too long, from which part on or what topic.*
12. Were there any questions that you thought about but were not asked? If yes, on what topic?
13. What do you think of the sequence of questions and topics?
14. Were there any questions that were difficult to answer because you do not have information about this?
15. Were there any questions that were difficult to answer because an answer category was missing?
16. Did you find any errors in the questionnaire? If so, where?

B. Specific questions

1. Discuss the remarks that headmasters mentioned in the questionnaires. *Try to get detailed information so we have input for improvement of the questionnaires.*

If not discussed before:

1. Did you miss an instruction 'How to complete the questionnaire'?
    a. If no, why not?
    b. If yes, what kind of instruction would you find clear?
2. Were the short headings at each new section clear for you and correspond with the question topics afterwards ? If no, why not?

C Design of the questionnaire

1. What do you think about the design of the questionnaire? (clear/boring)
2. Is there anything you think that would make the questionnaire easier to complete?

Finish conversation

Do you have any additional comments or remarks about the questionnaire?

Thank you very much for providing us with your comments and making time available for this telephone interview. It has been very helpful.

Appendix G Children questionnaire protocol

1. Give a short introduction: ***"Today we will spend some time completing a questionnaire. As many as 8,000 school children all over Europe are involved in the study, so you can imagine how all of those are sitting in their classrooms at this very moment, doing the same thing as you". "So now please open the first page and I will read the introduction so that you understand why you are filling in this questionnaire today. "***

2. Read the introduction out loud to all children.

3. Explain the instructions on how to fill in the questionnaire.
   Emphasize to children the following:
   - ***"You are not allowed to make your own boxes. You can use a blue or black pencil. It is very important that the boxes need to be 'crossed' INSIDE the boxes and not be 'ticked'*** " *(Information to the researcher: the reason for this is to be able to scan the answers afterwards).*
   - ***"Some questions are more difficult than others to answer. If you get stuck on a question, please ask me. If it is still difficult, or none of the answers fit, please pick the closest one."*** Also emphasize that it is not a test so there are no right or wrong answers and the children can mark the answer that fits them or their situation best**.**
   - ***"If you wish to change an answer, leave the incorrect answer box marked with a 'x' and make the correct answer box completely black."*** Take a look at the example with the children at the instruction page. The researcher writes the example on the school board (draw the box with a cross inside and the boxes when an answer is wrong) so there will be no misunderstanding about this.

4. Explain the procedure that will be followed for the completion of the questionnaire.
   "The questionnaire has different sections. Before each section I will explain to you what it is about and THEN you will start answering the specific section. When you finish the section you wait until we move all together to the next section. This way everybody will answer the questions at the same time and you will be able to ask questions if you do not understand a question. Just raise your hand".
   "Some questions are about your parents or care givers. We hereby mean your father, mother or other person that is taking care of you at your home."

5. Start of the questionnaire.

- The researcher writes today's date (question A4) on the school board in front of the class so there will be no misunderstanding about this. ***"The first 7 questions are general "Questions about you". You can start answering"***

- Read the introduction part about soft drinks. Underline the fact that is NOT about diet drinks or fruit juices. It is important to explain the definition of soft drinks and fruit squash very well to the children. That syrups are included and, for example, Coca Cola light or Pepsi max are diet drinks/light drinks. ***"You can start answering the Soft drink section"***

"Look at question B2. Did you mark a box in every column? Question B2 and B3 look the same but B2 is about what you usually drink and question B3 is about what you drink yesterday. Do not get confused and read carefully what is asked.

From B6 on, children might do not know what to answer at the questions about their parents/care givers and friends. You can emphasize that they can try to give an answer. Also if the child does not have the knowledge than it is important that he/she will guess. This is also the case at some questions at the D, E and F section.

- Read the introduction part about fruit juices. ***"You can start answering the Fruit juices section"***

"Look at question C2. Did you mark a box in every column? Question C2 and C3 look the same but C2 is about what you usually drink and question C3 is about what you drink yesterday. Do not get confused and read carefully what is asked.

- Read the introduction part about breakfast. ***"You can start answering the Breakfast section".***
- Read the introduction part about physical activity. ***"Please answer the first questions about how you usually go to school".***
- ***""Please answer the question about what you normally do during breaks at school hours".***
- Read the introduction part about sport activities and add to the instruction that the children might sometimes not notice that they are sweating or feeling out of breath, like with gymnastics or swimming. ***"Question E9 asks about your favorite sport. What we mean by this is the sport you do the most often or regularly. This can also be either the sport that you like the most or not. For example, if you go swimming every week but you actually prefer to go ice skating, you do mention swimming as your favorite sport."*** Be aware that this also accounts for the second favorite sport, question E11.
- ***"Please answer the first questions about sport activities" "Look carefully at question E10 and E13 (E10 is about how many hours you do sport or physical activities during a USUAL WHOLE WEEK and E13 is only about yesterday."***
- ***"Now we will ask you general questions about physical activities and sport. What do you think about physical activities and sport".***
- ***"The next few questions are about "TV viewing.***
"Look carefully at the first questions F1 and F2. They are about the usual hours of television/DVD/video watching and playing games separately for weekdays and weekends. If you find it difficult to calculate the average, we can help you calculate this. You can think about how your days usually look like. When and how long do you watch television/DVD/video in the morning, afternoon and/or evening and think about how this differs among the weekdays (F1 first row) and also during the weekends (F1 second row). For example if you watch every evening after dinner till bedtime, dinner is finished at 19.00

and you go to bed at 20.30, you watch television/DVD/video about 1,5 hours/day on average." The researcher can do the same for playing computer games (question F2).

- ***"The next couple of questions are general questions about yourself." The questions at the G-section that are about you, you can fill in anonymous. Nobody will see what you have answered at these questions.***

6. *End of the questionnaire*

   *After finishing the questionnaire read out loud: "**Thank you for participating in this survey, you still have one more task: to bring the parent envelope home and give it to your parent or guardian for completion. Bring the completed questionnaire back to school as soon as possible."***

7. ***Greetings from the research group in country ...***

Appendix H Anthropometry datasheet (template recommended by VUmc, Not final)

Name Main Researcher	Name Assistant Researcher	Date	School number	Child number	Gender	Weight 1	Weight 2	Weight 3	Height 1	Height 2	Height 3	Waist 1	Waist 2	Waist 3

Appendix I

Work Package 7

Anthropometry Protocol

January 2010

A. General instructions

It is the measurer's responsibility to make the pupils feel comfortable about the measurements. The pupil can ask to stop the measuring at any time. Research assistants should wear casual clothes to comfort the children and have a neutral behavior (professional and serious). It is important to keep in mind that unexpected laughing can make the children feel uncomfortable.

Two people are needed to perform the measures; one provides instructions and is also taking the measurements and the other assists, registers the measures and checks that the measures are performed in the right way.

1. Dress code

The participant has to remove her/his shoes and wear t-shirts and shorts/short pants during the weight, height and waist circumference measurements. Researchers will have to bring to school spare shorts in case for those participants who forgot to bring/wear one. If it is possible, it is an option to schedule the measures on the day of gym lessons.

2. Readings

Two readings of each measurement (weight, height and waist) will be obtained to assure accuracy. If the two readings differ more than 1% then a third measurement will been taken. All three measurements will be recorded and the outlier will be excluded during the data cleaning process. The researcher is not allowed to provide the pupil with their weight and waist circumference measurements. Only the height measurement can be announced.

3. Materials required

- Balance (SECA 861)
- Portable stadiometer (SECA Leicester)
- Waist circumference measuring band (SECA 201)
- Manual for SECA 861 (resolve any possible problems)
- Screen(s) to create a private changing room (optional)
- Table(s) for: a) Researchers

b) Children to mention their name so the researcher knows which identification number belongs to the child or the child wears a sticker with their identification number on it.

- Laptop to entry data (optional)
- Clothes-pegs (keeping the shirts up when waist circumference is been measured)
- Spare batteries
- A set of spare shirts and shorts

Calibration

- **Daily:** Scales should be zeroed daily. Zeroing means that the scale reads zero when no weight is placed on it.
- **Monthly:** Scales and stadiometers should be calibrated monthly.
  Scales should be tested with standard weights.
  Stadiometers should be checked with a standard length rod.

B. Measurements

1. Weight

Equipment required: Weight will be measured with the electronic scale ***SECA 861***.
Spare batteries should be carried at all times.

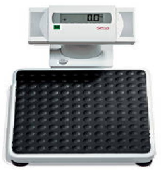

SECA 861

Method *(1 researcher and 1 research assistant is required)*

Researcher: The researcher pushes carefully on the balance and waits until the balance shows 00. For the measurement of weight, the pupil will be asked to stand next to the scale (Figure 1) and then step up sidewards onto the scale facing away from the readout (Figure 2). The responder will stand still over the center of the scale with body weight evenly distributed between both feet. The pupil's arms should be hanging freely by the

sides of the body, with palms facing the thighs. The responder should hold his/her head up, and face forward.

Research assistant: The research assistant registers the weight to the nearest 0.1 kg. without mentioning loudly and cleans the rubber surface in between the measurements.

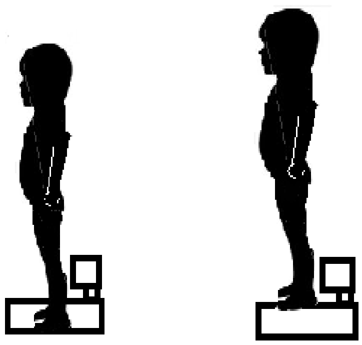

Figure 1. Figure 2.

Common errors

• The scale is not properly zeroed or balanced.

• Pupil is not properly centered on scale platform.

• Pupil is holding onto Assistant.

• Pupil is not remaining still on the scale.

Comments or instructions to pupils

- "I'm going to take three measurements – weight, height and waist circumference, in that order."
- "I'm then going to take those measurements again and, if any of the second measures are not close enough to the first ones, I'll measure you a third time."
- "Please take off your shoes and all your **shoes/long pants/sweater."**"Stand next to the scale."
- "Wait until it reads zero."
- "Step side wards onto the centre of the scale with your weight on both feet."
- "Look straight ahead."
- "Relax."
- "Thank you. You can step off now."

2. <u>Height</u>

Equipment required: Height will be measured with the portable stadiometer **SECA Leicester.**

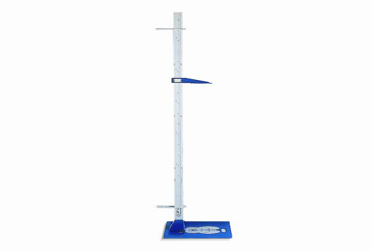

SECA Leicester

Method *(1 researcher and 1 research assistant is required)*

To measure standing height, hair ornaments must be removed and braids must be undone. The child stands on the stadiometer with bare feet (socks are allowed) placed slightly apart and the back of the head, shoulder blades, buttocks, calves, and heels touching the vertical board. Legs must be kept straight and the feet flat.

Researcher: The assistant researcher places the child's head in the **Frankfort plane position**; an imaginery horizontal line should be drawn from the ear canal to the lower edge of the eye socket running parallel to the baseboard. If their head is not aligned properly, the observer should ask them to raise or lower their chin until the head is in the Frankfort Plane. The headboard must be pulled down to rest firmly on top of the head and compress hair and the reading must be taken to the last completed 1 mm. The researcher checks if the head is in the Frankfort plane.

Research assistant: The research assistant checks the position of the lower part of the body and registers the height to the nearest 0.1 cm.

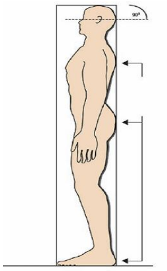

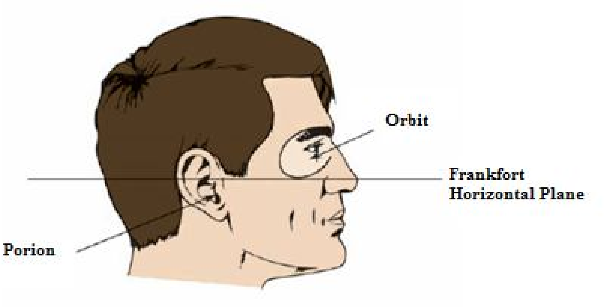

National Health and Nutrition Examination Survey. Anthropometry procedures manual, 2000

Common errors

- Feet are not flat on floor.
- Knees are bent.
- Head is not in proper position.
- Researcher focuses too much on the side of the child and does not check if the child stands in the middle of the measuring rod.

Comments or instructions to responders

- "Please stand backwards on the centre of the base with your back to the stadiometer. Put your feet together and move them back until your heels touch the back of the stadiometer. Stand up straight and look straight ahead."
- (If their head is not horizontal, say ...) "Please raise (or lower) your chin."
- "Take a deep breath and hold it."
- "That's fine, you can breathe normally now and step away from the stadiometer."

Additional point

- As the measuring rod can be unstable, assist or attend to participants in stepping on and balancing. Make an effort to ensure they are stable.
- If the subject has a hairstyle that stands well above the top of their head, bring the head plate down until it touches the head. If it is a hairstyle that can be altered (for example, a bun), ask them to change or undo it.
- In overweight, obese and older children, when the head is placed in proper position, according to the Frankfort Plane, there will be a space between the back of the child's head and the back of the measuring board.
- Do not judge the position of the child's head by looking at the top of the head, use the Frankfort Plane.
- If the measuring rod is not straight it has to be kept against the wall when measures are performed.

3. Waist circumference

Method required: The circumferences will be measured with the circumference measuring band ***SECA 201***. Be aware, the measurer has to start the measurement from the ZERO point as there are two sided numbers.

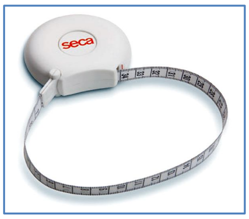

SECA 201

Method *(1 researcher and 1 research assistant is required)*

Researcher: The researcher instructs the child to stand erect with the abdomen relaxed, weight evenly balanced on both feet and his/her arms hanging by their side. The measurer faces the child and marks the lower costal (10th rib) border and the top of the iliac crest (Picture 1). The midaxillary line is marked between the costal border and the top of iliac crest (Picture 2) and an inelastic tape is used to measure the distance between them.

Research assistant: The research assistant marks the middle of this distance (Picture 3) and places the tape in a horizontal plane.

The measurement should be taken at the end of a normal expiration (so the child can not hold breath and tries to relax), without the tape compressing the skin. It is recorded to the nearest 0.1 cm by the research assistant (Picture 4).

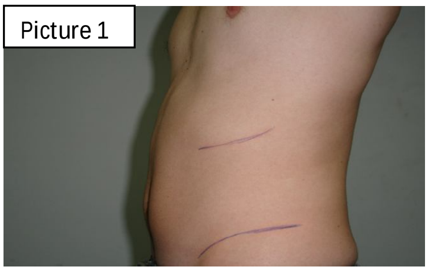

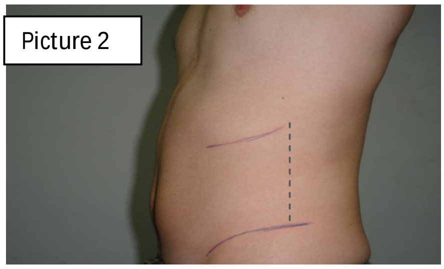

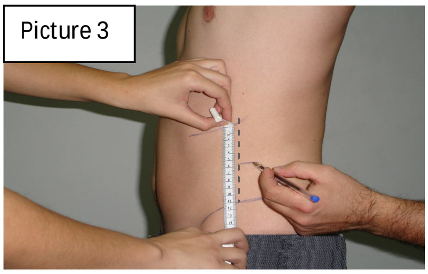

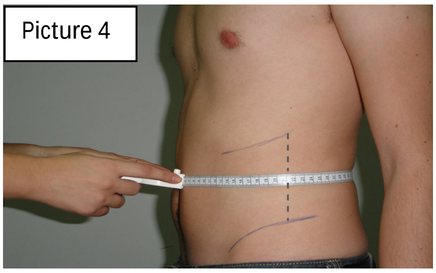

Common errors

- The measuring band is not horizontal.
- The measuring band is compressing the skin.
- The child is holding his/her breath.

Comments or instructions to responders

- If you have problems palpating the rib, ask the subject to breathe in very deeply. Locate the rib and as the subject breathes out, follow the rib as it moves down with your finger.
- The other option for locating the rib is to ask the subject to bend the upper body sideward.

Appendix J   Accelerometer protocol

LOGISTIC PLAN FOR accelerometer data collection

Methodology of school selection for WP7 ENERGY-accelerometer data

The schools for the accelerometer data collection will be selected from the list of randomly selected schools as sent by RESCON. We aimed to collect accelerometer data from at least 200 children per country from 4 schools (50 students per school). The selection of schools should be balanced across the 3 cities (selected from 3 tertiles), which were selected for the main survey. Distribution of the number of selected schools for accelerometer study should be proportional with the number of schools in the selected cities (thus more schools from larger cities).

One identical recruitment letter will be sent to all randomly selected schools explaining the possibility that some schoolchildren can be asked to wear accelerometers during one week. The selection of the four schools for accelerometer data collection will be based on the logistic planning of the survey data collection process.

Below we present a sample time schedule for accelerometer data collection. This sample plan is based on a total number of 240 accelerometers available (60 per country). One extra school (5th) is included in a sample time schedule taking into account potential difficulties to reach the necessary number of participants per school.

A Sample Time Schedule for Data Collection

MARCH 2010

Comment [V3]: To be adapted by VUmc.

Sun	Mon	Tue	Wed	Thu	Fri	Sat
	**1** CSA should be prepared for the 1st school	**2** Handing out CSAs in the 1st school (~50 students)	**3** 1st day of wearing CSA (1st school)	**4** 2nd day of wearing CSA (1st school)	**5** 3rd day of wearing CSA (1st school)	**6** 4th day of wearing CSA (1st school)
**7** 5th day of wearing CSA (1st school)	**8** Handing in CSAs back to school	**9** Collecting CSAs from school	**10** Preparing CSAs for the next school	**11** Preparing CSAs for the next school	**12** Handing out CSAs in the 2nd school (~50 students)	**13** 1st day of wearing CSA (2nd school)
**14** 2nd day of wearing CSA (2nd school)	**15** 3rd day of wearing CSA (2nd school)	**16** 4th day of wearing CSA (2nd school)	**17** 5th day of wearing CSA (2nd school)	**18** Handing in CSAs back to school	**19** Collecting CSAs from school	**20**
**21**	**22** Preparing CSAs for the next school	**23** Preparing CSAs for the next school	**24** Handing out CSAs in the 3rd school (~50 students)	**25** 1st day of wearing CSA (3rd school)	**26** 2nd day of wearing CSA (3rd school)	**27** 3rd day of wearing CSA (3rd school)
**28** 4th day of wearing CSA (3rd school)	**29** 5th day of wearing CSA (3rd school)	**30** Handing in CSAs back to school	**31** Collecting CSAs from school	**Notes:**		

APRIL 2010

Sun	Mon	Tue	Wed	Thu	Fri	Sat
				**1** Preparing CSAs for the next school	**2** Preparing CSAs for the next school	**3**
**4** EASTERN	**5** EASTERN	**6** Handing out CSAs in the 4th school (~50 students)	**7** 1st day of wearing CSA (4th school)	**8** 2nd day of wearing CSA (4th school)	**9** 3rd day of wearing CSA (4th school)	**10** 4th day of wearing CSA (4th school)
**11** 5th day of wearing CSA (4th school)	**12** Handing in CSAs back to school	**13** Collecting CSAs from school	**14** Preparing CSAs for the next school	**15** Preparing CSAs for the next school	**16** Handing out CSAs in the 5th school (~50 students)	**17** 1st day of wearing CSA (5th school)
**18** 2nd day of wearing CSA (5th school)	**19** 3rd day of wearing CSA (5th school)	**20** 4th day of wearing CSA (5th school)	**21** 5th day of wearing CSA (5th school)	**22** Handing in CSAs back to school	**23** Collecting CSAs from school	**24**
**25**	**26** Downloading Data	**27** Downloading & Combining Data	**28** Delivery of data files to VUMc	**29** Return of accelerometers*	**30**	**Notes:**

** Only applicable if accelerometers are borrowed from another country*

Distributing accelerometers between countries

Since the participating countries have different models of accelerometers, we propose to equally distribute the available accelerometers between the countries. For the present study only Actitrainers and GT1Ms will be used to provide similar, comparable results in the end. Total numbers can change due to the possibility that some of the devices are needed in other ongoing studies during March and April 2010. However it is expected to have at least 240 accelerometers in total. If Switzerland can provide the additional GT3Xs, accelerometers will be divided per country as 30 triaxis (Triaxis Actitrainer and GT3X) and 30 uniaxis (Uniaxis Actitrainer and GT1M) models. This will make sure that every country will be able to measure 50 students per school considering the possibility of damage or loss of accelerometers. You can see below the number of accelerometers available per country;

Netherlands - 75 Triaxis Actitrainers
10 Dual axis Actitrainers
Greece - 8 GT1M
Belgium - 128 GT1M, 46 MTI 7164's, 10 GT3x's.
Switzerland 108 CSA 7164 and 30 GT3X (optionally 270 GT1M and 15 additional GT3X)

Data collection at schools (March, April and May 2010)

Research assistants will be trained during the project meeting in Gent on the 5th of February and are responsible for the accelerometer data collection according to standardised protocols. Accelerometers should be initialized and prepared before handing out in schools. Information about accelerometer

use will be given to schoolchildren and at the end of the information session, accelerometers will be handed out. Additionally, children and parents will receive a brochure about accelerometer use and a diary. Teachers will also be informed about the procedures and asked to remind the children to wear the devices every day. Children will bring the device back to school and hand it to their teacher. Accelerometers will be collected from schools the day after. They will be prepared for the next school, i.e. charging, downloading data, initializing. In the end it is expected to have data from 800 children (200 per country).

Central data collection (End of May)

All data collected in the study will be transferred to VUmc, Netherlands. Data analyses will be performed by VUmc. The table below shows a time line which includes the activities and steps in the accelerometer study.

TIMELINE

	**Netherlands, Greece, Ghent, Switzerland**
**November 2009**	*School recruitment.<br>*Obtaining METC approval
**December 2009**	*School recruitment.
**January 2010**	*Translating a diary for accelerometer use in native language<br>*Translating a brochure for accelerometer use in native language<br>*School selection for accelerometer study.
**February 2010**	*Information letters to parents and informed consent letter.<br>*Sending back the list of recruited schools and appointments made with schools to ResCon.<br>*Training research assistants for accelerometer study<br>*Distributing devices between countries<br>*Preparing devices for the first school
**March April and May 2010**	*Data collection and sending the data files to the VUmc.<br>*Delivery of accelerometers to its original institute
