# Supplementary material for: EuropeaN Energy balance Research to prevent excessive weight Gain among Youth (ENERGY) project: Design and methodology of the ENERGY cross-sectional survey
Source: BMC Public Health. 2011 Jan 31;11:65. doi: 10.1186/1471-2458-11-65 (PMC3044658; doi:10.1186/1471-2458-11-65)
Supplement: Additional file 2 — codebook of ENERGY instruments. [file 1471-2458-11-65-S2.PDF]

Codeboek ENERGY CQ vragenlijst

Question	Variable name	Type	Value	Code	Compulsory
	VOLGNR	N3	001-998	001-998	Yes
Landcode	Land_c	N1	Belgium<br>Greece<br>Hungary<br>The Netherlands<br>Norway<br>Slovenia<br>Spain<br>Switzerland<br>Missing	1<br>2<br>3<br>4<br>5<br>6<br>7<br>8<br>-9	Yes
School number	School_c	N3	001-998<br>Missing	001-998<br>-9	Yes
Class number	Class_c	N1	5th grade<br>6th grade<br>Missing	5<br>6<br>-9	Yes
Code number	Student_c	N3	01-99<br>Missing	01-99<br>-9	Yes
A1. In what year were you born?	ca1	date	1997-2001<br>Missing	1997-2001<br>-9	No
A2. In what month is your birthday?	ca2	date	1<br>2<br>3<br>4<br>5<br>6<br>7<br>8<br>9<br>10<br>11<br>12<br>Missing	1<br>2<br>3<br>4<br>5<br>6<br>7<br>8<br>9<br>10<br>11<br>12<br>-9	No
A3. Are you a girl or a boy?	ca3	N1	Girl<br>Boy<br>Missing	0<br>1<br>-9	No
A4. What is today's date?					
day	ca4_d	Date	1-31<br>Missing	1-31<br>-9	No
month	ca4_m	Date	Januari<br>Februari<br>March<br>April<br>May<br>June<br>Juli<br>August<br>September<br>October<br>November<br>December<br>Missing	1<br>2<br>3<br>4<br>5<br>6<br>7<br>8<br>9<br>10<br>11<br>12<br>-9	No
year	ca4_y	Date	2010<br>Missing	2010<br>-9	No
A5. Which language do you most often speak at home?	ca5	N1	1<br>2<br>3	1<br>2<br>3	No

			4<br>5<br>Missing	4<br>5<br>-9	
A6. Which adults do you live with?					
Both my mother and my father all the time	ca6_1	N1	Selected<br>Not selected<br>Missing	1<br>0<br>-9	No
Only with my mother	ca6_2	N1	Selected<br>Not selected<br>Missing	1<br>0<br>-9	No
Only with my father	ca6_3	N1	Selected<br>Not selected<br>Missing	1<br>0<br>-9	No
With my mother and her new partner	ca6_4	N1	Selected<br>Not selected<br>Missing	1<br>0<br>-9	No
With my father and his new partner	ca6_5	N1	Selected<br>Not selected<br>Missing	1<br>0<br>-9	No
With my grandparents	ca6_6	N1	Selected<br>Not selected<br>Missing	1<br>0<br>-9	No
Other adults	ca6_7	N1	Selected<br>Not selected<br>Missing	1<br>0<br>-9	No
A7. Do you live with any brothers and/or sisters?					No
Yes, one or more older brother(s)	ca7_1	N1	Selected<br>Not selected<br>Missing	1<br>0<br>-9	No
Yes, one or more younger brother(s)	ca7_2	N1	Selected<br>Not selected<br>Missing	1<br>0<br>-9	No
Yes, one or more older sister(s)	ca7_3	N1	Selected<br>Not selected<br>Missing	1<br>0<br>-9	No
Yes, one or more younger sister(s)	ca7_4	N1	Selected<br>Not selected<br>Missing	1<br>0<br>-9	No
No, I do not live in the same house as my brother(s) or sister(s)	ca7_5	N1	Selected<br>Not selected<br>Missing	1<br>0<br>-9	No
I don’t have any brother(s) or sister(s)	ca7_6	N1	Selected<br>Not selected<br>Missing	1<br>0<br>-9	No
B1. How many times a week do you usually drink fizzy drinks and fruit squash?	cb1	N1	Never<br>Less than once a week<br>Once a week<br>2-4 days a week<br>5-6 days a week<br>Every day<br>Every day, more than once a day<br>Missing	0<br>0,5<br>1<br>3<br>5,5<br>7<br>8<br>-9	No
B2. On a day that you drink fizzy drinks and fruit squash, how many glasses, cans or bottles do you drink on such a day?					
a.Glasses or small	cb2_a	N1	None	0	No

bottles (250ml)			1 glass/small bottle<br>2 glasses/small bottles<br>3 glasses/small bottles<br>4 glasses/small bottles<br>5 or more glasses/small bottles<br>Missing	1<br>2<br>3<br>4<br>5<br>-9	
b.Cans (330ml)	cb2_b	N1	None<br>1 can<br>2 cans<br>3 cans<br>4 cans<br>5 or more cans<br>Missing	0<br>1<br>2<br>3<br>4<br>5<br>-9	No
c. Bottles (500ml)	cb2_c	N1	None<br>1 bottle<br>2 bottles<br>3 bottles<br>4 bottles<br>5 or more bottles<br>Missing	0<br>1<br>2<br>3<br>4<br>5<br>-9	No
B3. How many fizzy drinks and fruit squash did you drink yesterday?					
a. Glasses or small bottles (250ml)	cb3_a	N1	None<br>1 glass/small bottle<br>2 glasses/small bottles<br>3 glasses/small bottles<br>4 glasses/small bottles<br>5 or more glasses/small bottles<br>Missing	0<br>1<br>2<br>3<br>4<br>5<br>-9	No
b. Cans (330ml)	cb3_b	N1	None<br>1 can<br>2 cans<br>3 cans<br>4 cans<br>5 or more cans<br>Missing	0<br>1<br>2<br>3<br>4<br>5<br>-9	No
c. Bottles (500ml)	cb3_c	N1	None<br>1 bottle<br>2 bottles<br>3 bottles<br>4 bottles<br>5 or more bottles<br>Missing	0<br>1<br>2<br>3<br>4<br>5<br>-9	No
B4. I think that drinking fizzy drinks or fruit squash is…	cb4	N1	Very good<br>Good<br>Neither good nor bad<br>Bad<br>Very bad<br>Missing	1<br>2<br>3<br>4<br>5<br>-9	No
B5. I think drinking fizzy drinks or fruit squash will make me fat.	cb5	N1	I fully agree<br>I agree a bit<br>Neither agree nor disagree<br>I disagree a bit<br>I fully disagree<br>Missing	1<br>2<br>3<br>4<br>5<br>-9	No
B6. If I drink fizzy drinks or fruit squash, my parents/care givers thinks this is…	cb6	N1	Very good<br>Good<br>Neither good nor bad<br>Bad	1<br>2<br>3<br>4	No

			Very bad<br>Missing	5<br>-9	
B7. If I drink fizzy drinks or fruit squash, most of my friends think this is…	cb7	N1	Very good<br>Good<br>Neither good nor bad<br>Bad<br>Very bad<br>Missing	1<br>2<br>3<br>4<br>5<br>-9	No
B8. How often do your parents/care givers drink fizzy drinks or fruit squash?	cb8	N1	Always<br>Often<br>Sometimes<br>Not often<br>Never<br>Missing	1<br>2<br>3<br>4<br>5<br>-9	No
B9. How often do most of your friends drink fizzy drinks or fruit squash?	cb9	N1	Always<br>Often<br>Sometimes<br>Not often<br>Never<br>Missing	1<br>2<br>3<br>4<br>5<br>-9	No
B10. I like the taste of fizzy drinks or fruit squash.	cb10	N1	I fully agree<br>I agree a bit<br>Neither agree nor disagree<br>I disagree a bit<br>I fully disagree<br>Missing	1<br>2<br>3<br>4<br>5<br>-9	No
B11. Drinking fizzy drinks or fruit squash is something that I do without even really thinking about it.	cb11	N1	I fully agree<br>I agree a bit<br>Neither agree nor disagree<br>I disagree a bit<br>I fully disagree<br>Missing	1<br>2<br>3<br>4<br>5<br>-9	No
B12. I find drinking no fizzy drinks or fruit squash…	cb12	N1	Very easy<br>Easy<br>Neither easy nor difficult<br>Difficult<br>Very difficult<br>Missing	1<br>2<br>3<br>4<br>5<br>-9	No
B13. If I ask my parents/care givers for a fizzy drink or fruit squash, I get one.	cb13	N1	Always<br>Often<br>Sometimes<br>Not often<br>Never<br>Missing	1<br>2<br>3<br>4<br>5<br>-9	No
B14. I am allowed to take fizzy drinks or fruit squash, whenever I want.	cb14	N1	Always<br>Often<br>Sometimes<br>Not often<br>Never<br>Missing	1<br>2<br>3<br>4<br>5<br>-9	No
B15. Do your parents/care givers have rules about how many fizzy drinks or fruit squash you are allowed to drink?	cb15	N1	Yes<br>No<br>Missing	1<br>0<br>-9	No
B16. If you ask your parents/care givers to buy a certain brand of fizzy drink or fruit squash, will they do it?	cb16	N1	Always<br>Often<br>Sometimes<br>Not often<br>Never	1<br>2<br>3<br>4<br>5	No

			Missing	-9	
B17. Are there usually fizzy drinks or fruit squash at your home?	cb17	N1	Always<br>Often<br>Sometimes<br>Not often<br>Never<br>Missing	1<br>2<br>3<br>4<br>5<br>-9	No
B18. In which situations do you usually drink fizzy drinks or fruit squash?					
During the weekend	cb18_1	N1	Selected<br>Not selected<br>Missing	1<br>0<br>-9	No
Breakfast	cb18_2	N1	Selected<br>Not selected<br>Missing	1<br>0<br>-9	No
Lunch	cb18_3	N1	Selected<br>Not selected<br>Missing	1<br>0<br>-9	No
Dinner	cb18_4	N1	Selected<br>Not selected<br>Missing	1<br>0<br>-9	No
At school	cb18_5	N1	Selected<br>Not selected<br>Missing	1<br>0<br>-9	No
While watching television	cb18_6	N1	Selected<br>Not selected<br>Missing	1<br>0<br>-9	No
As a thirst quencher between meals	cb18_7	N1	Selected<br>Not selected<br>Missing	1<br>0<br>-9	No
During/after sports	cb18_8	N1	Selected<br>Not selected<br>Missing	1<br>0<br>-9	No
When I am with friends	cb18_9	N1	Selected<br>Not selected<br>Missing	1<br>0<br>-9	No
At birthdays/parties	cb18_10	N1	Selected<br>Not selected<br>Missing	1<br>0<br>-9	No
I never drink fizzy drinks or fruit squash	cb18_11	N1	Selected<br>Not selected<br>Missing	1<br>0<br>-9	No
B19. How often do you spend your own money on fizzy drinks or fruit squash?	cb19	N1	Always<br>Often<br>Sometimes<br>Not often<br>Never<br>Missing	1<br>2<br>3<br>4<br>5<br>-9	No
B20. If the price of fizzy drinks and fruit squash were doubled, I would buy less fizzy drinks or fruit squash from my own money.	cb20	N1	I fully agree<br>I agree a bit<br>Neither agree nor disagree<br>I disagree a bit<br>I fully disagree<br>I never buy fizzy drinks from my own money<br>Missing	1<br>2<br>3<br>4<br>5<br>6<br>-9	No
C1. How many times a week do you usually drink fruit juices?	cc1	N1	Never<br>Less than once a week<br>Once a week<br>2-4 days a week	0<br>0,5<br>1<br>3	No

			5-6 days a week<br>Every day<br>Every day, more than once a day<br>Missing	5,5<br>7<br>8<br>-9	
C2. On a day that you drink fruit juices, how many glasses or cartons do you drink on such a day?					
a. Glasses or small cartons (250ml)	cc2_a	N1	None<br>1 glass/small carton<br>2 glasses/small cartons<br>3 glasses/small cartons<br>4 glasses/small cartons<br>5 or more glasses/small cartons<br>Missing	0<br>1<br>2<br>3<br>4<br>5<br>-9	No
b. Regular cartons (330ml)	cc2_b	N1	None<br>1 carton<br>2 cartons<br>3 cartons<br>4 cartons<br>5 or more cartons<br>Missing	0<br>1<br>2<br>3<br>4<br>5<br>-9	No
C3. How many fruit juices did you drink yesterday?					
a. Glasses or small cartons (250ml)	cc3_a	N1	None<br>1 glass/small carton<br>2 glasses/small cartons<br>3 glasses/small cartons<br>4 glasses/small cartons<br>5 or more glasses/small cartons<br>Missing	0<br>1<br>2<br>3<br>4<br>5<br>-9	No
b. Regular cartons (330ml)	cc3_b	N1	None<br>1 carton<br>2 cartons<br>3 cartons<br>4 cartons<br>5 or more cartons<br>Missing	0<br>1<br>2<br>3<br>4<br>5<br>-9	No
C4. I think that drinking fruit juices is…	cc4	N1	Very good<br>Good<br>Neither good nor bad<br>Bad<br>Very bad<br>Missing	1<br>2<br>3<br>4<br>5<br>-9	No
C5. I think it is recommended for children my age ...	cc5	N1	Not to drink fruit juices at all<br>To drink fruit juices as much as you like<br>To drink not more than one glass a day<br>I don't know what is recommended<br>Missing	1<br>2<br>3<br>4<br>-9	No
C6. I think drinking fruit juices will make me fat.	cc6	N1	I fully agree<br>I agree a bit<br>Neither agree nor disagree<br>I disagree a bit<br>I fully disagree<br>Missing	1<br>2<br>3<br>4<br>5<br>-9	No
C7. I am allowed to take fruit juices, whenever I want.	cc7	N1	Always<br>Often<br>Sometimes<br>Not often<br>Never	1<br>2<br>3<br>4<br>5	No

			Missing	-9	
C8. Do your parents/care givers have rules about how many fruit juices you are allowed to drink?	cc8	N1	Yes<br>No<br>Missing	1<br>0<br>-9	No
C9. Are there usually fruit juices at your home?	cc9	N1	Always<br>Often<br>Sometimes<br>Not often<br>Never<br>Missing	1<br>2<br>3<br>4<br>5<br>-9	No
C10. In which situations are you most likely to drink fruit juices?					
During the weekend	cc10_1	N1	Selected<br>Not selected<br>Missing	1<br>0<br>-9	No
Breakfast	cc10_2	N1	Selected<br>Not selected<br>Missing	1<br>0<br>-9	No
Lunch	cc10_3	N1	Selected<br>Not selected<br>Missing	1<br>0<br>-9	No
Dinner	cc10_4	N1	Selected<br>Not selected<br>Missing	1<br>0<br>-9	No
At school	cc10_5	N1	Selected<br>Not selected<br>Missing	1<br>0<br>-9	No
While watching television	cc10_6	N1	Selected<br>Not selected<br>Missing	1<br>0<br>-9	No
As a thirst quencher between meals	cc10_7	N1	Selected<br>Not selected<br>Missing	1<br>0<br>-9	No
During/after sports	cc10_8	N1	Selected<br>Not selected<br>Missing	1<br>0<br>-9	No
When I am with friends	cc10_9	N1	Selected<br>Not selected<br>Missing	1<br>0<br>-9	No
At birthdays/parties	cc10_10	N1	Selected<br>Not selected<br>Missing	1<br>0<br>-9	No
I never drink fruit juices	cc10_11	N1	Selected<br>Not selected<br>Missing	1<br>0<br>-9	No
D1. From Monday to Friday during school weeks on how many days do you usually eat breakfast?	cd1	N1	I never eat breakfast on a schoolday<br>1 day<br>2 days<br>3 days<br>4 days<br>5 days<br>Missing	0<br>1<br>2<br>3<br>4<br>5<br>-9	No
D2. On how many days in the weekenddays (Saturday and Sunday) do you usually eat breakfast?	cd2		I never eat breakfast on weekendday<br>1 weekendday (Saterday OR Sunday)<br>2 weekenddays (Saterday AND Sunday)<br>Missing	0<br>1<br>2<br>-9	No
D3. What do you usually have for breakfast on school days?	cd3	N1	Just a drink<br>Just food<br>Drink with cold food	1<br>2<br>3	No

			Drink with hot food<br>Other<br>Missing	4<br>5<br>-9	
D4. What is the reason you usually skip breakfast?	cd4	N1	I never skip breakfast<br>I don't have enough time<br>I don't like breakfast products at home<br>I have never thought about it<br>I am not hungry in the morning<br>I just cannot eat early in the morning<br>Missing	1<br>2<br>3<br>4<br>5<br>6<br>-9	No
D5. Did you eat breakfast yesterday?	cd5	N1	Yes<br>No<br>Missing	1<br>0<br>-9	No
D6. Did you eat lunch yesterday?	cd6	N1	Yes<br>No<br>Missing	1<br>0<br>-9	No
D7. Did you eat dinner yesterday?	cd7	N1	Yes<br>No<br>Missing	1<br>0<br>-9	No
D8. Did you eat anything between meals yesterday?	cd8	N1	Yes<br>No<br>Missing	1<br>0<br>-9	No
D9. I think eating breakfast is…	cd9	N1	Very good<br>Good<br>Neither good nor bad<br>Bad<br>Very bad<br>Missing	1<br>2<br>3<br>4<br>5<br>-9	No
D10. I think it is recommended for children my age ...	cd10	N1	To skip breakfast<br>Eat breakfast if you feel like it<br>Eat breakfast on schooldays<br>Eat breakfast every day<br>I don't know what is recommended<br>Missing	1<br>2<br>3<br>4<br>5<br>-9	No
D11. I think NOT eating breakfast will make me fat.	cd11	N1	I fully agree<br>I agree a bit<br>Neither agree nor disagree<br>I disagree a bit<br>I fully disagree<br>Missing	1<br>2<br>3<br>4<br>5<br>-9	No
D12. I think eating breakfast will make me fat.	cd12	N1	I fully agree<br>I agree a bit<br>Neither agree nor disagree<br>I disagree a bit<br>I fully disagree<br>Missing	1<br>2<br>3<br>4<br>5<br>-9	No
D13. If I eat breakfast, my parents/care givers think this is…	cd13	N1	Very good<br>Good<br>Neither good nor bad<br>Bad<br>Very bad<br>Missing	1<br>2<br>3<br>4<br>5<br>-9	No
D14. If I eat breakfast, most of my friends think this is…	cd14	N1	Very good<br>Good<br>Neither good nor bad<br>Bad<br>Very bad<br>Missing	1<br>2<br>3<br>4<br>5<br>-9	No
D15. How often do your parents/care givers eat	cd15	N1	Always<br>Often	1<br>2	No

breakfast?			Sometimes<br>Not often<br>Never<br>Missing	3<br>4<br>5<br>-9	
D16. How often do most of your friends eat breakfast?	cd16	N1	Always<br>Often<br>Sometimes<br>Not often<br>Never<br>Missing	1<br>2<br>3<br>4<br>5<br>-9	No
D17. I like eating breakfast	cd17	N1	I fully agree<br>I agree a bit<br>Neither agree nor disagree<br>I disagree a bit<br>I fully disagree<br>Missing	1<br>2<br>3<br>4<br>5<br>-9	No
D18. Eating breakfast is something that I do without even really thinking about.	cd18	N1	I fully agree<br>I agree a bit<br>Neither agree nor disagree<br>I disagree a bit<br>I fully disagree<br>Missing	1<br>2<br>3<br>4<br>5<br>-9	No
D19. I find eating breakfast everyday…	cd19	N1	Very easy<br>Easy<br>Neither easy nor difficult<br>Difficult<br>Very difficult<br>Missing	1<br>2<br>3<br>4<br>5<br>-9	No
D20. My parents/care givers encourage me to have breakfast.	cd20	N1	I fully agree<br>I agree a bit<br>Neither agree nor disagree<br>I disagree a bit<br>I fully disagree<br>Missing	1<br>2<br>3<br>4<br>5<br>-9	No
D21. Do your parents/care givers have rules about whether you should eat breakfast?	cd21	N1	Yes<br>No<br>Missing	1<br>0<br>-9	No
D22. If you ask your parents/care givers to buy a certain brand of food or drink for breakfast, will they do it?	cd22	N1	Always<br>Often<br>Sometimes<br>Not often<br>Never<br>Missing	1<br>2<br>3<br>4<br>5<br>-9	No
D23. Are there usually breakfast products (milk, cereals, bread etc) at your home?	cd23	N1	Always<br>Often<br>Sometimes<br>Not often<br>Never<br>Missing	1<br>2<br>3<br>4<br>5<br>-9	No
D24. How often do you eat breakfast with your parents/ care givers?	cd24	N1	Never<br>Less than once a week<br>Once a week<br>2-4 days a week<br>5-6 days a week<br>Every day<br>Missing	0<br>0,5<br>1<br>3<br>5,5<br>7<br>-9	No
D25. In which situations do you usually eat your breakfast?					
At a set table at home	cd25_1	N1	Selected<br>Not selected	1<br>0	No

			Missing	-9	
In bed	cd25_2	N1	Selected<br>Not selected<br>Missing	1<br>0<br>-9	No
While watching television	cd25_3	N1	Selected<br>Not selected<br>Missing	1<br>0<br>-9	No
On my way to school	cd25_4	N1	Selected<br>Not selected<br>Missing	1<br>0<br>-9	No
At school before the class starts	cd25_5	N1	Selected<br>Not selected<br>Missing	1<br>0<br>-9	No
I never eat breakfast	cd25_6	N1	Selected<br>Not selected<br>Missing	1<br>0<br>-9	No
E1. How many days do you usually bike to school?	ce1	N1	I never bike to school<br>1 day per week<br>2 days per week<br>3 days per week<br>4 days per week<br>5 days per week<br>Missing	0<br>1<br>2<br>3<br>4<br>5<br>-9	No
E2. If you BIKE to school, how long does it take you to bike to school?	ce2	N1	I never bike to school<br>1-5 minutes<br>6-10 minutes<br>11-15 minutes<br>More than 15 minutes<br>Missing	0<br>1<br>2<br>3<br>4<br>-9	No
E3. How many days do you usually walk to school?	ce3	N1	I never walk to school<br>1 day per week<br>2 days per week<br>3 days per week<br>4 days per week<br>5 days per week<br>Missing	0<br>1<br>2<br>3<br>4<br>5<br>-9	No
E4. If you WALK to school, how long does it take you to bike to school?	ce4	N1	I never walk to school<br>1-5 minutes<br>6-10 minutes<br>11-15 minutes<br>More than 15 minutes<br>Missing	0<br>1<br>2<br>3<br>4<br>-9	No
E5. How many days do you usually travel by car to school?	ce5	N1	I never travel by car to school<br>1 day per week<br>2 days per week<br>3 days per week<br>4 days per week<br>5 days per week<br>Missing	0<br>1<br>2<br>3<br>4<br>5<br>-9	No
E6. How many days do you usually travel by public transport (bus, schoolbus, tram, metro) to school)?	ce6	N1	I never travel by public transport to school<br>1 day per week<br>2 days per week<br>3 days per week<br>4 days per week<br>5 days per week<br>Missing	0<br>1<br>2<br>3<br>4<br>5<br>-9	No
E7. How did you go to school today?					
By bike	ce7_1	N1	Selected<br>Not selected	1<br>0	No

			Missing	-9	
Walking	ce7_2	N1	Selected<br>Not selected<br>Missing	1<br>0<br>-9	No
By car	ce7_3	N1	Selected<br>Not selected<br>Missing	1<br>0<br>-9	No
Public transport	ce7_4	N1	Selected<br>Not selected<br>Missing	1<br>0<br>-9	No
E8. What do you usually do during breaks at school?	ce8	N1	I usually spend time sitting<br>I usually spend time walking around<br>I usually spend time with physical activity<br>Missing	1<br>2<br>3<br>-9	No
E9. My FAVORITE sport is	ce9	A50	Not available (if ce9_1 = 1)<br>Missing	-8<br>-9	No
E9. I do not participate in any sports acivities	ce9_1	N1	Selected<br>Not selected<br>Missing	1<br>0<br>-9	No
E10. In a TOTAL WEEK how many hours do you do this sport?	ce10	N1	30 minutes/week<br>1 hour/week<br>1,5 hour/week<br>2 hours/week<br>2,5 hours/week<br>3 hours/week<br>3,5 hours/week<br>4 hours/week<br>4,5 hours/week<br>5 hours/week<br>Not available (if ce9_1 = 1)<br>Missing	0,5<br>1<br>1,5<br>2<br>2,5<br>3<br>3,5<br>4<br>4,5<br>5<br>-8<br>-9	No
E11. My second FAVORITE sport is	ce11	A50	Not available (if ce9_1 or ce11_1 = 1)<br>Missing	-8<br>-9	No
E11. I do not have a second sport	ce11_1	N1	Selected<br>Not selected<br>Not available (if ce9_1 = 1)<br>Missing	1<br>0<br>-8<br>-9	No
E12. In a TOTAL WEEK how many hours do you do this sport?	ce12	N1	30 minutes/week<br>1 hour/week<br>1,5 hour/week<br>2 hours/week<br>2,5 hours/week<br>3 hours/week<br>3,5 hours/week<br>4 hours/week<br>4,5 hours/week<br>5 hours/week<br>Not available (if ce9_1 or ce11_1 = 1)<br>Missing	0,5<br>1<br>1,5<br>2<br>2,5<br>3<br>3,5<br>4<br>4,5<br>5<br>-8<br>-9	No
E13. How many hours of sports did you do yesterday?	ce13	N1	30 minutes/week<br>1 hour/week<br>1,5 hour/week<br>2 hours/week<br>2,5 hours/week<br>3 hours/week<br>3,5 hours/week<br>4 hours/week<br>4,5 hours/week<br>5 hours/week<br>Not available (if ce9_1 or ce11_1 = 1)	0,5<br>1<br>1,5<br>2<br>2,5<br>3<br>3,5<br>4<br>4,5<br>5<br>-8	No

			Missing	-9	
E14. I think that physical activity/sports is…	ce14	N1	Very good<br>Good<br>Neither good nor bad<br>Bad<br>Very bad<br>Missing	1<br>2<br>3<br>4<br>5<br>-9	No
E15. I think it is recommended for children my age ...	ce15	N1	To be active once a week<br>To be active some days a week<br>To be active every day for 30 minutes<br>To be active every day for 1 hour<br>To be active every day for 2 hours<br>To be active every day for 3 or 4 hours<br>I don’t know what is recommended<br>Missing	1<br>2<br>3<br>4<br>5<br>6<br>7<br>-9	No
E16. I think NOT doing physical activity/sports will make me fat.	ce16	N1	I fully agree<br>I agree a bit<br>Neither agree nor disagree<br>I disagree a bit<br>I fully disagree<br>Missing	1<br>2<br>3<br>4<br>5<br>-9	No
E17. If I do physical activity/sports, my parents/care givers think this is…	ce17	N1	Very good<br>Good<br>Neither good nor bad<br>Bad<br>Very bad<br>Missing	1<br>2<br>3<br>4<br>5<br>-9	No
E18. If I do physical activity/sports, most of my friends think this is…	ce18	N1	Very good<br>Good<br>Neither good nor bad<br>Bad<br>Very bad<br>Missing	1<br>2<br>3<br>4<br>5<br>-9	No
E19. How often do your parents/care givers do physical activity/sports?	ce19	N1	Always<br>Often<br>Sometimes<br>Not often<br>Never<br>Missing	1<br>2<br>3<br>4<br>5<br>-9	No
E20. How often do most of your friends do physical activity/sports?	ce20	N1	Always<br>Often<br>Sometimes<br>Not often<br>Never<br>Missing	1<br>2<br>3<br>4<br>5<br>-9	No
E21. I like doing physical activity/sports.	ce21	N1	I fully agree<br>I agree a bit<br>Neither agree nor disagree<br>I disagree a bit<br>I fully disagree<br>Missing	1<br>2<br>3<br>4<br>5<br>-9	No
E22. Doing physical activity/sports is something that I do without even really thinking about.	ce22	N1	I fully agree<br>I agree a bit<br>Neither agree nor disagree<br>I disagree a bit<br>I fully disagree<br>Missing	1<br>2<br>3<br>4<br>5<br>-9	No
E23. I find doing physical activity/sports for 1 hour everyday…	ce23	N1	Very easy<br>Easy<br>Neither easy nor difficult	1<br>2<br>3	No

			Difficult<br>Very difficult<br>Missing	4<br>5<br>-9	
E24. My parents/care givers encourage me to do physical activity/sports.	ce24	N1	I fully agree<br>I agree a bit<br>Neither agree nor disagree<br>I disagree a bit<br>I fully disagree<br>Missing	1<br>2<br>3<br>4<br>5<br>-9	No
E25. My parents/care givers help me if I need something for my sports.	ce25	N1	I fully agree<br>I agree a bit<br>Neither agree nor disagree<br>I disagree a bit<br>I fully disagree<br>Missing	1<br>2<br>3<br>4<br>5<br>-9	No
E26. Do your parents/care givers have rules about whether you should be physically active/do sports?	ce26	N1	Yes<br>No<br>Missing	1<br>0<br>-9	No
E27. Do your parents/care givers allow you to take part in physical activity/do sports?	ce27	N1	Yes<br>No<br>Missing	1<br>0<br>-9	No
E28. If you indicate that you like a certain physical activity /sports will your parents/care givers allow you to do it ?	ce28	N1	Always<br>Often<br>Sometimes<br>Not often<br>Never<br>Missing	1<br>2<br>3<br>4<br>5<br>-9	No
E29. Do you have the following things at home that you can use for physical activities/sports?					
Bike	ce29_1	N1	Yes<br>No<br>Missing	1<br>0<br>-9	No
Tennis and/or badminton racket	ce29_2	N1	Yes<br>No<br>Missing	1<br>0<br>-9	No
Ball (basketball, volleyball, football ect)	ce29_3	N1	Yes<br>No<br>Missing	1<br>0<br>-9	No
Sporting shoes	ce29_4	N1	Yes<br>No<br>Missing	1<br>0<br>-9	No
Skipping rope	ce29_5	N1	Yes<br>No<br>Missing	1<br>0<br>-9	No
Skates	ce29_6	N1	Yes<br>No<br>Missing	1<br>0<br>-9	No
Ski’s	ce29_7	N1	Yes<br>No<br>Missing	1<br>0<br>-9	No
Skate board	ce29_8	N1	Yes<br>No<br>Missing	1<br>0<br>-9	No
E30. How often do you take part in physical activity/ do sports with your parents care givers?	ce30	N1	Never<br>Less than once a week<br>Once a week<br>2-4 days a week<br>5-6 days a week	0<br>0,5<br>1<br>3<br>5,5	No

			Every day<br>Missing	7<br>-9	
F1. About how many hours a day do you usually watch television in your free time?		N1			
Average of all weekdays	cf1_a	N1	Non at all<br>30 minutes/week<br>1 hour/week<br>1,5 hour/week<br>2 hours/week<br>2,5 hours/week<br>3 hours/week<br>3,5 hours/week<br>4 or more hours/week<br>Missing	0<br>0,5<br>1<br>1,5<br>2<br>2,5<br>3<br>3,5<br>4<br>-9	No
Average of all weekenddays	cf1_b	N1	Non at all<br>30 minutes/week<br>1 hour/week<br>1,5 hour/week<br>2 hours/week<br>2,5 hours/week<br>3 hours/week<br>3,5 hours/week<br>4 or more hours/week<br>Missing	0<br>0,5<br>1<br>1,5<br>2<br>2,5<br>3<br>3,5<br>4<br>-9	No
F2. About how many hours a day do you usually play games ona a computer, or use your computer for leisure activity in your free time?		N1			
Average of all weekdays	cf2_a	N1	Non at all<br>30 minutes/week<br>1 hour/week<br>1,5 hour/week<br>2 hours/week<br>2,5 hours/week<br>3 hours/week<br>3,5 hours/week<br>4 or more hours/week<br>Missing	0<br>0,5<br>1<br>1,5<br>2<br>2,5<br>3<br>3,5<br>4<br>-9	No
Average of all weekenddays	cf2_b	N1	Non at all<br>30 minutes/week<br>1 hour/week<br>1,5 hour/week<br>2 hours/week<br>2,5 hours/week<br>3 hours/week<br>3,5 hours/week<br>4 or more hours/week<br>Missing	0<br>0,5<br>1<br>1,5<br>2<br>2,5<br>3<br>3,5<br>4<br>-9	No
F3. About how many hours did you watch television yesterday?	cf3	N1	Non at all<br>30 minutes/week<br>1 hour/week<br>1,5 hour/week<br>2 hours/week<br>2,5 hours/week<br>3 hours/week<br>3,5 hours/week<br>4 or more hours/week<br>Missing	0<br>0,5<br>1<br>1,5<br>2<br>2,5<br>3<br>3,5<br>4<br>-9	No

F4. About how many hours a day did you play games on a computer, or use your computer for leisure activity yesterday?	cf4	N1	Non at all<br>30 minutes/week<br>1 hour/week<br>1,5 hour/week<br>2 hours/week<br>2,5 hours/week<br>3 hours/week<br>3,5 hours/week<br>4 or more hours/week<br>Missing	0<br>0,5<br>1<br>1,5<br>2<br>2,5<br>3<br>3,5<br>4<br>-9	No
F5. I think that physical activity/sports is	cf5	N1	Very good<br>Good<br>Neither good nor bad<br>Bad<br>Very bad<br>Missing	1<br>2<br>3<br>4<br>5<br>-9	No
F6. I think it is recommended for children my age ...	cf6	N1	Not to watch tv at all<br>To watch tv not more than few times per week<br>To watch tv for less than 1 hour per day<br>To watch tv for less than 2 hours per day<br>To watch tv for more than 2 hours per day<br>To watch television as often as you like<br>I don’t know what is recommended<br>Missing	1<br>2<br>3<br>4<br>5<br>6<br>7<br>-9	No
F7. I think watching too much television will make me fat.	cf7	N1	I fully agree<br>I agree a bit<br>Neither agree nor disagree<br>I disagree a bit<br>I fully disagree<br>Missing	1<br>2<br>3<br>4<br>5<br>-9	No
F8. If I watch television, my parents/care givers think this is…	cf8	N1	Very good<br>Good<br>Neither good nor bad<br>Bad<br>Very bad<br>Missing	1<br>2<br>3<br>4<br>5<br>-9	No
F9. If I watch television, most of my friends think this is…	cf9	N1	Very good<br>Good<br>Neither good nor bad<br>Bad<br>Very bad<br>Missing	1<br>2<br>3<br>4<br>5<br>-9	No
F10. How often do your parents/care givers watch television?	cf10	N1	Always<br>Often<br>Sometimes<br>Not often<br>Never<br>Missing	1<br>2<br>3<br>4<br>5<br>-9	No
F11. How often do most of your friends watch television?	cf11	N1	Always<br>Often<br>Sometimes<br>Not often<br>Never<br>Missing	1<br>2<br>3<br>4<br>5<br>-9	No
F12. I like watching television.	cf12	N1	I fully agree<br>I agree a bit<br>Neither agree nor disagree<br>I disagree a bit<br>I fully disagree<br>Missing	1<br>2<br>3<br>4<br>5<br>-9	No

F13. Watching television is something that I do without even really thinking about.	cf13	N1	I fully agree<br>I agree a bit<br>Neither agree nor disagree<br>I disagree a bit<br>I fully disagree<br>Missing	1<br>2<br>3<br>4<br>5<br>-9	No
F14. I find NOT watching television …	cf14	N1	Very easy<br>Easy<br>Neither easy nor difficult<br>Difficult<br>Very difficult<br>Missing	1<br>2<br>3<br>4<br>5<br>-9	No
F15. My parents/caregivers allow me to watch television, whenever I want.	cf15	N1	I fully agree<br>I agree a bit<br>Neither agree nor disagree<br>I disagree a bit<br>I fully disagree<br>Missing	1<br>2<br>3<br>4<br>5<br>-9	No
F16. If I ask my parents/care givers to watch television, I can do so	cf16	N1	Always<br>Often<br>Sometimes<br>Not often<br>Never<br>Missing	1<br>2<br>3<br>4<br>5<br>-9	No
F17. Do your parents/care givers have rules about how many hours per day you are allowed to watch television?	cf17	N1	Yes<br>No<br>Missing	1<br>0<br>-9	No
F18. Do you have a television in your bedroom?	cf18	N1	Yes<br>No<br>Missing	1<br>0<br>-9	No
F19. How often do you watch television with your parents care givers?	cf19	N1	Never<br>Less than once a week<br>Once a week<br>2-4 days a week<br>5-6 days a week<br>Every day<br>Every day, more than once a day<br>Missing	0<br>0,5<br>1<br>3<br>5,5<br>7<br>8<br>-9	No
F20. How often do you watch television during meals?		N1			
Breakfast	Cf20_break	N1	Always<br>Often<br>Sometimes<br>Not often<br>Never<br>Missing	1<br>2<br>3<br>4<br>5<br>-9	No
Lunch	Cf20_lunch	N1	Always<br>Often<br>Sometimes<br>Not often<br>Never<br>Missing	1<br>2<br>3<br>4<br>5<br>-9	No
Dinner	cf20_dinner	N1	Always<br>Often<br>Sometimes<br>Not often<br>Never<br>Missing	1<br>2<br>3<br>4<br>5<br>-9	No
G1. Do you think you are too	cg1	N1	I am much too thin	1	No

thin or too fat?			I am a bit too thin<br>I am not too thin nor too fat<br>I am a bit too fat<br>I am much too fat<br>Missing	2<br>3<br>4<br>5<br>-9	
G2. How often have you tried to get slimmer/thinner during the last year?	cg2	N1	None<br>1-4 times<br>5-10 times<br>More than 10 times<br>I try to slim all the time<br>Missing	0<br>1<br>2<br>3<br>4<br>-9	No
G3. Do you try to get slimmer/thinner right now?	cg3	N1	Yes<br>No<br>Missing	1<br>0<br>-9	No

Codeboek ENERGY PQ vragenlijst

Question	Variable name	Type	Value	Code	Compulsory
	VOLGNR	N3	001-998	001-998	Yes
Landcode	Land_p	N1	Belgium<br>Greece<br>Hungary<br>The Netherlands<br>Norway<br>Slovenia<br>Spain<br>Switzerland<br>Missing	1<br>2<br>3<br>4<br>5<br>6<br>7<br>8<br>-9	Yes
School number	School_p	N3	001-998<br>Missing	001-998<br>-9	Yes
Class number	Class_p	N1	05<br>06<br>Missing	5<br>6<br>-9	Yes
Code number	Student_p	N3	01-99<br>Missing	01-99<br>-9	Yes
A1. In which month/year is your child born? month	pa1_m	date	1<br>2<br>3<br>4<br>5<br>6<br>7<br>8<br>9<br>10<br>11<br>12<br>Missing	1<br>2<br>3<br>4<br>5<br>6<br>7<br>8<br>9<br>10<br>11<br>12<br>-9	No
A1. In which month/year is your child born? year	pa1_y	date	1997-2001<br>Missing	1997-2001<br>-9	No
A2. What is today's date? month	pa2_m	date	1<br>2<br>3<br>4<br>5<br>6<br>7<br>8<br>9<br>10<br>11<br>12<br>Missing	1<br>2<br>3<br>4<br>5<br>6<br>7<br>8<br>9<br>10<br>11<br>12<br>-9	No
A2. What is today's date? year	pa2_y	date	2010<br>Missing	2010<br>-9	No
A3. This questionnaire is filled in by...	pa3	N1	The mother<br>The stepmother<br>The father<br>The stepfather<br>Other<br>Missing	1<br>2<br>3<br>4<br>5<br>-9	No
A4. What is you age?	pa4	N2	01-99<br>Missing	01-99<br>-9	No
A5. What is your marital status?	pa5	N1	Single	1	No

			Married<br>Living with my partner, but not married<br>Separated<br>Divorced<br>Other<br>Missing	2<br>3<br>4<br>5<br>6<br>-9	
A6. What is your weight?	pa6	N3	001-998<br>Missing	001-998<br>-9	No
A7. What is your height?	pa7	N3	001-998<br>Missing	001-998<br>-9	No
A8. Where the biological parents of the child born in ....?	pa8	N1	Yes<br>No, only one parent<br>No, none of the parents<br>Missing	1<br>2<br>3<br>-9	No
A9. How many years of school education did you/ your partner complete? Me	pa9_1	N1	Less than 7 years<br>7-9 years<br>10-11 years<br>12-13 years<br>14 years or more<br>Missing	1<br>2<br>3<br>4<br>5<br>-9	No
A9. How many years of school education did you/ your partner complete? Spouse/Partner	pa9_2	N1	Less than 7 years<br>7-9 years<br>10-11 years<br>12-13 years<br>14 years or more<br>Missing	1<br>2<br>3<br>4<br>5<br>-9	No
A9. I do not have a spouse/ partner	pa9_3	N1	Selected<br>Not selected<br>Missing	1<br>0<br>-9	No
A10. What is your main occupation? Me	pa10_1	N1	Employee in public secotr<br>Employee in private sector<br>Self-employed<br>No paid job<br>Missing	1<br>2<br>3<br>4<br>-9	No
A10. What is your main occupation? Spouse/Partner	pa10_2	N1	Employee in public secotr<br>Employee in private sector<br>Self-employed<br>No paid job<br>Missing	1<br>2<br>3<br>4<br>-9	No
A 10. I do not have a spouse/ partner	pa10_3	N1	Selected<br>Not selected<br>Missing	1<br>0<br>-9	No
B1. How many times a week do you usually drink soft drinks?	pb1	N1	Never<br>Less than once a week<br>Once a week<br>2-4 days a week<br>5-6 days a week<br>Every day<br>Every day, more than once a day<br>Missing	0<br>0,5<br>1<br>3<br>5,5<br>7<br>8<br>-9	No
B2. On a day that you drink soft drinks, how many glasses, cans or bottles do you drink on such a day?					
a.Glasses or small bottles (250ml)	pb2_a	N1	None<br>1 glass/small bottle<br>2 glasses/small bottles<br>3 glasses/small bottles<br>4 glasses/small bottles<br>5 or more glasses/small bottles	0<br>1<br>2<br>3<br>4<br>5	No

			Not available (if pb1=0)<br>Missing	-8<br>-9	
b.Cans (330ml)	pb2_b	N1	None<br>1 can<br>2 cans<br>3 cans<br>4 cans<br>5 or more cans<br>Not available (if pb1=0)<br>Missing	0<br>1<br>2<br>3<br>4<br>5<br>-8<br>-9	No
c.Bottles (500ml)	pb2_c	N1	None<br>1 bottle<br>2 bottles<br>3 bottles<br>4 bottles<br>5 or more bottles<br>Not available (if pb1=0)<br>Missing	0<br>1<br>2<br>3<br>4<br>5<br>-8<br>-9	No
B3. Drinking soft drinks is something that I do without even really thinking about it.	pb3	N1	I fully agree<br>I agree a bit<br>Neither agree nor disagree<br>I disagree a bit<br>I fully disagree<br>Not available (if pb1=0)<br>Missing	1<br>2<br>3<br>4<br>5<br>-8<br>-9	No
B4. There are soft drinks available at home for my child	pb4	N1	Always<br>Often<br>Sometimes<br>Not often<br>Never<br>Missing	1<br>2<br>3<br>4<br>5<br>-9	No
B5. I pay attention to the amount of soft drinks that my child drinks	pb5	N1	Always<br>Often<br>Sometimes<br>Not often<br>Never<br>Missing	1<br>2<br>3<br>4<br>5<br>-9	No
B6. If my child asks for soft drinks, I will give it to him/her	pb6	N1	Always<br>Often<br>Sometimes<br>Not often<br>Never<br>Missing	1<br>2<br>3<br>4<br>5<br>-9	No
B7. My childis allowed to take soft drinks, whenever (s)he wants	pb7	N1	Always<br>Often<br>Sometimes<br>Not often<br>Never<br>Missing	1<br>2<br>3<br>4<br>5<br>-9	No
B8. I negotiate with my child how much soft drinks (s)he is allowed to drink	pb8	N1	Always<br>Often<br>Sometimes<br>Not often<br>Never<br>Missing	1<br>2<br>3<br>4<br>5<br>-9	No
B9. How often do you tell your child that soft drinks are not good for him/her	pb9	N1	Always<br>Often<br>Sometimes<br>Not often<br>Never	1<br>2<br>3<br>4<br>5	No

			Missing	-9	
B10.How often do you tell your child that soft drinks can make him/her fat?	pb10	N1	Always<br>Often<br>Sometimes<br>Not often<br>Never<br>Missing	1<br>2<br>3<br>4<br>5<br>-9	No
B11. How often do you tell your child that soft drinks are bad for his/her teeth?	pb11	N1	Always<br>Often<br>Sometimes<br>Not often<br>Never<br>Missing	1<br>2<br>3<br>4<br>5<br>-9	No
B12. If I would like to dink soft drinks, I would restrain myself because of the presence of my child	pb12	N1	Always<br>Often<br>Sometimes<br>Not often<br>Never<br>Missing	1<br>2<br>3<br>4<br>5<br>-9	No
B13. If I prohibit my child from drinking soft drinks, (s)he tries to drink it anyway	pb13	N1	Always<br>Often<br>Sometimes<br>Not often<br>Never<br>Missing	1<br>2<br>3<br>4<br>5<br>-9	No
B14 If I prohibit my child from drinking soft drinks, I find it difficult ot stick to my rule(s), if (s)he starts negotiating	pb14	N1	Always<br>Often<br>Sometimes<br>Not often<br>Never<br>Missing	1<br>2<br>3<br>4<br>5<br>-9	No
B15 I give soft drinks to my child as a reard or to comfort him/her.	pb15	N1	Always<br>Often<br>Sometimes<br>Not often<br>Never<br>Missing	1<br>2<br>3<br>4<br>5<br>-9	No
B16. How often do you or your spouse drink soft drinks together with your child?	pb16	N1	Never<br>Less than once a week<br>Once a week<br>2-4 days a week<br>5-6 days a week<br>Every day<br>Every day, more than once a day<br>Missing	0<br>0,5<br>1<br>3<br>5,5<br>7<br>8<br>-9	No
B17. If the price of soft drinks were double, my child would drink less soft drinks	pb17	N1	I fully agree<br>I agree a bit<br>Neither agree nor disagree<br>I disagree a bit<br>I fully disagree<br>Missing	1<br>2<br>3<br>4<br>5<br>-9	No
B18. On average how much money do you give to your child to buy food and drinks per week? Please do not include money you save or spend on clothes for your child	pb18	N1	I don’t give money to my child<br>Less than 5€<br>Between 5€ and 10€<br>Between 11€ and 20€<br>Between 21€ and 30€<br>Between 31€ and 40€<br>Between 41€ and 50€<br>More than 51€<br>Missing	0<br>1<br>2<br>3<br>4<br>5<br>6<br>7<br>-9	No

B19. I would consider my child as being price conscious regarding food, snacks	pb19	N1	I fully agree<br>I agree a bit<br>Neither agree nor disagree<br>I disagree a bit<br>I fully disagree<br>Missing	1<br>2<br>3<br>4<br>5<br>-9	No
B20. I don't give my child some foods because they cost too much	pb20	N1	I fully agree<br>I agree a bit<br>Neither agree nor disagree<br>I disagree a bit<br>I fully disagree<br>Missing	1<br>2<br>3<br>4<br>5<br>-9	No
B21. What do you consider to be the three most important characteristics of your child's meal during school hours					
nutritious	pb21_nut	N1	Selected<br>Not selected<br>Missing	1<br>0<br>-9	No
provides energy	pb21_ene	N1	Selected<br>Not selected<br>Missing	1<br>0<br>-9	No
exhibits high variety	pb21_var	N1	Selected<br>Not selected<br>Missing	1<br>0<br>-9	No
satisfies my child's liking	pb21_lik	N1	Selected<br>Not selected<br>Missing	1<br>0<br>-9	No
reasonable price	pb21_pri	N1	Selected<br>Not selected<br>Missing	1<br>0<br>-9	No
home-prepared	pb21_hom	N1	Selected<br>Not selected<br>Missing	1<br>0<br>-9	No
organic	pb21_org	N1	Selected<br>Not selected<br>Missing	1<br>0<br>-9	No
vegetarian	pb21_veg	N1	Selected<br>Not selected<br>Missing	1<br>0<br>-9	No
taking into account religious requirements	pb21_rel	N1	Selected<br>Not selected<br>Missing	1<br>0<br>-9	No
C1. How many times a week do you usually drink fruit juices?	pc1	N1	Never<br>Less than once a week<br>Once a week<br>2-4 days a week<br>5-6 days a week<br>Every day<br>Every day, more than once a day<br>Missing	0<br>0,5<br>1<br>3<br>5,5<br>7<br>8<br>-9	No
C2. On a day that you drink fruit juice, how many glasses, or cartons do you drink on such a day?					No
a.Glasses or small bottles (250ml)	pc2_a	N1	None<br>1 glass/small bottle<br>2 glasses/small bottles<br>3 glasses/small bottles<br>4 glasses/small bottles	0<br>1<br>2<br>3<br>4	No

			5 or more glasses/small bottles<br>Not available (if pc1=0)<br>Missing	5<br>-8<br>-9	
b. Cartons (330ml)	pc2_b	N1	None<br>1 carton<br>2 cartons<br>3 cartons<br>4 cartons<br>5 or more cartons<br>Not available (if pc1=0)<br>Missing	0<br>1<br>2<br>3<br>4<br>5<br>-8<br>-9	No
C3. Drinking fruit juices is something that I do without even really thinking about it.	pc3	N1	I fully agree<br>I agree a bit<br>Neither agree nor disagree<br>I disagree a bit<br>I fully disagree<br>Not available (if pc1=0)<br>Missing	1<br>2<br>3<br>4<br>5<br>-8<br>-9	No
C4. There are fruit juices available at home for my child	pc4	N1	Always<br>Often<br>Sometimes<br>Not often<br>Never<br>Missing	1<br>2<br>3<br>4<br>5<br>-9	No
C5. I pay attention to the amount of fruit juices that my child drinks	pc5	N1	Always<br>Often<br>Sometimes<br>Not often<br>Never<br>Missing	1<br>2<br>3<br>4<br>5<br>-9	No
C6. If my child asks for fruit juices, I will give it to him/her	pc6	N1	Always<br>Often<br>Sometimes<br>Not often<br>Never<br>Missing	1<br>2<br>3<br>4<br>5<br>-9	No
C7. My child is allowed to take fruit juices, whenever (s)he wants	pc7	N1	Always<br>Often<br>Sometimes<br>Not often<br>Never<br>Missing	1<br>2<br>3<br>4<br>5<br>-9	No
C8. I negotiate with my child how much fruit juices (s)he is allowed to drink	pc8	N1	Always<br>Often<br>Sometimes<br>Not often<br>Never<br>Missing	1<br>2<br>3<br>4<br>5<br>-9	No
C9. How often do you tell your child that fruit juices are not good for him/her	pc9	N1	Always<br>Often<br>Sometimes<br>Not often<br>Never<br>Missing	1<br>2<br>3<br>4<br>5<br>-9	No
C10.How often do you tell your child that fruit juices can make him/her fat?	pc10	N1	Always<br>Often<br>Sometimes<br>Not often<br>Never<br>Missing	1<br>2<br>3<br>4<br>5<br>-9	No

C11. How often do you tell your child that soft drinks are bad for his/her teeth?	pc11	N1	Always<br>Often<br>Sometimes<br>Not often<br>Never<br>Missing	1<br>2<br>3<br>4<br>5<br>-9	No
C12. If I would like to drink fruit juices, I would restrain myself because of the presence of my child	pc12	N1	Always<br>Often<br>Sometimes<br>Not often<br>Never<br>Missing	1<br>2<br>3<br>4<br>5<br>-9	No
C13. If I prohibit my child from drinking fruit juices, (s)he tries to drink it anyway	pc13	N1	Always<br>Often<br>Sometimes<br>Not often<br>Never<br>Missing	1<br>2<br>3<br>4<br>5<br>-9	No
C14 If I prohibit my child from drinking fruit juices, I find it difficult ot stick to my rule(s), if (s)he starts negotiating	pc14	N1	Always<br>Often<br>Sometimes<br>Not often<br>Never<br>Missing	1<br>2<br>3<br>4<br>5<br>-9	No
C15 I give fruit juices to my child as a reward or to comfort him/her.	pc15	N1	Always<br>Often<br>Sometimes<br>Not often<br>Never<br>Missing	1<br>2<br>3<br>4<br>5<br>-9	No
C16. How often do you or your spouse drink fruit juices together with your child?	pc16	N1	Never<br>Less than once a week<br>Once a week<br>2-4 days a week<br>5-6 days a week<br>Every day<br>Every day, more than once a day<br>Missing	0<br>0,5<br>1<br>3<br>5,5<br>7<br>8<br>-9	No
D1. From Monday to Friday how many times do you usually eat breakfast?	pd1	N1	I never eat breakfast on a weekday<br>1 day<br>2 days<br>3 days<br>4 days<br>5 days<br>Missing	0<br>1<br>2<br>3<br>4<br>5<br>-9	No
D2. On how many days do you usually eat breakfast on the weekend?	pd2	N1	I never eat breakfast on weekendday<br>1 weekendday (Saterday OR Sunday)<br>2 weekenddays (Saterday AND Sunday)<br>Missing	0<br>1<br>2<br>-9	No
D3. Eating breakfast is something that I do without even really thinking about.	pd3	N1	I fully agree<br>I agree a bit<br>Neither agree nor disagree<br>I disagree a bit<br>I fully disagree<br>Missing	1<br>2<br>3<br>4<br>5<br>-9	No
D4. There are breakfast products (milk, cereals, bread etc) available at homefor my child	pd4	N1	Always<br>Often<br>Sometimes<br>Not often<br>Never	1<br>2<br>3<br>4<br>5	No

			Missing	-9	
D5. I encourage my child to have breakfast	pd5	N1	Always<br>Often<br>Sometimes<br>Not often<br>Never<br>Missing	1<br>2<br>3<br>4<br>5<br>-9	No
D6. I pay attention what kind of products my child is eating for breakfast	pd6	N1	Always<br>Often<br>Sometimes<br>Not often<br>Never<br>Missing	1<br>2<br>3<br>4<br>5<br>-9	No
D7. My chid is allowed to skip breakfast	pd7	N1	Always<br>Often<br>Sometimes<br>Not often<br>Never<br>Missing	1<br>2<br>3<br>4<br>5<br>-9	No
D8. I negotiate with my child on how much …	pd8	N1	Always<br>Often<br>Sometimes<br>Not often<br>Never<br>Missing	1<br>2<br>3<br>4<br>5<br>-9	No
D9. How often do you tell your child that eating breakfast is good for you	pd9	N1	Always<br>Often<br>Sometimes<br>Not often<br>Never<br>Missing	1<br>2<br>3<br>4<br>5<br>-9	No
D10. If I prohibit my child from skipping breakfast, (s)he tries to skip it anyway	pd10	N1	Always<br>Often<br>Sometimes<br>Not often<br>Never<br>Missing	1<br>2<br>3<br>4<br>5<br>-9	No
D11. If I prohibit my child from skipping breakfast, I find it difficult to stick to my rule(s) if (s)he starts negotiating	pd11	N1	Always<br>Often<br>Sometimes<br>Not often<br>Never<br>Missing	1<br>2<br>3<br>4<br>5<br>-9	No
D12. I praise my child if (s)he eats breakfast	pd12	N1	Always<br>Often<br>Sometimes<br>Not often<br>Never<br>Missing	1<br>2<br>3<br>4<br>5<br>-9	No
D13. How often do you eat breakfast with your parents/ care givers?	pd13	N1	Never<br>Once a week<br>2-4 days a week<br>5-6 days a week<br>Every day<br>Missing	0<br>1<br>3<br>5,5<br>7<br>-9	No
D14. How often do you eat lunch with your parents/ care givers?	pd14	N1	Never<br>Once a week<br>2-4 days a week<br>5-6 days a week<br>Every day	0<br>1<br>3<br>5,5<br>7	No

			Missing	-9	
D15. How often do you eat dinner with your parents/ care givers?	pd15	N1	Never<br>Once a week<br>2-4 days a week<br>5-6 days a week<br>Every day<br>Missing	0<br>1<br>3<br>5,5<br>7<br>-9	No
E1. I deliberately have smaller helpings as a means of controlling my weight	pe1	N1	I fully agree<br>I agree a bit<br>Neither agree nor disagree<br>I disagree a bit<br>I fully disagree<br>Missing	1<br>2<br>3<br>4<br>5<br>-9	No
E2. I do not eat certain foods because they make me fat	pe2	N1	I fully agree<br>I agree a bit<br>Neither agree nor disagree<br>I disagree a bit<br>I fully disagree<br>Missing	1<br>2<br>3<br>4<br>5<br>-9	No
E3. On a scale of 1 to 8 where 1 means no restrain in eating and 8 means total restraint, what rating would you give yourself?	pe3	N1	1 or lower<br>2<br>3<br>4<br>5<br>6<br>7<br>8 or higher<br>Missing	1<br>2<br>3<br>4<br>5<br>6<br>7<br>8<br>-9	No
F1. Do you have a job?	pf1	N1	I do have a job<br>I do not have a job<br>I work from home<br>Missing	1<br>2<br>3<br>-9	No
F2. How many days do you travel by car to work?	pf2	N1	I never travel by car to work<br>1 day per week<br>2 days per week<br>3 days per week<br>4 days per week<br>5 days per week<br>Not available (if pf1=2 or 3)<br>Missing	0<br>1<br>2<br>3<br>4<br>5<br>-8<br>-9	No
F3. How many days do you usually use public transport to go to work?	pf3	N1	I never travel by public transport to work<br>1 day per week<br>2 days per week<br>3 days per week<br>4 days per week<br>5 days per week<br>Not available (if pf1=2 or 3)<br>Missing	0<br>1<br>2<br>3<br>4<br>5<br>-8<br>-9	No
F4. How many days do you usually cycle to work?	pf4	N1	I never bike to work<br>1 day per week<br>2 days per week<br>3 days per week<br>4 days per week<br>5 days per week<br>Not available (if pf1=2 or 3)<br>Missing	0<br>1<br>2<br>3<br>4<br>5<br>-8<br>-9	No
F5. If you CYCLE to work, how long does it take you to cycle to work or to the public transport station?	pf5	N1	I never bike to work<br>1-5 minutes<br>6-10 minutes<br>11-15 minutes	0<br>1<br>2<br>3	No

			More than 15 minutes<br>Not available (if pf1=2 or 3)<br>Missing	4<br>-8<br>-9	
F6. How many days do you usually walk to work?	pf6	N1	I never walk to work<br>1 day per week<br>2 days per week<br>3 days per week<br>4 days per week<br>5 days per week<br>Not available (if pf1=2 or 3)<br>Missing	0<br>1<br>2<br>3<br>4<br>5<br>-8<br>-9	No
F7. If you WALK to work, how long does it take you to walk to work or to the public transport station?	pf7	N1	I never walk to work<br>1-5 minutes<br>6-10 minutes<br>11-15 minutes<br>More than 15 minutes<br>Not available (if pf1=2 or 3)<br>Missing	0<br>1<br>2<br>3<br>4<br>-8<br>-9	No
F8. I do not participate in any sports acivities	pf8_1	N1	Selected<br>Not selected<br>Missing	1<br>0<br>-9	No
F8. About how many days a week do you usually participate in physical activity /sports in your leisure time?					
Weekdays	pf8_wkday	N1	None at all<br>1 day per week<br>2 days per week<br>3 days per week<br>4 days per week<br>5 days per week<br>Not available (if pf8_1 = 1)<br>Missing	0<br>1<br>2<br>3<br>4<br>5<br>-8<br>-9	No
Weekends	pf8_wkend	N1	None at all<br>1 day (Saturday OR Sunday)<br>2 days (Saturday AND Sunday)<br>Not available (if pf8_1 = 1)<br>Missing	0<br>1<br>2<br>-8<br>-9	No
F9. About how much time a week do you participate in physical activities/sports in your leisure time?					
Weekdays	pf9_wkday	N1	None at all<br>30 minutes/week<br>1 hour/week<br>2 hours/week<br>3 hours/week<br>4 hours/week<br>5 hours/week<br>Not available (if pf8_1 = 1)<br>Missing	0<br>0,5<br>1<br>2<br>3<br>4<br>5<br>-8<br>-9	No
Weekends	pf9_wkend	N1	None at all<br>30 minutes/week<br>1 hour/week<br>2 hours/week<br>3 hours/week<br>4 hours/week<br>5 hours/week<br>Not available (if pf8_1 = 1)<br>Missing	0<br>0,5<br>1<br>2<br>3<br>4<br>5<br>-8<br>-9	No

F10. Physical activities/sport sctivities is something that I do without really thinking about	pf10	N1	I fully agree<br>I agree a bit<br>Neither agree nor disagree<br>I disagree a bit<br>I fully disagree<br>Not available (if pf8_1 = 1)<br>Missing	1<br>2<br>3<br>4<br>5<br>-9	No
F11. I pay for my child to take part in physical activity /sports	pf11	N1	Always<br>Often<br>Sometimes<br>Not often<br>Never<br>Missing	1<br>2<br>3<br>4<br>5<br>-9	No
F12. I bring my child to physical activity/sports sessions	pf12	N1	Always<br>Often<br>Sometimes<br>Not often<br>Never<br>Missing	1<br>2<br>3<br>4<br>5<br>-9	No
F13. I encourage my child to take part in physical activity /sports	pf13	N1	Always<br>Often<br>Sometimes<br>Not often<br>Never<br>Missing	1<br>2<br>3<br>4<br>5<br>-9	No
F14. I pay attention that my child does enough physical activity/sports	pf14	N1	Always<br>Often<br>Sometimes<br>Not often<br>Never<br>Missing	1<br>2<br>3<br>4<br>5<br>-9	No
F15. My child is allowed to skip physical activity /sport sessions whenever (s)he wants	pf15	N1	Always<br>Often<br>Sometimes<br>Not often<br>Never<br>Missing	1<br>2<br>3<br>4<br>5<br>-9	No
F16. I negotiate woth my child how much physical activity…	pf16	N1	Always<br>Often<br>Sometimes<br>Not often<br>Never<br>Missing	1<br>2<br>3<br>4<br>5<br>-9	No
F17. How often do you tell your child that physical activity/ sports are good for him/her	pf17	N1	Always<br>Often<br>Sometimes<br>Not often<br>Never<br>Missing	1<br>2<br>3<br>4<br>5<br>-9	No
F18. If I try to prohibit my child from not taking part in a physical activity/sport session, (s)he will try to skip it anyway	pf18	N1	Always<br>Often<br>Sometimes<br>Not often<br>Never<br>Missing	1<br>2<br>3<br>4<br>5<br>-9	No
F19. If I rry to prohibit my child from skipping a physical activity/sport session, I find it difficult to stick to my rule(s) if (s)he starts negotiating	pf19	N1	Always<br>Often<br>Sometimes<br>Not often<br>Never	1<br>2<br>3<br>4<br>5	No

			Missing	-9	
F20. I praise my child if (s)he takes part in physical activity /sports	pf20	N1	Always<br>Often<br>Sometimes<br>Not often<br>Never<br>Missing	1<br>2<br>3<br>4<br>5<br>-9	No
F21. I punish my child by not allowing him/her to take part in his/her physical activity sessions/sports	pf21	N1	Always<br>Often<br>Sometimes<br>Not often<br>Never<br>Missing	1<br>2<br>3<br>4<br>5<br>-9	No
F22. I set a time limit on how much time of physical activity/sports my child can do in order to devote more time to his/her homework or other important things	pf22	N1	I fully agree<br>I agree a bit<br>Neither agree nor disagree<br>I disagree a bit<br>I fully disagree<br>Missing	1<br>2<br>3<br>4<br>5<br>-9	No
F23. I do not allow my child to take part in physical activity/sports in his/her free time so that (s)he can concentrate on his/her studies.	pf23	N1	I fully agree<br>I agree a bit<br>Neither agree nor disagree<br>I disagree a bit<br>I fully disagree<br>Missing	1<br>2<br>3<br>4<br>5<br>-9	No
F24. How often do you and/or your spouse/partner participate in physical activity/sports togheter with your child.	pf24	N1	Never<br>Less than once a week<br>Once a week<br>2-4 days a week<br>5-6 days a week<br>Every day<br>Missing	0<br>0,5<br>1<br>3<br>5,5<br>7<br>-9	No
F25. I let my child participate in activity/sports less than I would like, because it is too expensive	pf25	N1	I fully agree<br>I agree a bit<br>Neither agree nor disagree<br>I disagree a bit<br>I fully disagree<br>Missing	1<br>2<br>3<br>4<br>5<br>-9	No
G1.About how many hours a day do you usually watch television					
Weekdays	pg1_a	N1	None at all<br>30 minutes/week<br>1 hour/week<br>1,5 hour/week<br>2 hours/week<br>2,5 hours/week<br>3 hours/week<br>3,5 hours/week<br>4 hours/week<br>Missing	0<br>0,5<br>1<br>1,5<br>2<br>2,5<br>3<br>3,5<br>4<br>-9	No
Weekenddays	pg1_b	N1	None at all<br>30 minutes/week<br>1 hour/week<br>1,5 hour/week<br>2 hours/week<br>2,5 hours/week<br>3 hours/week<br>3,5 hours/week<br>4 hours/week	0<br>0,5<br>1<br>1,5<br>2<br>2,5<br>3<br>3,5<br>4	No

			Missing	-9	
G2.About houw many hours a day do you usually use your computer for activities like chatting online,internet,emailing or playing games on a computer,games consule					
Weekdays	pg2_a	N1	None at all<br>30 minutes/week<br>1 hour/week<br>1,5 hour/week<br>2 hours/week<br>2,5 hours/week<br>3 hours/week<br>3,5 hours/week<br>4 hours/week<br>Missing	0<br>0,5<br>1<br>1,5<br>2<br>2,5<br>3<br>3,5<br>4<br>-9	No
Weekenddays	pg2_b	N1	None at all<br>30 minutes/week<br>1 hour/week<br>1,5 hour/week<br>2 hours/week<br>2,5 hours/week<br>3 hours/week<br>3,5 hours/week<br>4 hours/week<br>Missing	0<br>0,5<br>1<br>1,5<br>2<br>2,5<br>3<br>3,5<br>4<br>-9	No
G3.About houw many hours a day do you usually use your mobile phone for calling,texting,playing games or surfing on the internet during leisure time					
Weekdays	pg3_a	N1	None at all<br>30 minutes/week<br>1 hour/week<br>1,5 hour/week<br>2 hours/week<br>2,5 hours/week<br>3 hours/week<br>3,5 hours/week<br>4 hours/week<br>Missing	0<br>0,5<br>1<br>1,5<br>2<br>2,5<br>3<br>3,5<br>4<br>-9	No
Weekenddays	pg3_b	N1	None at all<br>30 minutes/week<br>1 hour/week<br>1,5 hour/week<br>2 hours/week<br>2,5 hours/week<br>3 hours/week<br>3,5 hours/week<br>4 hours/week<br>Missing	0<br>0,5<br>1<br>1,5<br>2<br>2,5<br>3<br>3,5<br>4<br>-9	No
G4.Watching television is something i do without even really thinking about.	pg4	N1	I fully agree<br>I agree a bit<br>Neither agree nor disagree<br>I disagree a bit<br>I fully disagree<br>Missing	1<br>2<br>3<br>4<br>5<br>-9	No
G5.In general, how often do you					

watch television during the following meals?					
Breakfast	pg5_a	N1	Always<br>Often<br>Sometimes<br>Not often<br>Never<br>Missing	1<br>2<br>3<br>4<br>5<br>-9	No
Lunch	pg5_b	N1	Always<br>Often<br>Sometimes<br>Not often<br>Never<br>Missing	1<br>2<br>3<br>4<br>5<br>-9	No
Dinner	pg5_c	N1	Always<br>Often<br>Sometimes<br>Not often<br>Never<br>Missing	1<br>2<br>3<br>4<br>5<br>-9	No
G6. TV/video/dvd is available in my childs's room	pg6	N1	Yes<br>No<br>Missing	1<br>0<br>-9	No
G7.I pay attention to the amount of time my child watches TV/video/dvd.	pg7	N1	Always<br>Often<br>Sometimes<br>Not often<br>Never<br>Missing	1<br>2<br>3<br>4<br>5<br>-9	No
G8.If my child asks if (s)he is allowed to watch TV/video/dvd,I will allow it.	pg8	N1	Always<br>Often<br>Sometimes<br>Not often<br>Never<br>Missing	1<br>2<br>3<br>4<br>5<br>-9	No
G9.My child is allowed to watch TV/video/dvd whenever (s)he wants.	pg9	N1	Always<br>Often<br>Sometimes<br>Not often<br>Never<br>Missing	1<br>2<br>3<br>4<br>5<br>-9	No
G10. I negotiate with my child how much TV/video/dvd (s)he is allowed to watch	pg10	N1	Always<br>Often<br>Sometimes<br>Not often<br>Never<br>Missing	1<br>2<br>3<br>4<br>5<br>-9	No
G11.How often do you tell your child that watching TV/video/dvd is not good for him/her?	pg11	N1	Always<br>Often<br>Sometimes<br>Not often<br>Never<br>Missing	1<br>2<br>3<br>4<br>5<br>-9	No
G12.How often do you tell your child that watching TV/video/dvd can make him/her fat?	pg12	N1	Always<br>Often<br>Sometimes<br>Not often<br>Never<br>Missing	1<br>2<br>3<br>4<br>5<br>-9	No
G13.How often do you tell your	pg13	N1	Always	1	No

child that watching TV/video/dvd is bad for him/her sight?			Often<br>Sometimes<br>Not often<br>Never<br>Missing	2<br>3<br>4<br>5<br>-9	
G14.If i would like to watch TV/video/dvd i would restrain myself because of het presence of my child?	pg14	N1	Always<br>Often<br>Sometimes<br>Not often<br>Never<br>Missing	1<br>2<br>3<br>4<br>5<br>-9	No
G15.If i prohibit my child from watching TV/video/dvd,(s)he tries to watch anyway.	pg15	N1	Always<br>Often<br>Sometimes<br>Not often<br>Never<br>Missing	1<br>2<br>3<br>4<br>5<br>-9	No
G16.If i prohibit my child from watching TV/video/dvd I find it difficult to stick to my rule(s) if (s)he starts negotiating.	pg16	N1	Always<br>Often<br>Sometimes<br>Not often<br>Never<br>Missing	1<br>2<br>3<br>4<br>5<br>-9	No
G17.I allow my child to watch TV/Video/dvd as a reward or to comfort him/her.	pg17	N1	Always<br>Often<br>Sometimes<br>Not often<br>Never<br>Missing	1<br>2<br>3<br>4<br>5<br>-9	No
G18.How often do you (one parent/spouse/partner or both)watch television together with your child?	pg18	N1	Never<br>Less than once a week<br>Once a week<br>2-4 days a week<br>5-6 days a week<br>Every day<br>Every day, more than once a day<br>Missing	0<br>0,5<br>1<br>3<br>5,5<br>7<br>8<br>-9	No
H1.What do you think about your child's weight?	ph1	N1	My child’s weight is OK<br>My child’s weight is a bit too much<br>My child’s weight is way too much<br>My child’s weight is a bit too little<br>My child’s weight is way too little<br>Missing	1<br>2<br>3<br>4<br>5<br>-9	No
H2.Does your child have a set daily routine for bedtime?	ph2	N1	Yes<br>No<br>Missing	1<br>0<br>-9	No
H3.How many hours of sleep does your chold usaully have during the night?					
Weekdays	ph3_a	N1	6 hours or less per night<br>7 hours per night<br>8 hours per night<br>9 hours per night<br>10 hours per night<br>More than 10 hours or less per night<br>Missing	1<br>2<br>3<br>4<br>5<br>6<br>-9	No
Weekenddays	ph3_b	N1	6 hours or less per night<br>7 hours per night<br>8 hours per night<br>9 hours per night	1<br>2<br>3<br>4	No

			10 hours per night<br>More than 10 hours or less per night<br>Missing	5<br>6<br>-9	

Codeboek ENERGY Audit Instrument

Question	Variable name	Type	Value	Code	Compulsory

**General information: Forms completed**					
School ID	SCHOOL_ID	N3	001-998	001-998	Yes
Date of data collection					
day	DATE_D	date	1-31<br>missing	1-31<br>-9	No
month	DATE_M	date	January<br>February<br>March<br>April<br>May<br>June<br>July<br>August<br>September<br>October<br>November<br>December<br>Missing	1<br>2<br>3<br>4<br>5<br>6<br>7<br>8<br>9<br>10<br>11<br>12<br>-9	No
year	DATE_Y	date	2010<br>Missing	2010<br>-9	No
Observer	OBS	A50	Missing	-9	No
Start time	S_TIME	date	00:00-24:00<br>Missing	00.00-24.00<br>-9	No
End time	E_TIME	date	00:00-24:00<br>Missing	00.00-24.00<br>-9	No
Form 1A: Canteen/shop/ kiosk/restaurant interview	F1A	N1	Yes, fully completed<br>Yes, partly completed<br>n/a<br>Missing	1<br>2<br>3<br>-9	No
Comments	F1A_C	A100	Missing	-9	No
Form 1B: Food/drink registration	F1B	N1	Yes, fully completed<br>Yes, partly completed<br>n/a<br>Missing	1<br>2<br>3<br>-9	No
Comments	F1B_C	A100	Missing	-9	No
Form 2A: Vending machines - interview	F2A	N1	Yes, fully completed<br>Yes, partly completed<br>n/a<br>Missing	1<br>2<br>3<br>-9	No
Comments	F2A_C	A100	Missing	-9	No
Form 2B: Food/drink registration	F2B	N1	Yes, fully completed<br>Yes, partly completed<br>n/a<br>Missing	1<br>2<br>3<br>-9	No
Comments	F2B_C	A100	Missing	-9	No
Form 3: Subscription programmes	F3	N1	Yes, fully completed<br>Yes, partly completed<br>n/a<br>Missing	1<br>2<br>3<br>-9	No
Comments	F3_C	A100	Missing	-9	No
Form 4: Commercial advertising	F4	N1	Yes, fully completed<br>Yes, partly completed<br>n/a<br>Missing	1<br>2<br>3<br>-9	No
Comments	F4_C	A100	Missing	-9	No
Form 5: Bicycle parking	F5	N1	Yes, fully completed<br>Yes, partly completed<br>n/a	1<br>2<br>3	No

			Missing	-9	
Comments	F5_C	A100	Missing	-9	No
Form 6: Equipment for recess	F6	N1	Yes, fully completed<br>Yes, partly completed<br>n/a<br>Missing	1<br>2<br>3<br>-9	
Comments	F6_C	A100	Missing	-9	No
Form 7: Indoor PA facilities	F7	N1	Yes, fully completed<br>Yes, partly completed<br>n/a<br>Missing	1<br>2<br>3<br>-9	No
Comments	F7_C	A100	Missing	-9	No
Form 8: Outdoor PA facilities	F8	N1	Yes, fully completed<br>Yes, partly completed<br>n/a<br>Missing	1<br>2<br>3<br>-9	No
Comments	F8_C	A100	Missing	-9	No
Form 9: Other info outdoor area	F9	N1	Yes, fully completed<br>Yes, partly completed<br>n/a<br>Missing	1<br>2<br>3<br>-9	No
Comments	F9_C	A100	Missing	-9	No
**Form 1A: Canteen/shop/ kiosk/restaurant interview**					
School ID	F1A_SCH	N3	001-998	001-998	Yes
Observer	F1A_OBS	A50	Missing	-9	No
Begin time	F1A_ST	date	00:00-24:00<br>Missing	00.00-24.00<br>-9	No
End time	F1A_ET	date	00:00-24:00<br>Missing	00.00-24.00<br>-9	No
1. The person who will be interviewed is the	F1A_1	N1	Canteen/school shop/ kiosk/administrator<br>Principal/vice principal<br>Other school staff<br>Missing	1<br>2<br>3<br>-9	No
If other, who	F1A_1_C	A100	Missing	-9	No
2. Who owns the canteen/school shop/ kiosk/restaurant?					
The school	F1A_2a	N1	Selected<br>Not selected<br>Missing	1<br>0<br>-9	No
Parents	F1A_2b	N1	Selected<br>Not selected<br>Missing	1<br>0<br>-9	No
Private	F1A_2c	N1	Selected<br>Not selected<br>Missing	1<br>0<br>-9	No
Other	F1A_2d	N1	Selected<br>Not selected<br>Missing	1<br>0<br>-9	No
If other, who	F1A_2_C	A100	Missing	-9	No
3. Are you the main person who is running the canteen/school shop/kiosk/ restaurant?	F1A_3	N1	Yes<br>No<br>Partly<br>Missing	1<br>0<br>2<br>-9	No
If no and/or partly, indicate who is the main person	F1A_3_C	A100	Missing	-9	No
4. Who decides what is offered in the	F1A_4	N1	The canteen/school shop/kiosk	1	No

canteen/school shop/kiosk/ restaurant?			administrator<br>The principal/vice principal<br>The government<br>Parents<br>Pupils<br>Other<br>Missing	 2<br>3<br>4<br>5<br>6<br>-9	
If other, who	F1A_4_C	A100	Missing	-9	No
5. How many days per week is the canteen/school shop/kiosk/ restaurant operated?	F1A_5	N1	1-7<br>Missing	1-7<br>-9	No
6. What are its hours of operation?					
Open time	F1A_6a	date	00:00-24:00<br>Missing	00.00-24.00<br>-9	No
Close time	F1A_6b	date	00:00-24:00<br>Missing	00.00-24.00<br>-9	No
7. Does the canteen/restaurant sell/offer any complete meals to all/a large proportion of the pupils?					
Yes, for breakfast	F1A_7a	N1	Selected<br>Not selected<br>Missing	1<br>0<br>-9	No
Yes, for lunch	F1A_7b	N1	Selected<br>Not selected<br>Missing	1<br>0<br>-9	No
Yes, other	F1A_7c	N1	Selected<br>Not selected<br>Missing	1<br>0<br>-9	No
No	F1A_7d	N1	Selected<br>Not selected<br>Missing	1<br>0<br>-9	No
If other, indicate	F1A_7_C	A100	Missing	-9	No
8. How are the meals prepared?	F1A_8	N1	Cooked in the canteen/school shop/kiosk/ restaurant<br>Professional catering service<br>Other<br>Not available (if F1A_7d = 1)<br>Missing	1<br><br>2<br>3<br>Empty<br>-9	No
If other, how	F1A_8_C	A100	Missing	-9	No
9. Who decides what is offered at these complete meals (food and drinks)?					
School management	F1A_9a	N1	Selected<br>Not selected<br>Not available (if F1A_7d = 1)	1<br>0<br>-9	No
The canteen/restaurant manager	F1A_9b	N1	Selected<br>Not selected<br>Not available (if F1A_7d = 1)	1<br>0<br>-9	No
The government	F1A_9c	N1	Selected<br>Not selected<br>Not available (if F1A_7d = 1)	1<br>0<br>-9	No
Parents	F1A_9d	N1	Selected<br>Not selected<br>Not available (if F1A_7d = 1)	1<br>0<br>-9	No
Pupils	F1A_9e	N1	Selected<br>Not selected<br>Not available (if F1A_7d = 1)	1<br>0<br>-9	No
Other	F1A_9f	N1	Selected	1	No

			Not selected<br>Not available (if F1A_7d = 1)	0<br>-9	
If other, who	F1A_9_C	A100	Missing	-9	No
10. Is there a weekly/monthly menu available for what foods are offered?	F1A_10	N1	Yes<br>No<br>Not available (if F1A_7d = 1)	1<br>0<br>-9	No
Copy of the weekly menu	F1A_10_C	A50	(number of image)<br>Missing	 -9	No
11. What is usually offered to drink with the meal?					
Nothing	F1A_11a	N1	Selected<br>Not selected<br>Not available (if F1A_7d = 1)	1<br>0<br>-9	No
Water	F1A_11b	N1	Selected<br>Not selected<br>Not available (if F1A_7d = 1)	1<br>0<br>-9	No
Milk	F1A_11c	N1	Selected<br>Not selected<br>Not available (if F1A_7d = 1)	1<br>0<br>-9	No
Other	F1A_11d	N1	Selected<br>Not selected<br>Not available (if F1A_7d = 1)	1<br>0<br>-9	No
If other, what	F1A_11_C	A100	Missing	-9	No
12. What are the main product groups sold (free choice) in the canteen/school shop/kiosk/ restaurant?					
Sandwiches	F1A_12a	N1	Selected<br>Not selected<br>Missing	1<br>0<br>-9	No
Hot foods	F1A_12b	N1	Selected<br>Not selected<br>Missing	1<br>0<br>-9	No
Drinks	F1A_12c	N1	Selected<br>Not selected<br>Missing	1<br>0<br>-9	No
Salt/sweet snacks/cakes	F1A_12d	N1	Selected<br>Not selected<br>Missing	1<br>0<br>-9	No
Other	F1A_12e	N1	Selected<br>Not selected<br>Missing	1<br>0<br>-9	No
If other, explain	F1A_12_C	A100	Missing	-9	No
13. What are the three most sold food products in the canteen/school shop/kiosk/ restaurant?					
1.	F1A_13a	A50	Missing	-9	No
2.	F1A_13b	A50	Missing	-9	No
3.	F1A_13c	A50	Missing	-9	No
14. What are the three most sold drink items in the canteen/school shop/kiosk/ restaurant?					
1.	F1A_14a	A50	Missing	-9	No
2.	F1A_14b	A50	Missing	-9	No
3.	F1A_14c	A50	Missing	-9	No
15. Is there fresh drinking water offered in the canteen/school shop/kiosk/ restaurant?	F1A_15	N1	No<br>Yes, for free<br>Yes, for sale<br>Missing	0<br>1<br>2<br>-9	No

16. Is it possible for students to stay in canteen/school shop/kiosk/ restaurant before/after breaks?	F1A_16	N1	Yes<br>No<br>n/a<br>Missing	1<br>0<br>2<br>-9	No
If no or n/a, explain why	F1A_16_C	A100	Missing	-9	No
17. Who decides what is offered in the canteen/school shop/kiosk/ restaurant?	F1A_17	N1	The school management<br>The canteen/school shop/kiosk/ restaurant manager<br>The government<br>Parents<br>Pupils<br>Other<br>Missing	1<br>2<br>3<br>4<br>5<br>6<br>-9	No
If other, who	F1A_17_C	A100	Missing	-9	No
18. Does the school make profit on canteen/school shop/kiosk/ restaurant from products' sales or as rent to the running company/person who is running the canteen/school shop?	F1A_18	N1	Yes<br>No<br>n/a<br>Missing	1<br>0<br>2<br>-9	No
If yes, how much	F1A_18_C	A100	Missing	-9	No
19. How does the school use that profit?	F1A_19	A50	Not available	-9	No
**Form 1B: Canteen/school shop/kiosk/ restaurant - Food/drink registration**					
School ID	F1B_SCH	N3	001-998	001-998	Yes
Observer	F1B_OBS	A50	Missing	-9	No
Begin time	F1B_ST	date	00:00-24:00<br>Missing	00.00-24.00<br>-9	No
End time	F1B_ET	date	00:00-24:00<br>Missing	00.00-24.00<br>-9	No
**Bread products**					
Country specific product 1	F1B_1a	A50	Missing	-9	No
Gr./portion	F1B_1b	N3	1-900<br>Missing	1-900<br>-9	No
Kcal/portion	F1B_1c	N3	1-900<br>Missing	1-900<br>-9	No
Yes	F1B_1d	N1	Selected<br>Not selected<br>Missing	1<br>0<br>-9	No
Nr. of products	F1B_1e	N2	0-99<br>Missing	0-99<br>-9	No
Comments	F1B_1_C	A100	Missing	-9	No
Country specific product 2	F1B_2a	A50	Missing	-9	No
Gr./portion	F1B_2b	N3	1-900<br>Missing	1-900<br>-9	No
Kcal/portion	F1B_2c	N3	1-900<br>Missing	1-900<br>-9	No
Yes	F1B_2d	N1	Selected<br>Not selected<br>Missing	1<br>0<br>-9	No
Nr. of products	F1B_2e	N2	0-99<br>Missing	0-99<br>-9	No
Comments	F1B_2_C	A100	Missing	-9	No
Country specific product 3	F1B_3a	A50	Missing	-9	No
Gr./portion	F1B_3b	N3	1-900<br>Missing	1-900<br>-9	No
Kcal/portion	F1B_3c	N3	1-900	1-900	No

			Missing	-9	
Yes	F1B_3d	N1	Selected<br>Not selected<br>Missing	1<br>0<br>-9	No
Nr. of products	F1B_3e	N2	0-99<br>Missing	0-99<br>-9	No
Comments	F1B_3_C	A100	Missing	-9	No
Country specific product 4	F1B_4a	A50	Missing	-9	No
Gr./portion	F1B_4b	N3	1-900<br>Missing	1-900<br>-9	No
Kcal/portion	F1B_4c	N3	1-900<br>Missing	1-900<br>-9	No
Yes	F1B_4d	N1	Selected<br>Not selected<br>Missing	1<br>0<br>-9	No
Nr. of products	F1B_4e	N2	0-99<br>Missing	0-99<br>-9	No
Comments	F1B_4_C	A100	Missing	-9	No
Country specific product 5	F1B_5a	A50	Missing	-9	No
Gr./portion	F1B_5b	N3	1-900<br>Missing	1-900<br>-9	No
Kcal/portion	F1B_5c	N3	1-900<br>Missing	1-900<br>-9	No
Yes	F1B_5d	N1	Selected<br>Not selected<br>Missing	1<br>0<br>-9	No
Nr. of products	F1B_5e	N2	0-99<br>Missing	0-99<br>-9	No
Comments	F1B_5_C	A100	Missing	-9	No
Country specific product 6	F1B_6a	A50	Missing	-9	No
Gr./portion	F1B_6b	N3	1-900<br>Missing	1-900<br>-9	No
Kcal/portion	F1B_6c	N3	1-900<br>Missing	1-900<br>-9	No
Yes	F1B_6d	N1	Selected<br>Not selected<br>Missing	1<br>0<br>-9	No
Nr. of products	F1B_6e	N2	0-99<br>Missing	0-99<br>-9	No
Comments	F1B_6_C	A100	Missing	-9	No
Country specific product 7	F1B_7a	A50	Missing	-9	No
Gr./portion	F1B_7b	N3	1-900<br>Missing	1-900<br>-9	No
Kcal/portion	F1B_7c	N3	1-900<br>Missing	1-900<br>-9	No
Yes	F1B_7d	N1	Selected<br>Not selected<br>Missing	1<br>0<br>-9	No
Nr. of products	F1B_7e	N2	0-99<br>Missing	0-99<br>-9	No
Comments	F1B_7_C	A100	Missing	-9	No
Country specific product 8	F1B_8a	A50	Missing	-9	No
Gr./portion	F1B_8b	N3	1-900<br>Missing	1-900<br>-9	No
Kcal/portion	F1B_8c	N3	1-900<br>Missing	1-900<br>-9	No
Yes	F1B_8d	N1	Selected<br>Not selected<br>Missing	1<br>0<br>-9	No

Nr. of products	F1B_8e	N2	0-99<br>Missing	0-99<br>-9	No
Comments	F1B_8_C	A100	Missing	-9	No
Country specific product 9	F1B_9a	A50	Missing	-9	No
Gr./portion	F1B_9b	N3	1-900<br>Missing	1-900<br>-9	No
Kcal/portion	F1B_9c	N3	1-900<br>Missing	1-900<br>-9	No
Yes	F1B_9d	N1	Selected<br>Not selected<br>Missing	1<br>0<br>-9	No
Nr. of products	F1B_9e	N2	0-99<br>Missing	0-99<br>-9	No
Comments	F1B_9_C	A100	Missing	-9	No
Country specific product 10	F1B_10a	A50	Missing	-9	No
Gr./portion	F1B_10b	N3	1-900<br>Missing	1-900<br>-9	No
Kcal/portion	F1B_10c	N3	1-900<br>Missing	1-900<br>-9	No
Yes	F1B_10d	N1	Selected<br>Not selected<br>Missing	1<br>0<br>-9	No
Nr. of products	F1B_10e	N2	0-99<br>Missing	0-99<br>-9	No
Comments	F1B_10_C	A100	Missing	-9	No
Country specific product 11	F1B_11a	A50	Missing	-9	No
Gr./portion	F1B_11b	N3	1-900<br>Missing	1-900<br>-9	No
Kcal/portion	F1B_11c	N3	1-900<br>Missing	1-900<br>-9	No
Yes	F1B_11d	N1	Selected<br>Not selected<br>Missing	1<br>0<br>-9	No
Nr. of products	F1B_11e	N2	0-99<br>Missing	0-99<br>-9	No
Comments	F1B_11_C	A100	Missing	-9	No
**Drinks**					
Country specific product 12	F1B_12a	A50	Missing	-9	No
ml/bottle, can, package	F1B_12b	N3	1-900<br>Missing	1-900<br>-9	No
Kcal/portion	F1B_12c	N3	1-900<br>Missing	1-900<br>-9	No
Yes	F1B_12d	N1	Selected<br>Not selected<br>Missing	1<br>0<br>-9	No
Nr. of products	F1B_12e	N2	0-99<br>Missing	0-99<br>-9	No
Comments	F1B_12_C	A100	Missing	-9	No
Country specific product 13	F1B_13a	A50	Missing	-9	No
ml/bottle, can, package	F1B_13b	N3	1-900<br>Missing	1-900<br>-9	No
Kcal/portion	F1B_13c	N3	1-900<br>Missing	1-900<br>-9	No
Yes	F1B_13d	N1	Selected<br>Not selected<br>Missing	1<br>0<br>-9	No
Nr. of products	F1B_13e	N2	0-99<br>Missing	0-99<br>-9	No
Comments	F1B_13_C	A100	Missing	-9	No

Country specific product 14	F1B_14a	A50	Missing	-9	No
ml/bottle, can, package	F1B_14b	N3	1-900<br>Missing	1-900<br>-9	No
Kcal/portion	F1B_14c	N3	1-900<br>Missing	1-900<br>-9	No
Yes	F1B_14d	N1	Selected<br>Not selected<br>Missing	1<br>0<br>-9	No
Nr. of products	F1B_14e	N2	0-99<br>Missing	0-99<br>-9	No
Comments	F1B_14_C	A100	Missing	-9	No
Country specific product 15	F1B_15a	A50	Missing	-9	No
ml/bottle, can, package	F1B_15b	N3	1-900<br>Missing	1-900<br>-9	No
Kcal/portion	F1B_15c	N3	1-900<br>Missing	1-900<br>-9	No
Yes	F1B_15d	N1	Selected<br>Not selected<br>Missing	1<br>0<br>-9	No
Nr. of products	F1B_15e	N2	0-99<br>Missing	0-99<br>-9	No
Comments	F1B_15_C	A100	Missing	-9	No
Country specific product 16	F1B_16a	A50	Missing	-9	No
ml/bottle, can, package	F1B_16b	N3	1-900<br>Missing	1-900<br>-9	No
Kcal/portion	F1B_16c	N3	1-900<br>Missing	1-900<br>-9	No
Yes	F1B_16d	N1	Selected<br>Not selected<br>Missing	1<br>0<br>-9	No
Nr. of products	F1B_16e	N2	0-99<br>Missing	0-99<br>-9	No
Comments	F1B_16_C	A100	Missing	-9	No
Country specific product 17	F1B_17a	A50	Missing	-9	No
ml/bottle, can, package	F1B_17b	N3	1-900<br>Missing	1-900<br>-9	No
Kcal/portion	F1B_17c	N3	1-900<br>Missing	1-900<br>-9	No
Yes	F1B_17d	N1	Selected<br>Not selected<br>Missing	1<br>0<br>-9	No
Nr. of products	F1B_17e	N2	0-99<br>Missing	0-99<br>-9	No
Comments	F1B_17_C	A100	Missing	-9	No
Country specific product 18	F1B_18a	A50	Missing	-9	No
ml/bottle, can, package	F1B_18b	N3	1-900<br>Missing	1-900<br>-9	No
Kcal/portion	F1B_18c	N3	1-900<br>Missing	1-900<br>-9	No
Yes	F1B_18d	N1	Selected<br>Not selected<br>Missing	1<br>0<br>-9	No
Nr. of products	F1B_18e	N2	0-99<br>Missing	0-99<br>-9	No
Comments	F1B_18_C	A100	Missing	-9	No
Country specific product 19	F1B_19a	A50	Missing	-9	No
ml/bottle, can, package	F1B_15b	N3	1-900<br>Missing	1-900<br>-9	No
Kcal/portion	F1B_19c	N3	1-900	1-900	No

			Missing	-9	
Yes	F1B_19d	N1	Selected<br>Not selected<br>Missing	1<br>0<br>-9	No
Nr. of products	F1B_19e	N2	0-99<br>Missing	0-99<br>-9	No
Comments	F1B_19_C	A100	Missing	-9	No
Country specific product 20	F1B_20a	A50	Missing	-9	No
ml/bottle, can, package	F1B_20b	N3	1-900<br>Missing	1-900<br>-9	No
Kcal/portion	F1B_20c	N3	1-900<br>Missing	1-900<br>-9	No
Yes	F1B_20d	N1	Selected<br>Not selected<br>Missing	1<br>0<br>-9	No
Nr. of products	F1B_20e	N2	0-99<br>Missing	0-99<br>-9	No
Comments	F1B_20_C	A100	Missing	-9	No
Country specific product 21	F1B_21a	A50	Missing	-9	No
ml/bottle, can, package	F1B_21b	N3	1-900<br>Missing	1-900<br>-9	No
Kcal/portion	F1B_21c	N3	1-900<br>Missing	1-900<br>-9	No
Yes	F1B_21d	N1	Selected<br>Not selected<br>Missing	1<br>0<br>-9	No
Nr. of products	F1B_21e	N2	0-99<br>Missing	0-99<br>-9	No
Comments	F1B_21_C	A100	Missing	-9	No
Country specific product 22	F1B_22a	A50	Missing	-9	No
ml/bottle, can, package	F1B_22b	N3	1-900<br>Missing	1-900<br>-9	No
Kcal/portion	F1B_22c	N3	1-900<br>Missing	1-900<br>-9	No
Yes	F1B_22d	N1	Selected<br>Not selected<br>Missing	1<br>0<br>-9	No
Nr. of products	F1B_22e	N2	0-99<br>Missing	0-99<br>-9	No
Comments	F1B_22_C	A100	Missing	-9	No
**Snacks/cakes/candies**					
Country specific product 23	F1B_23a				
Gr./portion	F1B_23b	N3	1-900<br>Missing	1-900<br>-9	
Kcal/portion	F1B_23c	N3	1-900<br>Missing	1-900<br>-9	
Yes	F1B_23d	N1	Selected<br>Not selected<br>Missing	1<br>0<br>-9	No
Nr. of products	F1B_23e	N2	0-99<br>Missing	0-99<br>-9	No
Comments	F1B_23_C	A100	Missing	-9	No
Country specific product 24	F1B_24a	A50	Missing	-9	No
Gr./portion	F1B_24b	N3	1-900<br>Missing	1-900<br>-9	No
Kcal/portion	F1B_24c	N3	1-900<br>Missing	1-900<br>-9	No
Yes	F1B_24d	N1	Selected<br>Not selected	1<br>0	No

			Missing	-9	
Nr. of products	F1B_24e	N2	0-99<br>Missing	0-99<br>-9	No
Comments	F1B_24_C	A100	Missing	-9	No
Country specific product 25	F1B_25a	A50	Missing	-9	No
Gr./portion	F1B_25b	N3	1-900<br>Missing	1-900<br>-9	No
Kcal/portion	F1B_25c	N3	1-900<br>Missing	1-900<br>-9	No
Yes	F1B_25d	N1	Selected<br>Not selected<br>Missing	1<br>0<br>-9	No
Nr. of products	F1B_25e	N2	0-99<br>Missing	0-99<br>-9	No
Comments	F1B_25_C	A100	Missing	-9	No
Country specific product 26	F1B_26a	A50	Missing	-9	No
Gr./portion	F1B_26b	N3	1-900<br>Missing	1-900<br>-9	No
Kcal/portion	F1B_26c	N3	1-900<br>Missing	1-900<br>-9	No
Yes	F1B_26d	N1	Selected<br>Not selected<br>Missing	1<br>0<br>-9	No
Nr. of products	F1B_26e	N2	0-99<br>Missing	0-99<br>-9	No
Comments	F1B_26_C	A100	Missing	-9	No
Country specific product 27	F1B_27a	A50	Missing	-9	No
Gr./portion	F1B_27b	N3	1-900<br>Missing	1-900<br>-9	No
Kcal/portion	F1B_27c	N3	1-900<br>Missing	1-900<br>-9	No
Yes	F1B_27d	N1	Selected<br>Not selected<br>Missing	1<br>0<br>-9	No
Nr. of products	F1B_27e	N2	0-99<br>Missing	0-99<br>-9	No
Comments	F1B_27_C	A100	Missing	-9	No
Country specific product 28	F1B_28a	A50	Missing	-9	No
Gr./portion	F1B_28b	N3	1-900<br>Missing	1-900<br>-9	No
Kcal/portion	F1B_28c	N3	1-900<br>Missing	1-900<br>-9	No
Yes	F1B_28d	N1	Selected<br>Not selected<br>Missing	1<br>0<br>-9	No
Nr. of products	F1B_28e	N2	0-99<br>Missing	0-99<br>-9	No
Comments	F1B_28_C	A100	Missing	-9	No
Country specific product 29	F1B_29a	A50	Missing	-9	No
Gr./portion	F1B_29b	N3	1-900<br>Missing	1-900<br>-9	No
Kcal/portion	F1B_29c	N3	1-900<br>Missing	1-900<br>-9	No
Yes	F1B_29d	N1	Selected<br>Not selected<br>Missing	1<br>0<br>-9	No
Nr. of products	F1B_29e	N2	0-99<br>Missing	0-99<br>-9	No
Comments	F1B_29_C	A100	Missing	-9	No

Country specific product 30	F1B_30a	A50	Missing	-9	No
Gr./portion	F1B_30b	N3	1-900<br>Missing	1-900<br>-9	No
Kcal/portion	F1B_30c	N3	1-900<br>Missing	1-900<br>-9	No
Yes	F1B_30d	N1	Selected<br>Not selected<br>Missing	1<br>0<br>-9	No
Nr. of products	F1B_30e	N2	0-99<br>Missing	0-99<br>-9	No
Comments	F1B_30_C	A100	Missing	-9	No
Country specific product 31	F1B_31a	A50	Missing	-9	No
Gr./portion	F1B_31b	N3	1-900<br>Missing	1-900<br>-9	No
Kcal/portion	F1B_31c	N3	1-900<br>Missing	1-900<br>-9	No
Yes	F1B_31d	N1	Selected<br>Not selected<br>Missing	1<br>0<br>-9	No
Nr. of products	F1B_31e	N2	0-99<br>Missing	0-99<br>-9	No
Comments	F1B_31_C	A100	Missing	-9	No
Country specific product 32	F1B_32a	A50	Missing	-9	No
Gr./portion	F1B_32b	N3	1-900<br>Missing	1-900<br>-9	No
Kcal/portion	F1B_32c	N3	1-900<br>Missing	1-900<br>-9	No
Yes	F1B_32d	N1	Selected<br>Not selected<br>Missing	1<br>0<br>-9	No
Nr. of products	F1B_32e	N2	0-99<br>Missing	0-99<br>-9	No
Comments	F1B_32_C	A100	Missing	-9	No
**Fruits and vegetables**					
Country specific product 33	F1B_33a	A50	Missing	-9	No
Gr./portion	F1B_33b	N3	1-900<br>Missing	1-900<br>-9	No
Kcal/portion	F1B_33c	N3	1-900<br>Missing	1-900<br>-9	No
Yes	F1B_33d	N1	Selected<br>Not selected<br>Missing	1<br>0<br>-9	No
Nr. of products	F1B_33e	N2	0-99<br>Missing	0-99<br>-9	No
Comments	F1B_33_C	A100	Missing	-9	No
Country specific product 34	F1B_34a	A50	Missing	-9	No
Gr./portion	F1B_34b	N3	1-900<br>Missing	1-900<br>-9	No
Kcal/portion	F1B_34c	N3	1-900<br>Missing	1-900<br>-9	No
Yes	F1B_34d	N1	Selected<br>Not selected<br>Missing	1<br>0<br>-9	No
Nr. of products	F1B_34e	N2	0-99<br>Missing	0-99<br>-9	No
Comments	F1B_34_C	A100	Missing	-9	No
**Other**					
Country specific product 35	F1B_35a	A50	Missing	-9	No
Gr./portion	F1B_35b	N3	1-900	1-900	No

			Missing	-9	
Kcal/portion	F1B_35c	N3	1-900<br>Missing	1-900<br>-9	No
Yes	F1B_35d	N1	Selected<br>Not selected<br>Missing	1<br>0<br>-9	No
Nr. of products	F1B_35e	N2	0-99<br>Missing	0-99<br>-9	No
Comments	F1B_35_C	A100	Missing	-9	No
Country specific product 36	F1B_36a	A50	Missing	-9	No
Gr./portion	F1B_36b	N3	1-900<br>Missing	1-900<br>-9	No
Kcal/portion	F1B_36c	N3	1-900<br>Missing	1-900<br>-9	No
Yes	F1B_36d	N1	Selected<br>Not selected<br>Missing	1<br>0<br>-9	No
Nr. of products	F1B_36e	N2	0-99<br>Missing	0-99<br>-9	No
Comments	F1B_36_C	A100	Missing	-9	No
Country specific product 37	F1B_37a	A50	Missing	-9	No
Gr./portion	F1B_37b	N3	1-900<br>Missing	1-900<br>-9	No
Kcal/portion	F1B_37c	N3	1-900<br>Missing	1-900<br>-9	No
Yes	F1B_37d	N1	Selected<br>Not selected<br>Missing	1<br>0<br>-9	No
Nr. of products	F1B_37e	N2	0-99<br>Missing	0-99<br>-9	No
Comments	F1B_37_C	A100	Missing	-9	No
Country specific product 38	F1B_38a	A50	Missing	-9	No
Gr./portion	F1B_38b	N3	1-900<br>Missing	1-900<br>-9	No
Kcal/portion	F1B_38c	N3	1-900<br>Missing	1-900<br>-9	No
Yes	F1B_38d	N1	Selected<br>Not selected<br>Missing	1<br>0<br>-9	No
Nr. of products	F1B_38e	N2	0-99<br>Missing	0-99<br>-9	No
Comments	F1B_38_C	A100	Missing	-9	No
Country specific product 39	F1B_39a	A50	Missing	-9	No
Gr./portion	F1B_39b	N3	1-900<br>Missing	1-900<br>-9	No
Kcal/portion	F1B_39c	N3	1-900<br>Missing	1-900<br>-9	No
Yes	F1B_39d	N1	Selected<br>Not selected<br>Missing	1<br>0<br>-9	No
Nr. of products	F1B_39e	N2	0-99<br>Missing	0-99<br>-9	No
Comments	F1B_39_C	A100	Missing	-9	No
40. Photo of canteen/school shop/kiosk/restaurant attached?	F1B_40	N1	Yes<br>No<br>Missing	1<br>0<br>-9	No
Photo 1	F1B_40a	A50	(number of photo)<br>Not available (if F1B_40 = 0)	 -9	No
Photo 2	F1B_40b	A50	(number of photo)		No

			Not available (if F1B_40 = 0)	-9	
Photo 3	F1B_40c	A50	(number of photo)<br>Not available (if F1B_40 = 0)	 -9	No
41. Photo of wider area attached?	F1B_41	N1	Yes<br>No<br>Missing	1<br>0<br>-9	No
Photo 1	F1B_41a	A50	(number of photo)<br>Not available (if F1B_41 = 0)	 -9	No
Photo 2	F1B_41b	A50	(number of photo)<br>Not available (if F1B_41 = 0)	 -9	No
Photo 3	F1B_41c	A50	(number of photo)<br>Not available (if F1B_41 = 0)	 -9	No
42. Other observations/information	F1B_42	A100	Missing	-9	No
**Form 2A: Vending machines - interview**					
School ID	F2A_SCH	N3	001-998	001-998	Yes
Observer	F2A_OBS	A50	Missing	-9	No
Begin time	F2A_ST	date	00:00-24:00<br>Missing	00.00-24.00<br>-9	No
End time	F2A_ET	date	00:00-24:00<br>Missing	00.00-24.00<br>-9	No
1. The person who will be interviewed is the	F2A_1	N1	Vending machine administrator<br>Canteen/school shop/restaurant administrator<br>Principal/vice principal<br>Other school staff<br>Missing	1<br>2<br><br>3<br>4<br>-9	No
If other, who	F2A_1_C	A100	Missing	-9	No
2. Who decides what is offered in vending machines?	F2A_2	N1	The school management<br>The canteen/school shop/kiosk/ restaurant manager<br>The government<br>Parents<br>Pupils<br>Other<br>Missing	1<br>2<br><br>3<br>4<br>5<br>6<br>-9	No
If other, who	F2A_2_C	A100	Missing	-9	No
3. What are the three most sold food products in the vending machines?					
1.	F2A_3a	A100	Missing	-9	No
2.	F2A_3b	A100	Missing	-9	No
3.	F2A_3c	A100	Missing	-9	No
4. What are the three most sold drink items in the vending machines?					
1.	F2A_4a	A100	Missing	-9	No
2.	F2A_4b	A100	Missing	-9	No
3.	F2A_4c	A100	Missing	-9	No
5. How often are the vending machines refilled?					
Per week	F2A_5a	N1	1-7<br>Missing	1-7<br>-9	No
Per month	F2A_5b	N2	1-31<br>Missing	1-31<br>-9	No
6. Are there any restrictions on hours of operation/accessibility of vending machines?	F2A_6	N1	Yes<br>No<br>Missing	1<br>0<br>-9	No
If yes, explain	F2A_6_C	A100	Missing	-9	No
7. Are any vending machines owned	F2A_7	N1	Yes	1	No

and operated by the school?			No<br>Missing	0<br>-9	
8. Does the school/parents/pupils make profit on vending machines from product sales or as rent to the company/person who is running the vending machines?	F2A_8	N1	Yes<br>No<br>n/a<br>Missing	1<br>0<br>2<br>-9	No
If yes, how much and who	F2A_8_C	A100	Missing	-9	No
9. How does the school use that profit?	F2A_9	A100	Missing	-9	No
10. Other observations/information	F2A_10	A100	Missing	-9	No
**Form 2B: Vending machines**					
School ID	F2B_SCH	N3	001-998	001-998	Yes
Observer	F2B_OBS	A50	 Missing	Empty<br>-9	No
Begin time	F2B_ST	date	00:00-24:00<br>Missing	00.00-24.00<br>-9	No
End time	F2B_ET	date	00:00-24:00<br>Missing	00.00-24.00<br>-9	No
1. Indicate where the vending machine is located					
Vending machine 1	F2B_1a	A100	Missing	-9	No
Vending machine 2	F2B_1b	A100	Missing	-9	No
Vending machine 3	F2B_1c	A100	Missing	-9	No
2. Type of machine					
Vending machine 1	F2B_2a	A100	Missing	-9	No
Vending machine 2	F2B_2b	A100	Missing	-9	No
Vending machine 3	F2B_2c	A100	Missing	-9	No
3.Is the machine currently on and available to students or is it turned off?					
Vending machine 1	F2B_3a	N1	On<br>Off<br>Missing	1<br>0<br>-9	No
Vending machine 2	F2B_3b	N1	On<br>Off<br>Missing	1<br>0<br>-9	No
Vending machine 3	F2B_3c	N1	On<br>Off<br>Missing	1<br>0<br>-9	No
**Bread products**					
Country specific product 1	F2B_4a	A50	Missing	-9	No
Gr./portion	F2B_4b	N3	1-900<br>Missing	1-900<br>-9	No
Kcal/portion	F2B_4c	N3	1-900<br>Missing	1-900<br>-9	No
Yes	F2B_4d	N1	Selected<br>Not selected<br>Missing	1<br>0<br>-9	No
Nr. of products	F2B_4e	N2	0-99<br>Missing	0-99<br>-9	No
Comments	F2B_4_C	A100	Missing	-9	No
Country specific product 2	F2B_5a	A50	Missing	-9	No
Gr./portion	F2B_5b	N3	1-900<br>Missing	1-900<br>-9	No
Kcal/portion	F2B_5c	N3	1-900<br>Missing	1-900<br>-9	No
Yes	F2B_5d	N1	Selected	1	No

			Not selected<br>Missing	0<br>-9	
Nr. of products	F2B_5e	N2	0-99<br>Missing	0-99<br>-9	No
Comments	F2B_5_C	A100	Missing	-9	No
Country specific product 3	F2B_6a	A50	Missing	-9	No
Gr./portion	F2B_6b	N3	1-900<br>Missing	1-900<br>-9	No
Kcal/portion	F2B_6c	N3	1-900<br>Missing	1-900<br>-9	No
Yes	F2B_6d	N1	Selected<br>Not selected<br>Missing	1<br>0<br>-9	No
Nr. of products	F2B_6e	N2	0-99<br>Missing	0-99<br>-9	No
Comments	F2B_6_C	A100	Missing	-9	No
**Drinks**					
Country specific product 4	F2B_7a	A50	Missing	-9	No
ml/bottle, can, package	F2B_7b	N3	1-900<br>Missing	1-900<br>-9	No
Kcal/portion	F2B_7c	N3	1-900<br>Missing	1-900<br>-9	No
Yes	F2B_7d	N1	Selected<br>Not selected<br>Missing	1<br>0<br>-9	No
Nr. of products	F2B_7e	N2	0-99<br>Missing	0-99<br>-9	No
Comments	F2B_7_C	A100	Missing	-9	No
Country specific product 5	F2B_8a	A50	Missing	-9	No
ml/bottle, can, package	F2B_8b	N3	1-900<br>Missing	1-900<br>-9	No
Kcal/portion	F2B_8c	N3	1-900<br>Missing	1-900<br>-9	No
Yes	F2B_8d	N1	Selected<br>Not selected<br>Missing	1<br>0<br>-9	No
Nr. of products	F2B_8e	N2	0-99<br>Missing	0-99<br>-9	No
Comments	F2B_8_C	A100	Missing	-9	No
Country specific product 6	F2B_9a	A50	Missing	-9	No
ml/bottle, can, package	F2B_9b	N3	1-900<br>Missing	1-900<br>-9	No
Kcal/portion	F2B_9c	N3	1-900<br>Missing	1-900<br>-9	No
Yes	F2B_9d	N1	Selected<br>Not selected<br>Missing	1<br>0<br>-9	No
Nr. of products	F2B_9e	N2	0-99<br>Missing	0-99<br>-9	No
Comments	F2B_9_C	A100	Missing	-9	No
**Snacks/cakes/candies**					
Country specific product 7	F2B_10a	A50	Missing	-9	No
Gr./portion	F2B_10b	N3	1-900<br>Missing	1-900<br>-9	No
Kcal/portion	F2B_10c	N3	1-900<br>Missing	1-900<br>-9	No
Yes	F2B_10d	N1	Selected<br>Not selected<br>Missing	1<br>0<br>-9	No

Nr. of products	F2B_10e	N2	0-99<br>Missing	0-99<br>-9	No
Comments	F2B_10_C	A100	Missing	-9	No
Country specific product 8	F2B_11a	A50	Missing	-9	No
Gr./portion	F2B_11b	N3	1-900<br>Missing	1-900<br>-9	No
Kcal/portion	F2B_11c	N3	1-900<br>Missing	1-900<br>-9	No
Yes	F2B_11d	N1	Selected<br>Not selected<br>Missing	1<br>0<br>-9	No
Nr. of products	F2B_11e	N2	0-99<br>Missing	0-99<br>-9	No
Comments	F2B_11_C	A100	Missing	-9	No
**Other**					
Country specific product 9	F2B_12a	A50	Missing	-9	No
Gr./portion	F2B_12b	N3	1-900<br>Missing	1-900<br>-9	No
Kcal/portion	F2B_12c	N3	1-900<br>Missing	1-900<br>-9	No
Yes	F2B_12d	N1	Selected<br>Not selected<br>Missing	1<br>0<br>-9	No
Nr. of products	F2B_12e	N2	0-99<br>Missing	0-99<br>-9	No
Comments	F2B_12_C	A100	Missing	-9	No
Country specific product 10	F2B_13a	A50	Missing	-9	No
Gr./portion	F2B_13b	N3	1-900<br>Missing	1-900<br>-9	No
Kcal/portion	F2B_13c	N3	1-900<br>Missing	1-900<br>-9	No
Yes	F2B_13d	N1	Selected<br>Not selected<br>Missing	1<br>0<br>-9	No
Nr. of products	F2B_13e	N2	0-99<br>Missing	0-99<br>-9	No
Comments	F2B_13_C	A100	Missing	-9	No
11. Photo of vending machine(s) attached?	F2B_14	N1	Yes<br>No<br>Missing	1<br>0<br>-9	No
Photo 1	F2B_14a	A50	(number of photo)<br>Not available (if F2B_11 = 0)	-9	No
Photo 2	F2B_14b	A50	(number of photo)<br>Not available (if F2B_11 = 0)	-9	No
Photo 3	F2B_14c	A50	(number of photo)<br>Not available (if F2B_11 = 0)	-9	No
12. Photo of location attached?	F2B_15	N1	Yes<br>No<br>Missing	1<br>0<br>-9	No
Photo 1	F2B_15a	A50	(number of photo)<br>Not available (if F2B_12 = 0)	-9	No
Photo 2	F2B_15b	A50	(number of photo)<br>Not available (if F2B_12 = 0)	-9	No
Photo 3	F2B_15c	A50	(number of photo)<br>Not available (if F2B_12 = 0)	-9	No
**Form 3: Subscription programmes**					

School ID	F3_SCH	N3	001-998	001-998	Yes
Observer	F3_OBS	A50	Missing	-9	No
Begin time	F3_ST	date	00:00-24:00<br>Missing	00.00-24.00<br>-9	No
End time	F3_ET	date	00:00-24:00<br>Missing	00.00-24.00<br>-9	No
1. Name of program					
a. Program 1	F3_1a	A100	Missing	-9	No
b. Program 2	F3_1b	A100	Missing	-9	No
2. Products offered					
a. Program 1	F3_2a	A100	Missing	-9	No
b. Program 2	F3_2b	A100	Missing	-9	No
3. Offered by week?					
a. Program 1	F3_3a	N1	1 day/week<br>2 days/week<br>3 days/week<br>4 days/week<br>Everyday<br>Missing	1<br>2<br>3<br>4<br>5<br>-9	No
b. Program 2	F3_3b	N1	1 day/week<br>2 days/week<br>3 days/week<br>4 days/week<br>Everyday<br>Missing	1<br>2<br>3<br>4<br>5<br>-9	No
4. Season offered?					
a. Program 1	F3_4a	N1	All the year<br>Spring<br>Summer<br>Autumn<br>Winter<br>Missing	1<br>2<br>3<br>4<br>5<br>-9	No
b. Program 2	F3_4b	N1	All the year<br>Spring<br>Summer<br>Autumn<br>Winter<br>Missing	1<br>2<br>3<br>4<br>5<br>-9	No
5. Offered by whom?					
a. Program 1	F3_5a	N1	Government<br>Large/national food company<br>Small/local food company<br>Other<br>Missing	1<br>2<br>3<br>4<br>-9	No
Other, explain	F3_5a_C	A100	Missing	-9	No
b. Program 2	F3_5b	N1	Government<br>Large/national food company<br>Small/local food company<br>Other<br>Missing	1<br>2<br>3<br>4<br>-9	No
Other, explain	F3_5b_C	A100	Missing	-9	No
6. Paid by whom?					
a. Program 1	F3_6a	N1	By government<br>By the school<br>By parents<br>Other<br>Missing	1<br>2<br>3<br>4<br>-9	No
Other, explain	F3_6a_C	A100	Missing	-9	No
b. Program 2	F3_6b	N1	By government	1	No

			By the school<br>By parents<br>Other<br>Missing	2<br>3<br>4<br>-9	
Other, explain	F3_6b_C	A100	Missing	-9	No
7. How administrated in the school?					
a. Program 1	F3_7a	N1	By staff paid from school<br>Parents<br>Other<br>Missing	1<br>2<br>3<br>-9	No
Other, explain	F3_7a_C	A100	Missing	-9	No
b. Program 2	F3_7b	N1	By staff paid from school<br>Parents<br>Other<br>Missing	1<br>2<br>3<br>-9	No
Other, explain	F3_7b_C	A100	Missing	-9	No
8. For how long has the program been in place?					
a. Program 1	F3_8a	N1	Less than a year<br>1-2 years<br>3-4 years<br>5 years or more<br>Missing	0,5<br>1,5<br>3,5<br>5,5<br>-9	No
b. Program 2	F3_8b	N1	Less than a year<br>1-2 years<br>3-4 years<br>5 years or more<br>Missing	0,5<br>1,5<br>3,5<br>5,5<br>-9	No
9. Comments					
a. Program 1	F3_9a	A100	Missing	-9	No
b. Program 2	F3_9b	A100	Missing	-9	No
10. Photo of program 1 attached?	F3_10	N1	Yes<br>No<br>Missing	1<br>0<br>-9	No
Photo 1	F3_10a	A50	(number of photo)<br>Not available (if F3_10 = 0)	 -9	No
Photo 2	F3_10b	A50	(number of photo)<br>Not available (if F3_10 = 0)	 -9	No
Photo 3	F3_10c	A50	(number of photo)<br>Not available (if F3_10 = 0)	 -9	No
11. Photo of program 2 attached?	F3_11	N1	Yes<br>No<br>Missing	1<br>0<br>-9	No
Photo 1	F3_11a	A50	(number of photo)<br>Not available (if F3_11 = 0)	 -9	No
Photo 2	F3_11b	A50	(number of photo)<br>Not available (if F3_11 = 0)	 -9	No
Photo 3	F3_11c	A50	(number of photo)<br>Not available (if F3_11 = 0)	 -9	No
12. Other observations/information	F3_12	A100	Missing	-9	No
**Form 4: Food and drink commercial advertising**					
School ID	F4_SCH	N3	001-998	001-998	Yes
Observer	F4_OBS	A50	Missing	-9	No
Begin time	F4_ST	date	00:00-24:00<br>Missing	00.00-24.00<br>-9	No
End time	F4_ET	date	00:00-24:00<br>Missing	00.00-24.00<br>-9	No

1. Are there any food or beverage commercial advertisements in the following locations?					
In the canteen/school shop/kiosk/ restaurant	F4_1a	N1	Selected<br>Not selected<br>Missing	1<br>0<br>-9	No
On vending machines	F4_1b	N1	Selected<br>Not selected<br>Missing	1<br>0<br>-9	No
In the hallways	F4_1c	N1	Selected<br>Not selected<br>Missing	1<br>0<br>-9	No
In the classroom of 5th and 6th graders	F4_1d	N1	Selected<br>Not selected<br>Missing	1<br>0<br>-9	No
On school grounds, including the outside of school buildings, on playing fields, or other areas of the school	F4_1e	N1	Selected<br>Not selected<br>Missing	1<br>0<br>-9	No
Other	F4_1f	N1	Selected<br>Not selected<br>Missing	1<br>0<br>-9	No
Comments	F4_1_C	A100	Missing	-9	No
2. Please indicate the types of food advertised					
No food advertisements	F4_2a	N1	Selected<br>Not selected<br>Missing	1<br>0<br>-9	No
Fruits and vegetables	F4_2b	N1	Selected<br>Not selected<br>Missing	1<br>0<br>-9	No
Bread products	F4_2c	N1	Selected<br>Not selected<br>Missing	1<br>0<br>-9	No
Snacks, cakes, candies	F4_2d	N1	Selected<br>Not selected<br>Missing	1<br>0<br>-9	No
Other	F4_2e	N1	Selected<br>Not selected<br>Missing	1<br>0<br>-9	No
If other, explain	F4_2_C	A100	Missing	-9	No
3. Please indicate the types of beverages advertised					
No beverage advertisements	F4_3a	N1	Selected<br>Not selected<br>Missing	1<br>0<br>-9	No
100% fruit juice	F4_3b	N1	Selected<br>Not selected<br>Missing	1<br>0<br>-9	No
Sweetened drinks	F4_3c	N1	Selected<br>Not selected<br>Missing	1<br>0<br>-9	No
Diet drinks	F4_3d	N1	Selected<br>Not selected<br>Missing	1<br>0<br>-9	No

Milk	F4_3e	N1	Selected<br>Not selected<br>Missing	1<br>0<br>-9	No
Water	F4_3f	N1	Selected<br>Not selected<br>Missing	1<br>0<br>-9	No
Other	F4_3g	N1	Selected<br>Not selected<br>Missing	1<br>0<br>-9	No
If other, explain	F4_3_C	A100	Missing	-9	No
4. Photo of commercial advertisements attached?	F4_4	N1	Yes<br>No<br>Missing	1<br>0<br>-9	No
Photo 1	F4_4a	A50	(number of photo)<br>Not available (if F4_4 = 0)	 -9	No
Photo 2	F4_4b	A50	(number of photo)<br>Not available (if F4_4 = 0)	 -9	No
Photo 3	F4_4c	A50	(number of photo)<br>Not available (if F4_4 = 0)	 -9	No
5. Other observations/information	F4_5	A100	Missing	-9	No
**Form 5: Bicycle parking area**					
School ID	F5_SCH	N3	001-998	001-998	Yes
Observer	F5_OBS	A50	Missing	-9	No
Begin time	F5_ST	date	00:00-24:00<br>Missing	00.00-24.00<br>-9	No
End time	F5_ET	date	00:00-24:00<br>Missing	00.00-24.00<br>-9	No
1. What is the condition of the bicycle parking	F5_1	N1	1-7<br>Missing	1-7<br>-9	No
If less than 3 explain	F5_1_C	A100	Missing	-9	No
2. How is the bicycle parking secured?					
Fully covered	F5_2a	N1	Selected<br>Not selected<br>Missing	1<br>0<br>-9	No
Partly covered	F5_2b	N1	Selected<br>Not selected<br>Missing	1<br>0<br>-9	No
Supervised	F5_2c	N1	Selected<br>Not selected<br>Missing	1<br>0<br>-9	No
Doorkeeper	F5_2c1	N1	Selected<br>Not selected<br>Missing	1<br>0<br>-9	No
Camera	F5_2c2	N1	Selected<br>Not selected<br>Missing	1<br>0<br>-9	No
Reception	F5_2c3	N1	Selected<br>Not selected<br>Missing	1<br>0<br>-9	No
Other	F5_2c4	N1	Selected<br>Not selected<br>Missing	1<br>0<br>-9	No
If other, what?	F5_2c4_C	A100	Missing	-9	No
Surrounded by a fence	F5_2d	N1	Selected<br>Not selected	1<br>0	No

			Missing	-9	
3. Are there bicycle racks?	F5_3	N1	Yes<br>No<br>Missing	1<br>0<br>-9	No
4. How many bicycles can be placed in the racks?	F5_4	N3	0-500<br>Missing	0-500<br>-9	No
5. Is the bicycle parking full of bicycles?	F5_5	N1	More than full >100%<br>Full >75%<br>Mean 25-75%<br>Empty <25%<br>Missing	1<br>2<br>3<br>4<br>-9	No
Number of bikes	F5_5a	N3	0-500<br>Missing	 -9	No
6. Photo of bicycle parking attached?	F5_6	N1	Yes<br>No<br>Missing	1<br>0<br>-9	No
Photo 1	F5_6a	A50	(number of photo)<br>Not available (if F5_6 = 0)	 -9	No
Photo 2	F5_6b	A50	(number of photo)<br>Not available (if F5_6 = 0)	 -9	No
Photo 3	F5_6c	A50	(number of photo)<br>Not available (if F5_6 = 0)	 -9	No
7. Other observations/information	F5_7	A100	Missing	-9	No
**Form 6: Physical activity equipment (loose) for recess**					
School ID	F6_SCH	N3	001-998	001-998	Yes
Observer	F6_OBS	A50	Missing	-9	No
Begin time	F6_ST	date	00:00-24:00<br>Missing	00.00-24.00<br>-9	No
End time	F6_ET	date	00:00-24:00<br>Missing	00.00-24.00<br>-9	No
1. How often is the following equipment used by 5th and 6th graders during recess?	F6_1	N1	Every recess<br>Once a day<br>A few times per week<br>Less than once a week<br>Other<br>Missing	1<br>2<br>3<br>4<br>5<br>-9	No
If other, specify when and why	F6_1_C	A100	Missing	-9	No
2. Does the equipment look inviting to play with?	F6_2	N1	1-7<br>Missing	1-7<br>-9	No
If less than 3, explain	F6_2_C	A100	Missing	-9	No
**Equipment**					
3. Footballs	F6_3a	N1	Yes<br>No<br>Missing	1<br>0<br>-9	No
Number of footballs	F6_3b	N2	0-99<br>Not available (if F6_3a = 0)<br>Missing	0-99<br>Empty<br>-9	No
Comments	F6_3_C	A100	Not available (if F6_3a = 0)<br>Missing	Empty<br>-9	No
4. Basketballs	F6_4a	N1	Yes<br>No<br>Missing	1<br>0<br>-9	No
Number of basketballs	F6_4b	N2	0-99<br>Not available (if F6_4a = 0)<br>Missing	0-99<br>Empty<br>-9	No
Comments	F6_4_C	A100	Not available (if F6_4a = 0)<br>Missing	Empty<br>-9	No

5. Volleyballs	F6_5a	N1	Yes<br>No<br>Missing	1<br>0<br>-9	No
Number of volleyballs	F6_5b	N2	0-99<br>Not available (if F6_5a = 0)<br>Missing	0-99<br>Empty<br>-9	No
Comments	F6_5_C	A100	Not available (if F6_5a = 0)<br>Missing	Empty<br>-9	No
6. Other balls	F6_6a	N1	Yes<br>No<br>Missing	1<br>0<br>-9	No
Number of other balls	F6_6b	N2	0-99<br>Not available (if F6_6a = 0)<br>Missing	0-99<br>Empty<br>-9	No
Comments	F6_6_C	A100	Not available (if F6_6a = 0)<br>Missing	Empty<br>-9	No
7. Rackets	F6_7a	N1	Yes<br>No<br>Missing	1<br>0<br>-9	No
Number of rackets	F6_7b	N2	0-99<br>Not available (if F6_7a = 0)<br>Missing	0-99<br>Empty<br>-9	No
Comments	F6_7_C	A100	Not available (if F6_7a = 0)<br>Missing	Empty<br>-9	No
8. Bandy/hockey sticks	F6_8a	N1	Yes<br>No<br>Missing	1<br>0<br>-9	No
Number of bandy/hockey sticks	F6_8b	N2	0-99<br>Not available (if F6_8a = 0)<br>Missing	0-99<br>Empty<br>-9	No
Comments	F6_8_C	A100	Not available (if F6_8a = 0)<br>Missing	Empty<br>-9	No
9. Bats (i.e. baseball)	F6_9a	N1	Yes<br>No<br>Missing	1<br>0<br>-9	No
Number of bats	F6_9b	N2	0-99<br>Not available (if F6_9a = 0)<br>Missing	0-99<br>Empty<br>-9	No
Comments	F6_9_C	A100	Not available (if F6_9a = 0)<br>Missing	Empty<br>-9	No
10. Skipping ropes	F6_10a	N1	Yes<br>No<br>Missing	1<br>0<br>-9	No
Number of skipping ropes	F6_10b	N2	0-99<br>Not available (if F6_10a = 0)<br>Missing	0-99<br>Empty<br>-9	No
Comments	F6_10_C	A100	Not available (if F6_10a = 0)<br>Missing	Empty<br>-9	No
11. Jump bands (elastics)	F6_11a	N1	Yes<br>No<br>Missing	1<br>0<br>-9	No
Number of jump bands	F6_11b	N2	0-99<br>Not available (if F6_11a = 0)<br>Missing	0-99<br>Empty<br>-9	No
Comments	F6_11_C	A100	Not available (if F6_11a = 0)<br>Missing	Empty<br>-9	No
12. Frisbees	F6_12a	N1	Yes<br>No<br>Missing	1<br>0<br>-9	No

Number of frisbees	F6_12b	N2	0-99<br>Not available (if F6_12a = 0)<br>Missing	0-99<br>Empty<br>-9	No
Comments	F6_12_C	A100	Not available (if F6_12a = 0)<br>Missing	Empty<br>-9	No
13. Other equipment 1	F6_13a	N1	Yes<br>No<br>Missing	1<br>0<br>-9	No
Number of other equipment 1	F6_13b	N2	0-99<br>Not available (if F6_13a = 0)<br>Missing	0-99<br>Empty<br>-9	No
Comments	F6_13_C	A100	Not available (if F6_13a = 0)<br>Missing	Empty<br>-9	No
14. Other equipment 2	F6_14a	N1	Yes<br>No<br>Missing	1<br>0<br>-9	No
Number of other equipment 2	F6_14b	N2	0-99<br>Not available (if F6_14a = 0)<br>Missing	0-99<br>Empty<br>-9	No
Comments	F6_14_C	A100	Not available (if F6_14a = 0)<br>Missing	Empty<br>-9	No
15. Other equipment 3	F6_15a	N1	Yes<br>No<br>Missing	1<br>0<br>-9	No
Number of other equipment 3	F6_15b	N2	0-99<br>Not available (if F6_15a = 0)<br>Missing	0-99<br>Empty<br>-9	No
Comments	F6_15_C	A100	Not available (if F6_15a = 0)<br>Missing	Empty<br>-9	No
16. Photo of room/box for keeping the equipment attached?	F6_16	N1	Yes<br>No<br>Missing	1<br>0<br>-9	No
Photo 1	F6_16a	A50	(number of photo)<br>Not available (if F6_16)	 -9	No
Photo 2	F6_16b	A50	(number of photo)<br>Not available (if F6_16 = 0)	 -9	No
Photo 3	F6_16c	A50	(number of photo)<br>Not available (if F6_16 = 0)	 -9	No
17. Photo of equipment attached?	F6_17	N1	Yes<br>No<br>Missing	1<br>0<br>-9	No
Photo 1	F6_17a	A50	(number of photo)<br>Not available (if F6_17 = 0)	 -9	No
Photo 2	F6_17b	A50	(number of photo)<br>Not available (if F6_17 = 0)	 -9	No
Photo 3	F6_17c	A50	(number of photo)<br>Not available (if F6_17 = 0)	 -9	No
18. Other observations/information	F6_18	A100	Missing	-9	No
**Form 7: Indoor sport facilities**					
School ID	F7_SCH	N3	001-998	001-998	Yes
Observer	F7_OBS	A50	Missing	-9	No
Begin time	F7_ST	date	00:00-24:00<br>Missing	00.00-24.00<br>-9	No
End time	F7_ET	date	00:00-24:00<br>Missing	00.00-24.00<br>-9	No
1. Are the indoor facilities open to pupils at other times than during physical education?	F7_1	N1	Yes<br>No<br>Missing	1<br>0<br>-9	No

Comments	F7_1_C	A100	Missing	-9	No
**Facilities**					
2. Gym	F7_2a	N1	Yes<br>No<br>Missing	1<br>0<br>-9	No
Number of gyms	F7_2b	N1	0-9<br>Not available (if F7_2a = 0)<br>Missing	0-9<br>Empty<br>-9	No
Size of gym (m²)	F7_2c	N4	10-5000<br>Not available (if F7_2a = 0)<br>Missing	100-5000<br>Empty<br>-9	No
Condition of gym	F7_2d	A100	Not available (if F7_2a = 0)<br>Missing	Empty<br>-9	No
3. Swimming pool	F7_3a	N1	Yes<br>No<br>Missing	1<br>0<br>-9	No
Number of swimming pools	F7_3b	N1	0-9<br>Not available (if F7_3a = 0)<br>Missing	0-9<br>Empty<br>-9	No
Size of swimming pool (m²)	F7_3c	N4	10-5000<br>Not available (if F7_3a = 0)<br>Missing	100-5000<br>Empty<br>-9	No
Condition of swimming pool	F7_3d	A100	Not available (if F7_2a = 0)<br>Missing	Empty<br>-9	No
4. Weight training facilities	F7_4a	N1	Yes<br>No<br>Missing	1<br>0<br>-9	No
Number of weight training facilities	F7_4b	N1	0-9<br>Not available (if F7_4a = 0)<br>Missing	0-9<br>Empty<br>-9	No
Size of weight training Facilities (m²)	F7_4c	N4	10-5000<br>Not available (if F7_4a = 0)<br>Missing	100-5000<br>Empty<br>-9	No
Condition of weight training facilities	F7_4d	A100	Not available (if F7_4a = 0)<br>Missing	Empty<br>-9	No
5. Gender specific changing rooms	F7_5a	N1	Yes<br>No<br>Missing	1<br>0<br>-9	No
Number of gender specific changing rooms	F7_5b	N1	0-9<br>Not available (if F7_5a = 0)<br>Missing	0-9<br>Empty<br>-9	No
Size of gender specific changing rooms (m²)	F7_5c	N4	10-5000<br>Not available (if F7_5a = 0)<br>Missing	100-5000<br>Empty<br>-9	No
Condition of gender specific changing rooms	F7_5d	A100	Not available (if F7_5a = 0)<br>Missing	Empty<br>-9	No
6. Shower facilities	F7_6a	N1	Yes<br>No<br>Missing	1<br>0<br>-9	No
Number of shower facilities	F7_6b	N1	0-9<br>Not available (if F7_6a = 0)<br>Missing	0-9<br>Empty<br>-9	No
Size of shower facilities (m²)	F7_6c	N4	10-5000<br>Not available (if F7_6a = 0)<br>Missing	100-5000<br>Empty<br>-9	No
Condition of shower facilities	F7_6d	A100	Not available (if F7_6a = 0)<br>Missing	Empty<br>-9	No
7. Other facilities 1	F7_7a	N1	Yes<br>No	1<br>0	No

			Missing	-9	
Specify other facilities 1	F7_7a_C	A100	Not available (if F7_7a = 0)<br>Missing	Empty<br>-9	No
Number of other facilities 1	F7_7b	N1	0-9<br>Not available (if F7_7a = 0)<br>Missing	0-9<br>Empty<br>-9	No
Size of other facilities 1 (m²)	F7_7c	N4	10-5000<br>Not available (if F7_7a = 0)<br>Missing	100-5000<br>Empty<br>-9	No
Condition of other facilities 1	F7_7d	A50	Not available (if F7_7a = 0)<br>Missing	Empty<br>-9	No
8. Other facilities 2	F7_8a	N1	Yes<br>No<br>Missing	1<br>0<br>-9	No
Specify other facilities 2	F7_8a_C	A100	Not available (if F7_8a = 0)<br>Missing	Empty<br>-9	No
Number of other facilities 2	F7_8b	N1	0-9<br>Not available (if F7_8a = 0)<br>Missing	0-9<br>Empty<br>-9	No
Size of other facilities 2 (m²)	F7_8c	N4	10-5000<br>Not available (if F7_8a = 0)<br>Missing	100-5000<br>Empty<br>-9	No
Condition of other facilities 2	F7_8d	A50	Not available (if F7_8a = 0)<br>Missing	Empty<br>-9	No
9. Photo of indoor gym attached	F7_9	N1	Yes<br>No<br>Missing	1<br>0<br>-9	No
Photo 1	F7_9a	A50	(number of photo)<br>Not available (if F7_9 = 0)	-9	No
Photo 2	F7_9b	A50	(number of photo)<br>Not available (if F7_9 = 0)	-9	No
Photo 3	F7_9c	A50	(number of photo)<br>Not available (if F7_9 = 0)	-9	No
10. Photo of equipment for indoor gym attached?	F7_10	N1	Yes<br>No<br>Missing	1<br>0<br>-9	No
Photo 1	F7_10a	A50	(number of photo)<br>Not available (if F7_10 = 0)	-9	No
Photo 2	F7_10b	A50	(number of photo)<br>Not available (if F7_10 = 0)	-9	No
Photo 3	F7_10c	A50	(number of photo)<br>Not available (if F7_10 = 0)	-9	No
11. Other observations/information	F7_11	A100	Missing	-9	No
**Form 8: Outdoor sport facilities**					
School ID	F8_SCH	N3	001-998	001-998	Yes
Observer	F8_OBS	A50	Missing	-9	No
Begin time	F8_ST	date	00:00-24:00<br>Missing	00.00-24.00<br>-9	No
End time	F8_ET	date	00:00-24:00<br>Missing	00.00-24.00<br>-9	No
1. Are there parts of the school grounds designated to specific grade levels?	F8_1	N1	Yes<br>No<br>Missing	1<br>0<br>-9	No
Comments	F8_1_C	A100	Missing	-9	No
**Facilities**					
2. Football field	F8_2a	N1	Yes<br>No<br>Missing	1<br>0<br>-9	No

Number of football fields	F8_2b	N1	0-9<br>Not available (if F8_2a = 0)<br>Missing	0-9<br>Empty<br>-9	No
Size of football field (m²)	F8_2c	N4	10-5000<br>Not available (if F8_2a = 0)<br>Missing	100-5000<br>Empty<br>-9	No
Condition of football field	F8_2d	A50	Not available (if F8_2a = 0)<br>Missing	Empty<br>-9	No
3. Basketball field	F8_3a	N1	Yes<br>No<br>Missing	1<br>0<br>-9	No
Number of basketball fields	F8_3b	N1	0-9<br>Not available (if F8_3a = 0)<br>Missing	0-9<br>Empty<br>-9	No
Size of basketball field (m²)	F8_3c	N4	10-5000<br>Not available (if F8_3a = 0)<br>Missing	100-5000<br>Empty<br>-9	No
Condition of basketball field	F8_3d	A50	Not available (if F8_3a = 0)<br>Missing	Empty<br>-9	No
4. Volleyball/badminton/tennis fields with net	F8_4a	N1	Yes<br>No<br>Missing	1<br>0<br>-9	No
Number of volleyball/badminton/tennis fields	F8_4b	N1	0-9<br>Not available (if F8_4a = 0)<br>Missing	0-9<br>Empty<br>-9	No
Size of volleyball/badminton/tennis fields (m²)	F8_4c	N4	10-5000<br>Not available (if F8_4a = 0)<br>Missing	100-5000<br>Empty<br>-9	No
Condition of volleyball/badminton/tennis fields	F8_4d	A50	Not available (if F8_4a = 0)<br>Missing	Empty<br>-9	No
5. Other fields for ballgames	F8_5a	N1	Yes<br>No<br>Missing	1<br>0<br>-9	No
Specify other fields	F8_5a_C	A100	Not available (if F8_5a = 0)<br>Missing	Empty<br>-9	No
Number of other fields	F8_5b	N1	0-9<br>Not available (if F8_5a = 0)<br>Missing	0-9<br>Empty<br>-9	No
Size of other fields (m²)	F8_5c	N4	10-5000<br>Not available (if F8_5a = 0)<br>Missing	100-5000<br>Empty<br>-9	No
Condition of other fields	F8_5d	A50	Not available (if F8_5a = 0)<br>Missing	Empty<br>-9	No
6. Paint/marks for other games	F8_6a	N1	Yes<br>No<br>Missing	1<br>0<br>-9	No
Number of paint/marks for other games	F8_6b	N1	0-9<br>Not available (if F8_6a = 0)<br>Missing	0-9<br>Empty<br>-9	No
Size of paint/marks for other Games (m²)	F8_6c	N4	10-5000<br>Not available (if F8_6a = 0)<br>Missing	100-5000<br>Empty<br>-9	No
Condition of paint/marks for other games	F8_6d	A50	Not available (if F8_6a = 0)<br>Missing	Empty<br>-9	No
7. Play area with swings, slides etc	F8_7a	N1	Yes<br>No<br>Missing	1<br>0<br>-9	No
Number of play areas	F8_7b	N1	0-9	0-9	No

			Not available (if F8_7a = 0)<br>Missing	Empty<br>-9	
Size of play areas (m²)	F8_7c	N4	10-5000<br>Not available (if F8_7a = 0)<br>Missing	100-5000<br>Empty<br>-9	No
Condition of play areas	F8_7d	A50	Not available (if F8_7a = 0)<br>Missing	Empty<br>-9	No
8. Table tennis	F8_8a	N1	Yes<br>No<br>Missing	1<br>0<br>-9	No
Number of table tennis facilities	F8_8b	N1	0-9<br>Not available (if F8_8a = 0)<br>Missing	0-9<br>Empty<br>-9	No
Size of table tennis facilities (m²)	F8_8c	N4	10-5000<br>Not available (if F8_8a = 0)<br>Missing	100-5000<br>Empty<br>-9	No
Condition of table tennis facilities	F8_8d	A50	Not available (if F7_2a = 0)<br>Missing	Empty<br>-9	No
9. Track and field (running, jumping, throwing)	F8_9a	N1	Yes<br>No<br>Missing	1<br>0<br>-9	No
Number of track and field	F8_9b	N1	0-9<br>Not available (if F8_9a = 0)<br>Missing	0-9<br>Empty<br>-9	No
Size of track and field (m²)	F8_9c	N4	10-5000<br>Not available (if F8_9a = 0)<br>Missing	100-5000<br>Empty<br>-9	No
Condition of track and field	F8_9d	A50	Not available (if F8_9a = 0)<br>Missing	Empty<br>-9	No
10. Obstacle course/jungle path	F8_10a	N1	Yes<br>No<br>Missing	1<br>0<br>-9	No
Number of obstacle courses	F8_10b	N1	0-9<br>Not available (if F8_10a = 0)<br>Missing	0-9<br>Empty<br>-9	No
Size of obstacle course (m²)	F8_10c	N4	10-5000<br>Not available (if F8_10a = 0)<br>Missing	100-5000<br>Empty<br>-9	No
Condition of obstacle course	F8_10d	A50	Not available (if F8_10a = 0)<br>Missing	Empty<br>-9	No
11. Climbing	F8_11a	N1	Yes<br>No<br>Missing	1<br>0<br>-9	No
Number of climbing facilities	F8_11b	N1	0-9<br>Not available (if F8_11a = 0)<br>Missing	0-9<br>Empty<br>-9	No
Size of climbing facilities (m²)	F8_11c	N4	10-5000<br>Not available (if F8_11a = 0)<br>Missing	100-5000<br>Empty<br>-9	No
Condition of climbing facilities	F8_11d	A50	Not available (if F8_11a = 0)<br>Missing	Empty<br>-9	No
12. Skateboard area	F8_12a	N1	Yes<br>No<br>Missing	1<br>0<br>-9	No
Number of skateboard areas	F8_12b	N1	0-9<br>Not available (if F8_12a = 0)<br>Missing	0-9<br>Empty<br>-9	No
Size of skateboard area (m²)	F8_12c	N4	10-5000<br>Not available (if F8_12a = 0)	100-5000<br>Empty	No

			Missing	-9	
Condition of skateboard area	F8_12d	A50	Not available (if F8_12a = 0)<br>Missing	Empty<br>-9	No
13. Other facilities 1	F8_13a	N1	Yes<br>No<br>Missing	1<br>0<br>-9	No
Specify other facilities 1	F8_13a_C	A100	Not available (if F8_13a = 0)<br>Missing	Empty<br>-9	No
Number of other facilities 1	F8_13b	N1	0-9<br>Not available (if F8_13a = 0)<br>Missing	0-9<br>Empty<br>-9	No
Size of other facilities 1 (m²)	F8_13c	N4	10-5000<br>Not available (if F8_13a = 0)<br>Missing	100-5000<br>Empty<br>-9	No
Condition of other facilities 1	F8_13d	A50	Not available (if F8_13a = 0)<br>Missing	Empty<br>-9	No
14. Other facilities 2	F8_14a	N1	Yes<br>No<br>Missing	1<br>0<br>-9	No
Specify other facilities 2	F8_14a_C	A100	Not available (if F8_14a = 0)<br>Missing	Empty<br>-9	No
Number of other facilities 2	F8_14b	N1	0-9<br>Not available (if F8_14a = 0)<br>Missing	0-9<br>Empty<br>-9	No
Size of other facilities 2 (m²)	F8_14c	N4	10-5000<br>Not available (if F8_14a = 0)<br>Missing	100-5000<br>Empty<br>-9	No
Condition of other facilities 2	F8_14d	A50	Not available (if F8_14a = 0)<br>Missing	Empty<br>-9	No
15. Other facilities 3	F8_15a	N1	Yes<br>No<br>Missing	1<br>0<br>-9	No
Specify other facilities 3	F8_15a_C	A100	Not available (if F8_15a = 0)<br>Missing	Empty<br>-9	No
Number of other facilities 3	F8_15b	N1	0-9<br>Not available (if F8_15a = 0)<br>Missing	0-9<br>Empty<br>-9	No
Size of other facilities 3 (m²)	F8_15c	N4	10-5000<br>Not available (if F8_15a = 0)<br>Missing	100-5000<br>Empty<br>-9	No
Condition of other facilities 3	F8_13d	A50	Not available (if F8_15a = 0)<br>Missing	Empty<br>-9	No
16. Photo of outdoor sport facilities attached?	F8_16	N1	Yes<br>No<br>Missing	1<br>0<br>-9	No
Photo 1	F8_16a	A50	(number of photo)<br>Not available (if F8_16 = 0)	-9	No
Photo 2	F8_16b	A50	(number of photo)<br>Not available (if F8_16 = 0)	-9	No
Photo 3	F8_16c	A50	(number of photo)<br>Not available (if F8_16 = 0)	-9	No
17. Other observations/information	F8_17	A100	Missing	-9	No
**Form 9: Other information about the school outdoor area**					
School ID	F9_SCH	N3	001-998	001-998	Yes
Observer	F9_OBS	A50	Missing	-9	No
Begin time	F9_ST	date	00:00-24:00<br>Missing	00.00-24.00<br>-9	No

End time	F9_ET	date	00:00-24:00<br>Missing	00.00-24.00<br>-9	No
1. What kinds of surfaces are present?					
Asphalt/paved	F89_1a	N1	Selected<br>Not selected<br>Missing	1<br>0<br>-9	No
Grass	F9_1b	N1	Selected<br>Not selected<br>Missing	1<br>0<br>-9	No
Gravel	F9_1c	N1	Selected<br>Not selected<br>Missing	1<br>0<br>-9	No
Sand	F9_1d	N1	Selected<br>Not selected<br>Missing	1<br>0<br>-9	No
Rocks	F9_1e	N1	Selected<br>Not selected<br>Missing	1<br>0<br>-9	No
Nature/woods	F9_1f	N1	Selected<br>Not selected<br>Missing	1<br>0<br>-9	No
Other	F9_1g	N1	Selected<br>Not selected<br>Missing	1<br>0<br>-9	No
If other, explain	F9_1_C	A100	Missing	-9	No
2. How is the topography?					
Flat (mostly)	F9_2a	N1	Selected<br>Not selected<br>Missing	1<br>0<br>-9	No
Hilly (mostly)	F9_2b	N1	Selected<br>Not selected<br>Missing	1<br>0<br>-9	No
Combination of both	F9_2c	N1	Selected<br>Not selected<br>Missing	1<br>0<br>-9	No
Other	F9_2d	N1	Selected<br>Not selected<br>Missing	1<br>0<br>-9	No
If other, explain	F9_2_C	A100	Missing	-9	No
3. How is the school's outdoor area separated from the neighbourhood?					
Fence/hedge	F9_3a	N1	Selected<br>Not selected<br>Missing	1<br>0<br>-9	No
No clear boundaries	F9_3b	N1	Selected<br>Not selected<br>Missing	1<br>0<br>-9	No
Other	F9_3c	N1	Selected<br>Not selected<br>Missing	1<br>0<br>-9	No
If other, explain	F9_3_C	A100	Missing	-9	No
4. Are there other things present?					
Plants/trees	F9_4a	N1	Selected<br>Not selected<br>Missing	1<br>0<br>-9	No
Benches (and tables)	F9_4b	N1	Selected<br>Not selected<br>Missing	1<br>0<br>-9	No

Drinking fountains	F9_4c	N1	Selected<br>Not selected<br>Missing	1<br>0<br>-9	No
Other	F9_4d	N1	Selected<br>Not selected<br>Missing	1<br>0<br>-9	No
If other, explain	F9_4_C	A100	Missing	-9	No
5. General appearance of the outdoor area	F9_5	N1	1-7<br>Missing	1-7<br>-9	No
If less than 3, explain	F9_5_C	A100	Missing	-9	No
6. Photo of a physical activity promoting characteristic attached?	F9_6	N1	Yes<br>No<br>Missing	1<br>0<br>-9	No
Photo 1	F9_6a	A50	(number of photo)<br>Not available (if F9_6 = 0)	 -9	No
Photo 2	F9_6b	A50	(number of photo)<br>Not available (if F9_6 = 0)	 -9	No
Photo 3	F9_6c	A50	(number of photo)<br>Not available (if F9_6 = 0)	 -9	No
7. Photo of barriers to physical activity attached?	F9_7	N1	Yes<br>No<br>Missing	1<br>0<br>-9	No
Photo 1	F9_7a	A50	(number of photo)<br>Not available (if F9_7 = 0)	 -9	No
Photo 2	F9_7b	A50	(number of photo)<br>Not available (if F9_7 = 0)	 -9	No
Photo 3	F9_7c	A50	(number of photo)<br>Not available (if F9_7 = 0)	 -9	No
8. Other observations/information	F9_8	A100	Missing	-9	No

Codebook ENERGY School management questionnaire

Section A: Some questions about yourself and your school					
**Question**	**Variable name**	**Type**	**Value**	**Code**	**Compulsory**
A1. Please write the name of the school	A1	A50	Missing	-9	Yes
A2a What is your position at the school	A2_a	N1	Headmaster<br>Vice headmaster<br>Other *(please specify)*<br>Missing	1<br>2<br>3<br>-9	No
Specification Other	A2_a_C	A50	Not available (if A2_a = 1 or 2)<br>Missing	-8<br>-9	No
A2b. For how long have you had this position in this school? (number of years)	A2_b	N2	0-99<br>Missing	0-99<br>-9	No
A3. What is your gender?	A3	N1	Male<br>Female<br>Missing	1<br>0<br>-9	No
A4. Is this school…?	A4	N1	Public<br>Private<br>Other *(Please specify)*<br>Missing	1<br>2<br>3<br>-9	
Specification Other	A4_C	A50	Not available (if A4= 1 or 2)<br>Missing	-9	No
A5. What is the total number of pupils in this school?					
Number of boys	A5_a	N4	0-9999<br>Missing	0-9999<br>-9	No
Number of girls	A5_b	N4	0-9999<br>Missing	0-9999<br>-9	No
A6a. What is the grade levels housed in this school?					
From	A6_a_from	N2	0-50<br>Missing	0-50<br>-9	No
To	A6_a_to	N2	0-50<br>Missing	0-50<br>-9	No
A6b. What is the age–range of the pupils housed in this school?					
	A6_b_from	N2	0-50<br>Missing	0-50<br>-9	No
	A6_b_to	N2	0-50<br>Missing	0-50<br>-9	No
A7. Approximately how many of the pupils in the school come from (live in) families with few economic resources (low income families, families who receive transfer incomes)? (Please estimate)	A7	N1	Less than 5 % of the pupils<br>5-9% of the pupils<br>10-19%<br>20-29%<br>30-39%<br>40-49%<br>50-74%<br>More than 75% of the pupils<br>Missing	1<br>2<br>3<br>4<br>5<br>6<br>7<br>8<br>-9	No
A8. Approximately how many of the pupils in the school come from ethnic minorities? (Please estimate)	A8	N1	Less than 5 % of the pupils<br>5-9% of the pupils<br>10-19%<br>20-29%<br>30-39%<br>40-49%<br>50-74%<br>More than 75% of the pupils	1<br>2<br>3<br>4<br>5<br>6<br>7<br>8	No

			Missing	-9	
A9. What is the usual start and end time (i.e. 08:00-14:15) of the school days in a regular week for 5-6th graders in this school?					
Monday start 5th grade	A9_Mon_5_start	Date	00:00-24:00<br>Missing	00.00-24.00<br>-9:00	No
Monday end 5th grade	A9_Mon_5_end	Date	00:00-24:00<br>Missing	00.00-24.00<br>-9:00	No
Tuesday start 5th grade	A9_Tues_5_start	Date	00:00-24:00<br>Missing	00.00-24.00<br>-9:00	No
Tuesday end 5th grade	A9_Tues_5_end	Date	00:00-24:00<br>Missing	00.00-24.00<br>-9:00	No
Wednesday start 5th grade	A9_Wed_5_start	Date	00:00-24:00<br>Missing	00.00-24.00<br>-9:00	No
Wednesday end 5th grade	A9_Wed_5_end	Date	00:00-24:00<br>Missing	00.00-24.00<br>-9:00	No
Thursday start 5th grade	A9_Thur_5_start	Date	00:00-24:00<br>Missing	00.00-24.00<br>-9:00	No
Thursday end 5th grade	A9_Thur_5_end	Date	00:00-24:00<br>Missing	00.00-24.00<br>-9:00	No
Friday start 5th grade	A9_Fri_5_start	Date	00:00-24:00<br>Missing	00.00-24.00<br>-9:00	No
Friday end 5th grade	A9_Fri_5_end	Date	00:00-24:00<br>Missing	00.00-24.00<br>-9:00	No
Monday start 6th grade	A9_Mon_6_start	Date	00:00-24:00<br>Missing	00.00-24.00<br>-9:00	No
Monday end 6th grade	A9_Mon_6_end	Date	00:00-24:00<br>Missing	00.00-24.00<br>-9:00	No
Tuesday start 6th grade	A9_Tues_6_start	Date	00:00-24:00<br>Missing	00.00-24.00<br>-9:00	No
Tuesday end 6th grade	A9_Tues_6_end	Date	00:00-24:00<br>Missing	00.00-24.00<br>-9:00	No
Wednesday start 6th grade	A9_Wed_6_start	Date	00:00-24:00<br>Missing	00.00-24.00<br>-9:00	No
Wednesday end 6th grade	A9_Wed_6_end	Date	00:00-24:00<br>Missing	00.00-24.00<br>-9:00	No
Thursday start 6th grade	A9_Thur_6_start	Date	00:00-24:00<br>Missing	00.00-24.00<br>-9:00	No
Thursday end 6th grade	A9_Thur_6_end	Date	00:00-24:00<br>Missing	00.00-24.00<br>-9:00	No
Friday start 6th grade	A9_Fri_6_start	Date	00:00-24:00<br>Missing	00.00-24.00<br>-9:00	No
Friday end 6th grade	A9_Fri_6_end	Date	00:00-24:00<br>Missing	00.00-24.00<br>-9:00	No

Section B: Opportunities to eat/drink and be physically active

**Question**	**Variable name**	**Type**	**Value**	**Code**	**Compulsory**
B10. How do the majority of the 5th-6th graders in your school get to/from school?	B10	N1	By motorised transportation<br>By walking/biking<br>About 50/50 of each of the above<br>Missing	1<br>2<br>3<br>-9	No
B11. Is it generally regarded as safe to walk or bike to your school?	B11	N1	No<br>Yes<br>Missing	0<br>1<br>-9	No
B12. Are there scheduled times to eat main meals or a “snack” for 5-6th graders?	B12	N1	No<br>Yes (please fill in table below)<br>Missing	0<br>1<br>-9	No

Name of eating occasion 1	B12_occ1	A50	Not available (if B12= 0)<br>Missing	-8<br>-9	No
Start time occasion1	B12_occ1_start	Date	00:00-24:00<br>Not available (if B12= 0)<br>Missing	00.00-24.00<br>-8<br>-9	No
End time occasion 1	B12_occ1_end	Date	00:00-24:00<br>Not available (if B12= 0)<br>Missing	00.00-24.00<br>-8<br>-9	No
Name of eating occasion 2	B12_occ2	A50	Not available (if B12= 0)<br>Missing	-8<br>-9	No
Start time occasion 2	B12_occ2_start	Date	00:00-24:00<br>Not available (if B12= 0)<br>Missing	00.00-24.00<br>-8<br>-9	No
End time occasion 2	B12_occ2_end	Date	00:00-24:00<br>Not available (if B12= 0)<br>Missing	00.00-24.00<br>-8<br>-9	No
Name of eating occasion 3	B12_occ3	A50	Not available (if B12= 0)<br>Missing	-8<br>-9	No
Start time occasion 3	B12_occ3_start	Date	00:00-24:00<br>Not available (if B12= 0)<br>Missing	00.00-24.00<br>-8<br>-9	No
End time occasion 3	B12_occ3_end	Date	00:00-24:00<br>Not available (if B12= 0)<br>Missing	00.00-24.00<br>-8<br>-9	No
Name of eating occasion 4	B12_occ4	A50	Not available (if B12= 0)<br>Missing	-8<br>-9	No
Start time occasion 4	B12_occ4_start	Date	00:00-24:00<br>Not available (if B12= 0)<br>Missing	00.00-24.00<br>-8<br>-9	No
End time occasion 4	B12_occ4_end	Date	00:00-24:00<br>Not available (if B12= 0)<br>Missing	00.00-24.00<br>-8<br>-9	No
B13. Are there scheduled times for recess between lessons for 5-6th graders?	B13	N1	No<br>Yes (please fill in table below)<br>Missing	0<br>1<br>-9	No
Start time recess 1	B13_rec1_start	Date	00:00-24:00<br>Not available (if B13= 0)<br>Missing	00.00-24.00<br>-8<br>-9	No
End time recess 1	B13_rec1_end	Date	00:00-24:00<br>Not available (if B13= 0)<br>Missing	00.00-24.00<br>-8<br>-9	No
Start time recess 2	B13_rec2_start	Date	00:00-24:00<br>Not available (if B13= 0)<br>Missing	00.00-24.00<br>-8<br>-9	No
End time recess 2	B13_rec2_end	Date	00:00-24:00<br>Not available (if B13= 0)<br>Missing	00.00-24.00<br>-8<br>-9	No
Start time recess 3	B13_rec3_start	Date	00:00-24:00<br>Not available (if B13= 0)<br>Missing	00.00-24.00<br>-8<br>-9	No
End time recess 3	B13_rec3_end	Date	00:00-24:00<br>Not available (if B13= 0)<br>Missing	00.00-24.00<br>-8<br>-9	No
Start time recess 4	B13_rec4_start	Date	00:00-24:00<br>Not available (if B13= 0)<br>Missing	00.00-24.00<br>-8<br>-9	No
End time recess 4	B13_rec4_end	Date	00:00-24:00<br>Not available (if B13= 0)	00.00-24.00<br>-8	No

			Missing	-9	
B14. How many lessons of required physical education are scheduled per week for the 5th-6th graders in your school?					
5th graders	B14_5th	N1	0/not required<br>1 lessons<br>2 lessons<br>3 lessons<br>4 lessons<br>5 or more lessons<br>Missing	0<br>1<br>2<br>3<br>4<br>5<br>-9	No
6th graders	B14_6th	N1	0/not required<br>1 lessons<br>2 lessons<br>3 lessons<br>4 lessons<br>5 or more lessons<br>Missing	0<br>1<br>2<br>3<br>4<br>5<br>-9	No
B15. What is the scheduled time per lesson of physical education for the 5th - 6th graders?	B15	N1	30 minutes or less<br>30-45 minutes<br>46-60 minutes<br>61-75 minutes<br>76-90 minutes<br>More than 90 minutes<br>Missing	1<br>2<br>3<br>4<br>5<br>6<br>-9	No
B16. Does your school regularly (i.e. weekly) offer or practice any of the following additional opportunities for 5th - 6th graders to be physically active?					
a. Brief teacher organized physical activities in lessons other than physical education	B16_a	N1	No<br>Yes<br>Missing	0<br>1<br>-9	No
b. Organised sport activities before or after the school day (i.e. school sports team, after-school activities)	B16_b	N1	No<br>Yes<br>Missing	0<br>1<br>-9	No
B17. From where does the majority of 5-6th graders get the food/drinks that they consume during the school day? (Please check all that apply) Think about main meals and snacks as you described them in question 12.					
Home (i.e. bagged lunch)	B17_a	N1	No<br>Yes, for meals<br>Yes, for snacks<br>Missing	0<br>1<br>2<br>-9	No
Shops etc around the school (i.e. bought on the way to school or in recess during the school day)	B17_b	N1	No<br>Yes, for meals<br>Yes, for snacks<br>Missing	0<br>1<br>2<br>-9	No
Subscription programs offered through school (i.e. milk, fruit, sandwiches)	B17_c	N1	No<br>Yes, for meals<br>Yes, for snacks<br>Missing	0<br>1<br>2<br>-9	No
School canteen	B17_d	N1	No<br>Yes, for meals	0<br>1	No

			Yes, for snacks<br>Missing	2<br>-9	
School shop/kiosk	B17_e	N1	No<br>Yes, for meals<br>Yes, for snacks<br>Missing	0<br>1<br>2<br>-9	No
Vending machines on the school premises	B17_f	N1	No<br>Yes, for meals<br>Yes, for snacks<br>Missing	0<br>1<br>2<br>-9	No
B18. How are the meals/snacks obtained at school paid for? (Please check all that apply)					
Parents prepay - in full	B18_a	N1	Meals<br>Snacks<br>Not applicable<br>Missing	1<br>2<br>0<br>-9	No
Parents prepay - but partly subsidized (by school/government)	B18_b	N1	Meals<br>Snacks<br>Not applicable<br>Missing	1<br>2<br>0<br>-9	No
Parent pay afterwards (by invoice)	B18_c	N1	Meals<br>Snacks<br>Not applicable<br>Missing	1<br>2<br>0<br>-9	No
Pupil/parent pay at point of purchase - in full	B18_d	N1	Meals<br>Snacks<br>Not applicable<br>Missing	1<br>2<br>0<br>-9	No
Pupil/parent pay at point of purchase - but partly subsidized (by school/government)	B18_e	N1	Meals<br>Snacks<br>Not applicable<br>Missing	1<br>2<br>0<br>-9	No
School pay - in full (through own budget)	B18_f	N1	Meals<br>Snacks<br>Not applicable<br>Missing	1<br>2<br>0<br>-9	No
School pay – but subsidized by government (local/national)	B18_g	N1	Meals<br>Snacks<br>Not applicable<br>Missing	1<br>2<br>0<br>-9	No
Local/national government pay – in full	B18_h	N1	Meals<br>Snacks<br>Not applicable<br>Missing	1<br>2<br>0<br>-9	No
Private companies pay – in full	B18_i	N1	Meals<br>Snacks<br>Not applicable<br>Missing	1<br>2<br>0<br>-9	No
Private companies pay – in part	B18_j	N1	Meals<br>Snacks<br>Not applicable<br>Missing	1<br>2<br>0<br>-9	No
Offered for free/subsidized price to pupils from low-income families	B18_k	N1	Meals<br>Snacks<br>Not applicable<br>Missing	1<br>2<br>0<br>-9	No

Section C: Other organizational factors related to eating/drinking and physical activity					
**Question**	**Variable name**	**Type**	**Value**	**Code**	**Compulsory**
C19. Please, indicate which of the following eating/drinking practices you consider part of your school's (daily) routine. (Y=Yes, P/S= partly or sometimes, N=No, NA = not applicable)					
In our school we:					
Provide easy access to fresh drinking water at all times	C19_a	N1	Yes<br>Partly or sometimes<br>No<br>Not applicable<br>Missing	1<br>2<br>3<br>4<br>-9	No
Comment	C19_a_C	A250			No
Apply nutritional guidelines for what foods/drinks may be offered in the canteen	C19_b	N1	Yes<br>Partly or sometimes<br>No<br>Not applicable<br>Missing	1<br>2<br>3<br>4<br>-9	No
Comment	C19_b_C	A250			No
Apply nutritional guidelines for what foods/drinks may be sold in the school shop/kiosk	C19_c	N1	Yes<br>Partly or sometimes<br>No<br>Not applicable<br>Missing	1<br>2<br>3<br>4<br>-9	No
Comment	C19_c_C	A250			No
Apply nutritional guidelines for what foods/drinks may be sold in vending machines	C19_d	N1	Yes<br>Partly or sometimes<br>No<br>Not applicable<br>Missing	1<br>2<br>3<br>4<br>-9	No
Comment	C19_d_C	A250			No
Prohibit that pupils bring unhealthy foods/drinks to school on a regular school day	C19_e	N1	Yes<br>Partly or sometimes<br>No<br>Not applicable<br>Missing	1<br>2<br>3<br>4<br>-9	No
Comment	C19_e_C	A250			No
Allow pupils to leave school during any or all of the recesses	C19_f	N1	Yes<br>Partly or sometimes<br>No<br>Not applicable<br>Missing	1<br>2<br>3<br>4<br>-9	No
Comment	C19_f_C	A250			No
Provide adequate eating facilities (i.e. clean, access to water to wash hands, waste bins)	C19_g	N1	Yes<br>Partly or sometimes<br>No<br>Not applicable<br>Missing	1<br>2<br>3<br>4<br>-9	No
Comment	C19_g_C	A250			No
Provide adult supervision during scheduled snacks/meal times	C19_h	N1	Yes<br>Partly or sometimes<br>No<br>Not applicable<br>Missing	1<br>2<br>3<br>4<br>-9	No
Comment	C19_h_C	A250			No

Encourage teachers and other staff to act as role models for healthy eating	C19_i	N1	Yes<br>Partly or sometimes<br>No<br>Not applicable<br>Missing	1<br>2<br>3<br>4<br>-9	No
Comment	C19_i_C	A250			No
Encourage pupils to eat and drink healthy foods and drinks	C19_j	N1	Yes<br>Partly or sometimes<br>No<br>Not applicable<br>Missing	1<br>2<br>3<br>4<br>-9	No
Comment	C19_j_C	A250			No
Allow pupils to eat whenever they want	C19_k	N1	Yes<br>Partly or sometimes<br>No<br>Not applicable<br>Missing	1<br>2<br>3<br>4<br>-9	No
Comment	C19_k_C	A250			No
Limit access to canteen to snack/meal times only	C19_l	N1	Yes<br>Partly or sometimes<br>No<br>Not applicable<br>Missing	1<br>2<br>3<br>4<br>-9	No
Comment	C19_l_C	A250			No
Limit access to school shop/kiosk to snack/meal times only	C19_m	N1	Yes<br>Partly or sometimes<br>No<br>Not applicable<br>Missing	1<br>2<br>3<br>4<br>-9	No
Comment	C19_m_C	A250			No
Limit access to vending machines to snack/meal times only	C19_n	N1	Yes<br>Partly or sometimes<br>No<br>Not applicable<br>Missing	1<br>2<br>3<br>4<br>-9	No
Comment	C19_n_C	A250			No
Provide healthy food/drink options at social/sporting events	C19_o	N1	Yes<br>Partly or sometimes<br>No<br>Not applicable<br>Missing	1<br>2<br>3<br>4<br>-9	No
Comment	C19_o_C	A250			No
Provide healthy food/drink options at parent meetings	C19_p	N1	Yes<br>Partly or sometimes<br>No<br>Not applicable<br>Missing	1<br>2<br>3<br>4<br>-9	No
Comment	C19_p_C	A250			No
Provide healthy food/drink options at teacher trainings and other staff meetings	C19_q	N1	Yes<br>Partly or sometimes<br>No<br>Not applicable<br>Missing	1<br>2<br>3<br>4<br>-9	No
Comment	C19_q_C	A250			No
Allow marketing (i.e. posters, material, events) of unhealthy foods/drinks at/through school	C19_r	N1	Yes<br>Partly or sometimes<br>No<br>Not applicable<br>Missing	1<br>2<br>3<br>4<br>-9	No

Comment	C19_r_C	A250			No
Prohibit sale of unhealthy foods as fund raising during school days	C19_s	N1	Yes<br>Partly or sometimes<br>No<br>Not applicable<br>Missing	1<br>2<br>3<br>4<br>-9	No
Comment	C19_s_C	A250			No
Prohibit use of unhealthy food/drinks as rewards by teachers and other staff	C19_t	N1	Yes<br>Partly or sometimes<br>No<br>Not applicable<br>Missing	1<br>2<br>3<br>4<br>-9	No
Comment	C19_t_C	A250			No
Teach about food, nutrition and health as part of the curriculum	C19_u	N1	Yes<br>Partly or sometimes<br>No<br>Not applicable<br>Missing	1<br>2<br>3<br>4<br>-9	No
Comment	C19_u_C	A250			No
Teach practical cooking skills as part of the curriculum	C19_v	N1	Yes<br>Partly or sometimes<br>No<br>Not applicable<br>Missing	1<br>2<br>3<br>4<br>-9	No
Comment	C19_v_C	A250			No
Address the importance of everyday healthy eating at parent meetings	C19_w	N1	Yes<br>Partly or sometimes<br>No<br>Not applicable<br>Missing	1<br>2<br>3<br>4<br>-9	No
Comment	C19_w_C	A250			No
Address the importance of everyday healthy eating in written information to parents	C19_x	N1	Yes<br>Partly or sometimes<br>No<br>Not applicable<br>Missing	1<br>2<br>3<br>4<br>-9	No
Comment	C19_x_C	A250			No
C20. Please, indicate which of the following physical activity practices you consider part of your school's (daily) routine. (Y=Yes, P/S= partly or sometimes, N=No, NA = not applicable)					
In our school we:					
Encourage biking/walking to school	C20_a	N1	Yes<br>Partly or sometimes<br>No<br>Not applicable<br>Missing	1<br>2<br>3<br>4<br>-9	No
Comment	C20_a_C	A250			No
Provide bike racks or a designated area for bike parking	C20_b	N1	Yes<br>Partly or sometimes<br>No<br>Not applicable<br>Missing	1<br>2<br>3<br>4<br>-9	No
Comment	C20_b_C	A250			No
Work to ensure traffic safety at start/end of the school day	C20_c	N1	Yes<br>Partly or sometimes	1<br>2	No

			No<br>Not applicable<br>Missing	3<br>4<br>-9	
Comment	C20_c_C	A250			No
Require pupils to be outside during recess	C20_d	N1	Yes<br>Partly or sometimes<br>No<br>Not applicable<br>Missing	1<br>2<br>3<br>4<br>-9	No
Comment	C20_d_C	A250			No
Allow pupils to use own equipment (balls, ropes etc) during recess	C20_e	N1	Yes<br>Partly or sometimes<br>No<br>Not applicable<br>Missing	1<br>2<br>3<br>4<br>-9	No
Comment	C20_e_C	A250			No
Provide access to sports equipment (balls, ropes etc) during recess	C20_f	N1	Yes<br>Partly or sometimes<br>No<br>Not applicable<br>Missing	1<br>2<br>3<br>4<br>-9	No
Comment	C20_f_C	A250			No
Provide adult/teacher supervision of school grounds during recess	C20_g	N1	Yes<br>Partly or sometimes<br>No<br>Not applicable<br>Missing	1<br>2<br>3<br>4<br>-9	No
Comment	C20_g_C	A250			No
Encourage pupils to play or be physically active during recess	C20_h	N1	Yes<br>Partly or sometimes<br>No<br>Not applicable<br>Missing	1<br>2<br>3<br>4<br>-9	No
Comment	C20_h_C	A250			No
Teach about physical activity and health as part of the curriculum	C20_i	N1	Yes<br>Partly or sometimes<br>No<br>Not applicable<br>Missing	1<br>2<br>3<br>4<br>-9	No
Comment	C20_i_C	A250			No
Teach skills to practice specific sports as part of the curriculum	C20_j	N1	Yes<br>Partly or sometimes<br>No<br>Not applicable<br>Missing	1<br>2<br>3<br>4<br>-9	No
Comment	C20_j_C	A250			No
Require physical education teachers to be certified/specialists in physical education	C20_k	N1	Yes<br>Partly or sometimes<br>No<br>Not applicable<br>Missing	1<br>2<br>3<br>4<br>-9	No
Comment	C20_k_C	A250			No
Require pupils to be dressed for physical activity in physical education lessons	C20_l	N1	Yes<br>Partly or sometimes<br>No<br>Not applicable<br>Missing	1<br>2<br>3<br>4<br>-9	No
Comment	C20_l_C	A250			No
Provide clean and separate	C20_m	N1	Yes	1	No

changing rooms with showers			Partly or sometimes<br>No<br>Not applicable<br>Missing	2<br>3<br>4<br>-9	
Comment	C20_m_C	A250			No
Require pupils to shower after physical education lessons	C20_n	N1	Yes<br>Partly or sometimes<br>No<br>Not applicable<br>Missing	1<br>2<br>3<br>4<br>-9	No
Comment	C20_n_C	A250			No
Require a note from parents for single absences from physical education	C20_o	N1	Yes<br>Partly or sometimes<br>No<br>Not applicable<br>Missing	1<br>2<br>3<br>4<br>-9	No
Comment	C20_o_C	A250			No
Require a note from a doctor for long term absences from physical education	C20_p	N1	Yes<br>Partly or sometimes<br>No<br>Not applicable<br>Missing	1<br>2<br>3<br>4<br>-9	No
Comment	C20_p_C	A250			No
Provide alternative physical activities in physical education for pupils with special needs	C20_q	N1	Yes<br>Partly or sometimes<br>No<br>Not applicable<br>Missing	1<br>2<br>3<br>4<br>-9	No
Comment	C20_q_C	A250			No
Provide parents with feedback on their child's development in physical education	C20_r	N1	Yes<br>Partly or sometimes<br>No<br>Not applicable<br>Missing	1<br>2<br>3<br>4<br>-9	No
Comment	C20_r_C	A250			No
Give physical education homework	C20_s	N1	Yes<br>Partly or sometimes<br>No<br>Not applicable<br>Missing	1<br>2<br>3<br>4<br>-9	No
Comment	C20_s_C	A250			No
Include physical activity in lessons other than physical education	C20_t	N1	Yes<br>Partly or sometimes<br>No<br>Not applicable<br>Missing	1<br>2<br>3<br>4<br>-9	No
Comment	C20_t_C	A250			No
Allow general use of school grounds (incl. outdoor facilities) before/after the school day	C20_u	N1	Yes<br>Partly or sometimes<br>No<br>Not applicable<br>Missing	1<br>2<br>3<br>4<br>-9	No
Comment	C20_u_C	A250			No
Encourage teachers and other staff to act as role models for physical activity	C20_v	N1	Yes<br>Partly or sometimes<br>No<br>Not applicable<br>Missing	1<br>2<br>3<br>4<br>-9	No
Comment	C20_v_C	A250			No

Address the importance of everyday physical activity at parent meetings	C20_w	N1	Yes<br>Partly or sometimes<br>No<br>Not applicable<br>Missing	1<br>2<br>3<br>4<br>-9	No
Comment	C20_w_C	A250			No
Address the importance of everyday physical activity in written information to parents	C20_x	N1	Yes<br>Partly or sometimes<br>No<br>Not applicable<br>Missing	1<br>2<br>3<br>4<br>-9	No
Comment	C20_x_C	A250			No
C21. In which forums or with whom have eating/drinking practices been discussed at your school in the past 2 years?					
Teacher/staff meetings	C21_a	N1	No<br>Yes, to some extent<br>Yes, to a great extent<br>Missing	0<br>1<br>2<br>-9	No
Parent meetings	C21_b	N1	No<br>Yes, to some extent<br>Yes, to a great extent<br>Missing	0<br>1<br>2<br>-9	No
With pupils	C21_c	N1	No<br>Yes, to some extent<br>Yes, to a great extent<br>Missing	0<br>1<br>2<br>-9	No
With school nurse/doctor	C21_d	N1	No<br>Yes, to some extent<br>Yes, to a great extent<br>Missing	0<br>1<br>2<br>-9	No
Other	C21_e	N1	No<br>Yes, to some extent<br>Yes, to a great extent<br>Missing	0<br>1<br>2<br>-9	No
C22. In which forums or with whom have physical activity practices for this school been discussed in the past 2 years?					
Teacher/staff meetings	C22_a	N1	No<br>Yes, to some extent<br>Yes, to a great extent<br>Missing	0<br>1<br>2<br>-9	No
Parent meetings	C22_b	N1	No<br>Yes, to some extent<br>Yes, to a great extent<br>Missing	0<br>1<br>2<br>-9	No
With pupils	C22_c	N1	No<br>Yes, to some extent<br>Yes, to a great extent<br>Missing	0<br>1<br>2<br>-9	No
With school nurse/doctor	C22_d	N1	No<br>Yes, to some extent<br>Yes, to a great extent<br>Missing	0<br>1<br>2<br>-9	No
Other	C22_e	N1	No<br>Yes, to some extent<br>Yes, to a great extent<br>Missing	0<br>1<br>2<br>-9	No

C23a. Are any of the practices discussed stated in written documents?					
Yes, in a school health policy	C23a_a	N1	Not selected<br>Selected<br>Missing	0<br>1<br>-9	No
Yes, in a school food policy	C23a_b	N1	Not selected<br>Selected<br>Missing	0<br>1<br>-9	No
Yes, in a school physical activity policy	C23a_c	N1	Not selected<br>Selected<br>Missing	0<br>1<br>-9	No
Yes, other	C23a_d	N1	Not selected<br>Selected<br>Missing	0<br>1<br>-9	No
No	C23a_e	N1	Not selected<br>Selected<br>Missing	0<br>1<br>-9	No
C23b. For how long has the school had these policy documents?					
Health	C23b_health	N1	Less than a year<br>1-2 years<br>3-4 years<br>5 years or more<br>Not available (if C23a_e= 1)<br>Missing	0<br>1<br>2<br>3<br>-8<br>-9	No
Food	C23b_food	N1	Less than a year<br>1-2 years<br>3-4 years<br>5 years or more<br>Not available (if C23a_e= 1)<br>Missing	0<br>1<br>2<br>3<br>-8<br>-9	No
Physical activity	C23b_PA	N1	Less than a year<br>1-2 years<br>3-4 years<br>5 years or more<br>Not available (if C23a_e= 1)<br>Missing	0<br>1<br>2<br>3<br>-8<br>-9	No
**Section D: Social factors related to healthy eating and physical activity**					
**Question**	**Variable name**	**Type**	**Value**	**Code**	**Compulsory**
D24. Rate to what extent teachers/other adults at your school act as role models by eating healthy foods/drinks.	D24	N1	Very large<br>Large<br>Medium<br>Small<br>Missing	1<br>2<br>3<br>4<br>-9	No
D25. Rate to what extent healthy foods/drinks are promoted at your school's social/sporting events.	D25	N1	Very large<br>Large<br>Medium<br>Small<br>Missing	1<br>2<br>3<br>4<br>-9	No
D26. Rate to what the extent the majority of parents at your school support healthy eating in school.	D26	N1	Very large<br>Large<br>Medium<br>Small<br>Missing	1<br>2<br>3<br>4<br>-9	No
D27. Rate to what extent teachers/other adults at your school act as role models by being physically active.	D27	N1	Very large<br>Large<br>Medium	1<br>2<br>3	No

			Small<br>Missing	4<br>-9	
D28. Rate to what extent play or physical activity during recess is promoted at your school.	D28	N1	Very large<br>Large<br>Medium<br>Small<br>Missing	1<br>2<br>3<br>4<br>-9	No
D29. Rate to what extent the majority of parents at your school support physical activity to/from and in school.	D29	N1	Very large<br>Large<br>Medium<br>Small<br>Missing	1<br>2<br>3<br>4<br>-9	No
D30. Rate to what extent promoting healthy eating and/or physical activity is regarded as important at your school.	D30	N1	Very large<br>Large<br>Medium<br>Small<br>Missing	1<br>2<br>3<br>4<br>-9	No
D31. Rate to what extent the school health services promote healthy eating and/or physical activity at your school.	D31	N1	Very large<br>Large<br>Medium<br>Small<br>Missing	1<br>2<br>3<br>4<br>-9	No
D32. Rate to what extent you personally regard promoting healthy eating and/or physical activity as important for schools.	D32	N1	Very large<br>Large<br>Medium<br>Small<br>Missing	1<br>2<br>3<br>4<br>-9	No
**Section E: Economic factors related to eating/drinking and physical activity**					
**Question**	**Variable name**	**Type**	**Value**	**Code**	**Compulsory**
E33. Please, indicate which of the following economic factors/sponsorships have been used in your school in the past 2 years. (Y=Yes, P/S= partly or sometimes, N=No, NA = not applicable)					
In our school we have:					
Free/subsidized milk subscription program for all	E33_a	N1	Yes<br>Partly or sometimes<br>No<br>Not applicable<br>Missing	1<br>2<br>3<br>4<br>-9	No
Comment	E33_a	A250			No
Free/subsidized fruit/vegetable subscription program for all	E33_b	N1	Yes<br>Partly or sometimes<br>No<br>Not applicable<br>Missing	1<br>2<br>3<br>4<br>-9	No
Comment	E33_b	A250			No
Free/subsidized lunch for all	E33_c	N1	Yes<br>Partly or sometimes<br>No<br>Not applicable<br>Missing	1<br>2<br>3<br>4<br>-9	No
Comment	E33_c	A250			No
Free/subsidized snacks for all	E33_d	N1	Yes<br>Partly or sometimes<br>No<br>Not applicable	1<br>2<br>3<br>4	No

			Missing	-9	
Comment	E33_d	A250			No
Reduced prices on healthy options in canteen	E33_e	N1	Yes<br>Partly or sometimes<br>No<br>Not applicable<br>Missing	1<br>2<br>3<br>4<br>-9	No
Comment	E33_e	A250			No
Reduced prices on healthy options in shop/kiosk	E33_f	N1	Yes<br>Partly or sometimes<br>No<br>Not applicable<br>Missing	1<br>2<br>3<br>4<br>-9	No
Comment	E33_f	A250			No
Reduced prices on healthy options in vending machines	E33_g	N1	Yes<br>Partly or sometimes<br>No<br>Not applicable<br>Missing	1<br>2<br>3<br>4<br>-9	No
Comment	E33_g	A250			No
Offered package deals on healthy options (i.e. healthy food with healthy drink)	E33_h	N1	Yes<br>Partly or sometimes<br>No<br>Not applicable<br>Missing	1<br>2<br>3<br>4<br>-9	No
Comment	E33_h	A250			No
Used coupon systems for healthy options (i.e. buy 5 – get the 6th for free)	E33_i	N1	Yes<br>Partly or sometimes<br>No<br>Not applicable<br>Missing	1<br>2<br>3<br>4<br>-9	No
Comment	E33_i	A250			No
Increased prices on unhealthy options in canteen/shop/kiosk/vending machines	E33_j	N1	Yes<br>Partly or sometimes<br>No<br>Not applicable<br>Missing	1<br>2<br>3<br>4<br>-9	No
Comment	E33_j	A250			No
Obtained sponsorship for healthy foods/drinks to social/sporting events	E33_k	N1	Yes<br>Partly or sometimes<br>No<br>Not applicable<br>Missing	1<br>2<br>3<br>4<br>-9	No
Comment	E33_k	A250			No
Obtained sponsorship for unhealthy foods/drinks to social/sporting events	E33_l	N1	Yes<br>Partly or sometimes<br>No<br>Not applicable<br>Missing	1<br>2<br>3<br>4<br>-9	No
Comment	E33_l	A250			No
Obtained sponsorship for healthy foods/drinks on regular school days	E33_m	N1	Yes<br>Partly or sometimes<br>No<br>Not applicable<br>Missing	1<br>2<br>3<br>4<br>-9	No
Comment	E33_m	A250			No
Obtained sponsorship for unhealthy foods/drinks on regular school days	E33_n	N1	Yes<br>Partly or sometimes<br>No	1<br>2<br>3	No

			Not applicable<br>Missing	4<br>-9	
Comment	E33_n	A250			No
Applied for funding that required the implementation of certain healthy eating and/or physical activity practices	E33_o	N1	Yes<br>Partly or sometimes<br>No<br>Not applicable<br>Missing	1<br>2<br>3<br>4<br>-9	No
Comment	E33_o	A250			No
Obtained sponsorship to buy sports equipment for recess	E33_p	N1	Yes<br>Partly or sometimes<br>No<br>Not applicable<br>Missing	1<br>2<br>3<br>4<br>-9	No
Comment	E33_p	A250			No
Obtained sponsorship to maintain/renovate the school ground (incl. play areas)	E33_q	N1	Yes<br>Partly or sometimes<br>No<br>Not applicable<br>Missing	1<br>2<br>3<br>4<br>-9	No
Comment	E33_q	A250			No
Obtained sponsorship to maintain/renovate outdoor or indoor sport facilities	E33_r	N1	Yes<br>Partly or sometimes<br>No<br>Not applicable<br>Missing	1<br>2<br>3<br>4<br>-9	No
Comment	E33_r	A250			No
Obtained sponsorship to offer extra physical activity during school hours	E33_s	N1	Yes<br>Partly or sometimes<br>No<br>Not applicable<br>Missing	1<br>2<br>3<br>4<br>-9	No
Comment	E33_s	A250			No
Obtain sponsorship to offer after school sports programs	E33_t	N1	Yes<br>Partly or sometimes<br>No<br>Not applicable<br>Missing	1<br>2<br>3<br>4<br>-9	No
Comment	E33_t	A250			No
E34a. Has the school participated in national or regional campaigns with the aim of promoting healthy diets in the past 2 years, and involving any form of (chance of) reward?	E34_a	N1	Yes, a campaign involving a reward for all pupils on the school<br>Yes, a campaign involving a reward for a group of pupils on the school<br>Yes, a campaign involving a reward for single pupils<br>Yes, a campaign involving a reward for the school<br>The school has participated in campaign(s) without such reward schemes<br>No, the school has not participated in such campaigns during the last 2 years<br>Don't know<br>Missing	1<br>2<br>3<br>4<br>5<br>6<br>7<br>-9	No
E34b. Has the school participated in national or regional campaigns with the aim of promoting physical activity in	E34_b	N1	Yes, a campaign involving a reward for all pupils on the school	1	No

the past 2 years, involving any form of (chance of) reward?			Yes, a campaign involving a reward for a group of pupils on the school<br>Yes, a campaign involving a reward for single pupils<br>Yes, a campaign involving a reward for the school<br>The school has participated in campaign(s) without such reward schemes<br>No, the school has not participated in such campaigns during the last 2 years<br>Don't know<br>Missing	2<br>3<br>4<br>5<br>6<br>7<br>-9	
Please provide any comments you might have to the questionnaire and its content	Comment	A500			No
